# The varieties of contemplative experience: A mixed-methods study of meditation-related challenges in Western Buddhists

**DOI:** 10.1371/journal.pone.0176239

**Published:** 2017-05-24

**Authors:** Jared R. Lindahl, Nathan E. Fisher, David J. Cooper, Rochelle K. Rosen, Willoughby B. Britton

**Affiliations:** 1 Cogut Center for the Humanities, Brown University, Providence, Rhode Island, United States of America; 2 Department of Religious Studies, University of California, Santa Barbara, California, United States of America; 3 Department of Psychiatry and Human Behavior, Brown University Medical School, Providence, Rhode Island, United States of America; 4 Department of Behavioral and Social Sciences, Brown University School of Public Health, Providence, Rhode Island, United States of America; Virginia Commonwealth University, UNITED STATES

## Abstract

Buddhist-derived meditation practices are currently being employed as a popular form of health promotion. While meditation programs draw inspiration from Buddhist textual sources for the benefits of meditation, these sources also acknowledge a wide range of other effects beyond health-related outcomes. The Varieties of Contemplative Experience study investigates meditation-related experiences that are typically underreported, particularly experiences that are described as challenging, difficult, distressing, functionally impairing, and/or requiring additional support. A mixed-methods approach featured qualitative interviews with Western Buddhist meditation practitioners and experts in Theravāda, Zen, and Tibetan traditions. Interview questions probed meditation experiences and influencing factors, including interpretations and management strategies. A follow-up survey provided quantitative assessments of causality, impairment and other demographic and practice-related variables. The content-driven thematic analysis of interviews yielded a taxonomy of 59 meditation-related experiences across 7 domains: cognitive, perceptual, affective, somatic, conative, sense of self, and social. Even in cases where the phenomenology was similar across participants, interpretations of and responses to the experiences differed considerably. The associated valence ranged from very positive to very negative, and the associated level of distress and functional impairment ranged from minimal and transient to severe and enduring. In order to determine what factors may influence the valence, impact, and response to any given experience, the study also identified 26 categories of influencing factors across 4 domains: practitioner-level factors, practice-level factors, relationships, and health behaviors. By identifying a broader range of experiences associated with meditation, along with the factors that contribute to the presence and management of experiences reported as challenging, difficult, distressing or functionally impairing, this study aims to increase our understanding of the effects of contemplative practices and to provide resources for mediators, clinicians, meditation researchers, and meditation teachers.

## Introduction

Buddhist meditation practices, which traditionally have been part of an extensive religious path to awakening (*bodhi*), are now in the modern Western context also a popular form of general health promotion that is simultaneously bound to and divorced from its religious roots. Inspired by Buddhist claims for the possibility of freedom from “suffering” (*dukkha*), key Buddhist doctrines and practices have been re-presented within and adapted to psychological and biomedical frameworks [1]. In this new context, Buddhist-derived meditation practices inform treatment programs such as “mindfulness-based interventions” (MBIs), which are being applied towards the alleviation of a wide range of ailments, including stress [2], addiction [3], chronic pain [4], mood disorders [5], psychiatric disorders [6], and medical conditions [7]. Such programs have also been used to improve cognitive abilities [8] and emotion regulation [9]. MBIs are being employed at institutional levels, including prisons [10, 11], the military [12–14], and in both K-12 [15–18] and higher education [19]. With more than 20 mindfulness phone apps [20], mindfulness is major contributor to the billion-dollar meditation industry [21] that serves more than 18 million meditators [22], with 1 million new meditators each year in the United States alone [23].

For its theory and practices, as well as for its legitimacy and authenticity, the mindfulness movement draws heavily from Buddhist texts and teachings [24, 25]; it also looks to studies of long-term Buddhist meditators as evidence of meditation’s potential benefits [26–29]. While these sources are often assumed to be indicative of “the effects of meditation,” the focus on positive health-related benefits represents only a narrow selection of possible effects that have been acknowledged within Buddhist traditions both past and present.

Scholars of Buddhist Studies have documented the historical processes responsible for the current alliance between Buddhism and science [30–32]. Recent scholarship has also addressed how the modern representation of Buddhism as “secular,” “empirical,” and “scientific” (or a least compatible with these modern Western values) has led to the success of the mindfulness movement within biomedical health science and the broader spiritual marketplace, which has branded mindfulness as relevant and efficacious not for the attainment of religious goals, but for a project of health, happiness, and well-being believed to have a broader, if not universal, appeal [33–36]. However, the limited focus on the benefits of meditation for physical and psychological health and well-being is a modern and largely Western creation that neither represents the diversity of meditation practices nor the range of possible or even likely effects of those practices.

Many Buddhist literary sources describe how meditation practices are expected to lead to perceptual, affective, epistemic, and behavioral shifts that lie beyond the scope of the health-related outcomes that are the concern of MBIs and associated scientific research. Biographical narratives and stages-of-the-path literature across Buddhist traditions also acknowledge periods of challenge or difficulty associated with the practice of meditation. In Tibetan Buddhist traditions, the term *nyams* refers to a wide range of “meditation experiences”—from bliss and visions to intense body pain, physiological disorders, paranoia, sadness, anger and fear—which can be a source of challenge or difficulty for the meditation practitioner [37, 38]. Certain *nyams*—in particular the triad of bliss (*bde ba*), clarity or luminosity (*gsal ba*), and non-conceptuality (*mi rtog pa*)*—*are multivalent in that in some lineages of Tibetan Buddhism they are deliberately cultivated and framed as “signs of progress,” and yet in other contexts can be dismissed as untrustworthy hindrances to genuine insight [39]. In Zen Buddhist traditions, the term *makyō* refers to a class of largely perceptual “side-effects” or “disturbing conditions” that arise during the course of practice and which are also sometimes interpreted as signs of progress [40, 41]. Zen traditions have also long acknowledged the possibility for certain practice approaches to lead to a prolonged illness-like condition known as “Zen sickness” [42] or “meditation sickness” [34]. The *Śūraṅgama Sūtra*—a classic text of Mahāyāna Buddhism—enumerates fifty deceptive or illusory experiences that are associated primarily, though not exclusively, with the practice of concentration (*samādhi*). The *Sūtra* particularly warns about pleasant experiences that lead the meditator into a false sense of spiritual progress, which results in misguided thinking and conduct [43]. Similarly, in Theravāda Buddhist traditions, progress in the practice of meditation is expected to lead to transient experiences called “corruptions of insight” (*vipassanā-upakkilesā*) on account of meditators’ tendency to confuse these blissful and euphoric states for genuine insight [44, 45]. Some modern accounts also include reports of monks becoming “mentally unstable” in the wake of such states [46]. Other stages of practice, in particular some of the “insight knowledges” (*vipassanā-ñāṇa*), are presented as being particularly challenging, especially in modern Asian sources [47, 48].

Biographical narratives and stages-of-the-path textual sources cannot necessarily be taken as straightforward descriptions of the experiences of past Buddhists, as such texts often also have prescriptive or polemical inclinations [49, 50]. Furthermore, the privileging of *experiences* as central to meditation may in some cases reflect modern sensibilities rather than Buddhist concerns throughout the ages [51]. Nevertheless, Buddhist-derived meditation practices are often presented in the West in terms of their non-religious health-related outcomes with little to no attention given to the possibility of the broader range of effects suggested by both traditional Buddhist sources as well as modern American Buddhist authors [52, 53]. Given that some of these effects might even run counter to the dominant paradigm of health and well-being, it is critical that the range of effects associated with Buddhist meditation be investigated in the modern Western context. In particular, we need to know: What is the range of effects associated with the practice of meditation in this context? And what effects do people report as unexpected, challenging, difficult, or distressing? Answering these questions will increase our knowledge of how Buddhist meditation is understood and practiced in the West, the type of support structures that are needed for meditation-related challenges, and the potential boundary conditions of applications of meditation for health and well-being.

### Previous research on the range of effects from meditation

Beyond the extensive literature on the positive effects of meditation for physical, emotional, and mental health and well-being, studies of other meditation effects fall into two broad categories: 1) studies of side effects or adverse effects, and 2) research on anomalous, spiritual, mystical, or religious experience. The phenomena covered in these two literatures are not necessarily distinct. Rather, the difference in appraisal often reflects the disciplinary perspective and methodological approach of the researchers involved, as well as the social contexts of the populations being studied. Providing a comprehensive review of studies on the range of phenomena associated with anomalous, spiritual, mystical, or religious experiences is beyond the scope of this paper. Instead, this section focuses specifically on recent clinical, experimental, and qualitative research on the effects of Buddhist or Buddhist-based meditation practices.

Meditation-related effects that are not health-related benefits or that are reported as distressing have been classified as “side effects” or “adverse effects” (AEs), especially in clinical psychology research. While randomized clinical trials are the most reliable source for acquiring accurate information about positive effects, information about adverse effects has not been readily available for several reasons. First, the vast majority (>75%) of meditation studies do not actively assess adverse effects [2, 54]; instead, they rely solely on patients to spontaneously report any difficulties to the researchers or teachers. However, patients are unlikely to volunteer information about negative reactions to treatment without being directly asked due to the influence of authority structures and demand characteristics [55–57]. As a result, passive monitoring is thought to underestimate AE prevalence by more than 20-fold [58]. A few MBI researchers have started to actively monitor AEs either through questionnaires or through clinician interviews [59–61]. However, these are typically limited to serious AEs (life-threatening or fatal events) or “deterioration” on pre-existing clinical outcomes that require clinical attention, such as increased depression or suicidality. These AEs, as well as traumatic flashbacks, are now listed in the MBI guidelines under “risks to participants” [62].

Meditation-related adverse effects that were serious or distressing enough to warrant additional treatment have been reported in clinical and medical literature. These include reports of meditation-induced psychosis, seizures, depersonalization, mania and other forms of clinical deterioration [63–73]. Descriptions of meditation-induced depersonalization and other clinically relevant problems also appear in the APA’s Diagnostic and Statistical Manual of Mental Disorders [74–76]. While meditation-related difficulties that are serious enough to warrant treatment are acknowledged in the clinical literature, there continues to be a lack of systematic investigation into challenging meditation experiences, what causes them, and how to prevent or manage them.

Reports from meditation practitioners have also been researched within the framework of anomalous experiences. Such studies are often lab-based experiments that use inductions to create state-based changes in one or more meditators in order to explore neural correlates [77–79]. This category includes studies on changes in sense of self [78–81], changes in sense of time and space [77, 80, 82], and changes in perception [83]. Other research has investigated trait-based changes in perception following an extended period of meditation practice [27].

Similar to these experimental studies, some qualitative studies have used theory-driven approaches to anomalous experiences that aim to test a specific hypothesis rather than conduct an open-ended exploration. For example, Chen et al. (2011) [80] asked Chinese Chan and Pure Land Buddhist meditators questions based on Hood’s Mysticism Scale in order to investigate whether their experiences provided evidence for a common core of mystical experience. Through a semi-structured interview, Full et al. (2013) [83] investigated changes in perception in thirteen advanced Burmese meditation practitioners. While the interview specifically probed changes in perception and associated benefits of meditation, it lacked a complementary probe for possible negative effects or an open-ended question to report other types of experiences. In addition, the sample was mostly male, Burmese-authorized lineage holders. While valuable, these two qualitative studies of meditative experiences are limited in that they were exclusively of Asian participants, did not ask open-ended questions that allow for a broader range of experiences associated with meditation to be reported, and did not explicitly assess the role of the practitioner’s interpretative framework.

For phenomena about which little is known, qualitative studies that ask open-ended questions are the most informative and produce the richest phenomenological data about meditation-related experiences. In a pioneering project, Kornfield (1979) [84] conducted a mixed-methods study of American Buddhist meditators during a 3-month Vipassana retreat. While this study did not specifically probe challenging or difficult experiences, the open-ended query about “unusual” experiences yielded reports uncommon in the research literature, including strong negative emotions, involuntary movements, anomalous somatic sensations, and out-of-body experiences. More recently, Lomas et al. (2014) [85] asked active meditators to “recount their involvement with meditation,” and received both positive and negative reports. Meditators reported “exacerbation of psychological problems,” including anxiety and depression, “troubling experiences of self,” and “reality being challenged,” which included out-of-body experiences and in one case resulted in patient hospitalization for psychosis. However, the study was also of an all-male sample almost entirely from one community; in addition, the authors acknowledge that their sample “arguably excludes people whose experiences of meditation were so troubling as to cause them to cease practicing (since only currently active meditators were recruited).”

In one of the only prospective studies to use qualitative methods to deliberately ask about adverse effects, Shapiro (1992) [67] found that 63% of meditators on an intensive Vipassana retreat reported at least one adverse effect, with 7.4% reporting effects negative enough to stop meditating, and one individual hospitalized for psychosis. VanderKooi (1997) [86] also specifically queried “difficulties” or “extreme states” with both meditators and the teachers who helped them navigate their experiences. In a series of case vignettes, the report emphasizes the challenges of integrating conflicting interpretive frameworks from Buddhism and psychiatry. The study also illustrates how teachers can be a rich and often under-utilized source of information about meditation-related difficulties.

While these studies make important contributions towards an interdisciplinary conversation about the range of experiences associated with meditation practices in general and Buddhist meditation practices in particular, each methodological approach is associated with its own set of limitations. Single-person case studies provide some insight into unexpected symptoms associated with meditation, but do not get at the broader range of phenomena, nor can they identify patterns that would help researchers, clinicians, and teachers to investigate what types of experiences might be expected to arise when certain causal factors are present or absent. Even larger-scale studies on groups of meditators have indicated that assessing sensitive, socially undesirable experiences such as adverse reactions to meditation requires specific probes. In clinical, experimental, and qualitative research on meditation alike, the extent to which adverse meditation experiences are reported is proportional to how specifically they are queried.

Moreover, the interpretative frameworks and appraisal processes of researchers and subjects alike also frame and impact the results and require special consideration. It is difficult to discern to what extent the classification of an experience as an “adverse effect,” a “religious experience,” or any other designation reflects a real difference in phenomenology or is a consequence of an appraisal made either by a meditator, a researcher, or both. Similar challenges affect research that attempts to compare and differentiate “mystical” or “religious” experiences from “psychopathology” [87–90] (see Discussion). Instead of attempting to impose an interpretative framework by classifying certain meditation-related experiences as “religious experiences” or alternately as forms of “psychopathology,” a better approach is to identify the interpretative frameworks held by meditation practitioners or offered by meditation experts and their impact.

### The varieties of contemplative experience research study

The purpose of the current study is to build upon the research summarized above by specifically addressing the following questions: What is the range of meditation-related effects described by Buddhist practitioners in the West? What types of experiences do they report as unexpected, challenging, difficult, distressing, or functionally impairing? What are the hypothesized causes of those experiences? What interpretations are they given by others? What impact do these experiences have on the lives of meditation practitioners? How do practitioners prevent, manage, navigate, or integrate such experiences? How do teachers guide their students through such experiences?

Through qualitative interviews with Western Buddhist meditation practitioners and meditation experts (teachers and clinicians), the Varieties of Contemplative Experience (VCE) research study attempts to investigate the broader range of experiences associated with meditation practices in the West. In order to better understand the types of experiences that tend to be under-reported in scientific research, scholarship, and the media, the VCE study intentionally queried experiences that practitioners found unexpected, difficult, distressing, or functionally impairing. Interviews also asked about the range of interpretations given to experiences, as well as the putative causes of and remedies for difficult experiences. Meditation experts were also queried for how they interpret and manage challenging experiences that are reported by their students or patients. In addition to providing descriptions of experiences and identifying potential influencing factors, the VCE study ultimately aims to provide meditators, teachers and clinicians with practical management strategies for challenging meditation-related experiences.

## Methods

### Qualitative approach and research paradigm

While quantitative methods are appropriate for "known" or “well-characterized phenomena,” qualitative methods are particularly appropriate when little is known because they “allow for identification of previously unknown processes, explanations of why and how phenomena occur, and the range of their effects” [91]. Qualitative methods are used when the goals are “developing a hypothesis or theory in the early stages of an inquiry, understanding particular cases in depth and detail, getting at meanings in context, and capturing changes in a dynamic environment” [92]. Since the VCE study aims to investigate a wide range of meditation-related experiences as well as to understand how they are integrated into practitioners’ lives, qualitative methodology was the most appropriate approach for undertaking this project because it allowed participants to describe their experiences in their own words and to offer their own explanations for them. Attention to these experiences, and the language used to describe them, is an essential formative step towards building a research strategy for further understanding them.

In addition to the fundamental principles of qualitative research, our methods are also informed by other theories and approaches. Our study design and approaches to data analysis are shaped in part by the attribution theory approach to the study of “experiences deemed religious” developed by Taves (2008 and 2009) [93, 94] to advance the cognitive science of religion. By “meditation-related experiences” we are referring to a range of effects across different domains of human experience that meditation practitioners in our study attributed to one or more types of meditation. In addition to causal attributions, practitioners and experts alike are also agents in ascribing significance, meaning, and value to meditation-related experiences, sometimes in incompatible ways. Our methods are further informed by the cultural psychiatry approach, which investigates “the ways in which psychopathology and healing are shaped by cultural knowledge and practices,” and which allows for “local nosologies” and “emic” categories of experience to inform the analysis [95].

### Context

Because the widespread practice of Buddhist meditation in the West is a relatively recent phenomenon, and because Buddhist-derived meditation practices such as “mindfulness” are increasingly being deployed in various novel settings such as schools, clinics, and hospitals, more information is needed on the range of experiences associated with the practice of Buddhist meditation in the West. To capture “ecologically valid” or “real world” scenarios, the VCE study includes practitioners from multiple traditions who engaged in various meditation practices in the context of either daily practice or intensive retreat. The relationship between traditional forms of meditation and their therapeutic applications is still being negotiated, with many advocates for a deeper relationship between interventions and traditional Buddhist theory and practice [96, 97]. Therefore, assessing the range of experiences reported by practitioners in a variety of Buddhist traditions not only provides valuable information about Buddhism as it is practiced and experienced in the West, it also has the potential to inform MBIs and other novel interventions derived from Buddhist practices.

### Participants

#### Practitioners

The VCE study recruited Buddhist meditation practitioners from across Theravāda, Zen, and Tibetan traditions. Inclusion criteria for practitioners required a minimum age of 18 years, a meditation practice in a Buddhist tradition, and the ability to report on meditation-related experience that was challenging, difficult, or was associated with significant physiological or psychological changes, including distress or impairment. Exclusion criteria for practitioners were a history of unusual psychological experiences prior to learning meditation (e.g., from substance use or mental illness) that closely resembled the experiences they associated with meditation, a mixed practice history that included a significant influence from non-Buddhist practices, or the presence of other variables (such as medical illness) that could causally account for the entire reported symptomology.

#### Experts

The VCE study also recruited meditation experts who had either taught extensively in a Buddhist tradition or who had applied Buddhist meditation in clinical settings (or both). Inclusion criteria for experts were an occupational identity as a meditation teacher in a Buddhist lineage or as a clinician working with meditation-based therapies.

### Sampling strategy

#### Subject recruiting

The overall sampling strategy was purposive, where cases are selected “based on a specific purpose rather than randomly” [98]. Specifically, we used “deviant case sampling” or “outlier sampling” where participants are selected on the extreme ends of a distribution in order to investigate under-reported phenomena, which for this study were challenging, difficult, or distressing experiences associated with meditation [99]. Recruitment employed both special case sampling [99] and snowball or chain sampling [100] to meet different aims. In order to investigate the range of meditation-related experiences that occur under standard or optimal conditions, special case sampling was used to select experienced teachers or lineage-holders from well-respected meditation centers. These teachers could provide two different kinds of interviews: interviews about their own experiences (practitioner interview) and/or experiences they had observed in their students (expert interview). Teachers who contributed interviews subsequently provided VCE study contact information to students with notable or challenging experiences, many of whom in turn provided VCE study contact information to fellow meditators. Selection of subjects proceeded iteratively to ensure a balanced gender ratio and equal representation across different Buddhist meditative traditions.

#### Sample size

Sample size was based on two stratification variables: gender and Buddhist tradition. Because we sought 10 males and 10 females for each of the three Buddhist traditions (Theravāda, Zen, and Tibetan), our target sample size was 60. After the initial 30 interviews, and again when the targeted sample was achieved, we reviewed the data, assessing the range and saturation of reported experiences, to determine if additional interviews were needed. Additionally, because initial qualitative coding proceeded while recruitment and interviewing was ongoing, this allowed us to assess saturation with respect to reported meditation experiences multiple times.

### Researcher characteristics and reflexivity

The Varieties of Contemplative Experience study was executed by an interdisciplinary research team comprised of a clinical psychologist and neuroscientist (WB), three religious studies scholars (JL, DC, NF), and a qualitative methodologist trained in both medical anthropology and behavioral medicine (RR). Interviewers had practical expertise and/or academic expertise with one or more of the three main Buddhist meditation traditions being researched. In order to establish rapport with interview subjects and to be able to engage with tradition-specific practices or terminology, interviewers were regularly matched with interview subjects practicing in the traditions in which they had one or more forms of expertise. Interviewers received training in qualitative methods and interviewing procedures, including sitting in on interviews and reviewing transcripts. The interdisciplinary nature of our team required that interpretive and disciplinary perspectives be made explicit. Any theory-driven approaches to qualitative analysis aimed to be representative of multiple interpretive and disciplinary perspectives, as well as faithful to the reports from the practitioners themselves.

### Ethical issues pertaining to human subjects

The study protocol and consent procedure was approved by the Brown University Institutional Review Board. All participants reviewed a consent form and provided informed consent before participating in the study. Documentation of written consent was waived for this study because most interviews took place remotely via telephone or video conferencing. Instead, participants provided verbal consent, which was recorded in a password-protected database on a secure server. All practitioners were de-identified, and their names were replaced with a subject ID number. Potentially identifying information, such as names of centers, spouses and geographical locations, was omitted from transcripts.

### Data collection methods

Interviews were conducted between December 2010 and March 2016 via telephone, video conferencing, or in person either by the PIs (WB, JL) or by other study personnel (NF, DC, and others). Interview duration was typically between 45 and 120 minutes. Data collection instruments were adapted iteratively to optimally balance structured and unstructured components of the interview, with the dual aim of facilitating exploration of the topic and the ability to compare data across practitioners [101]. Given the exploratory, content-driven nature of the study, both practitioner and expert interviews were semi-structured. While demographic information was often reported during the course of the interview, the importance of specific factors such as early life relationships, psychiatric history, and trauma history emerged during the course of the study. In order to ensure consistent and comparable information for all practitioners, a follow-up questionnaire was employed to gather comprehensive demographic information and detailed assessments of causality and impairment (see Additional instruments and quantitative measures).

### Measures and instruments

#### Practitioner interview protocol

Because the varieties of meditation-related experiences have not been well documented in scientific and scholarly literature, it was crucial that the study participants drive the interview content, not the researchers. Therefore, we developed a semi-structured interview protocol that emphasized open-ended questions (see S1 File). Interviews covered three general domains: phenomenology, interpretations, and remedies. Interviews also included questions about personal background and practice history.

The central part of the interview involved detailed descriptions of meditation-related experiences, or phenomenology. Questions included variants of “What kinds of experiences have you had as a result of meditation?” and “Have you had any significant experiences that were unexpected, challenging, or difficult?” When reporting on the phenomenology of their experience, practitioners were encouraged to refrain from using tradition-specific jargon such as “*jhāna*,” “*satori*,” or “*kuṇḍalinī*”; rather, if these terms arose, interviewers requested that practitioners attempt to explain those concepts in their own descriptive language. Interviewers also asked participants to describe the contextual factors associated with the onset of challenging or difficult meditation-related experiences by following-up with probing questions, such as “How much were you meditating at this time?” or “Were there any notable life circumstances at this time?” Practitioners were also asked questions about impact (variants of “How did these experiences impact your life?”) and causal attribution (“What is the reason you associate these experiences with meditation?”) unless the material had already been volunteered in the narrative report of their experiences.

Questions querying interpretations and explanatory frameworks included some variants of “How did you interpret your experiences?” and “How did other people interpret your experiences?” Practitioners were thus asked to provide their own interpretation of what happened as well as to report interpretations others provided them. Practitioners were also encouraged to identify where their own understanding of their meditation experiences deviated significantly from the interpretations given to them by teachers, texts, community members, or other authorities such as psychiatrists, friends, or relatives. Separately querying practitioners’ own interpretations of their experiences and others’ interpretations allowed us to disambiguate the range of phenomena associated with meditation from the ways in which these phenomena are interpreted—sometimes in divergent ways—in different sociocultural contexts. This allowed us to understand the role of other agents in the appraisal and meaning-making processes surrounding unexpected and difficult meditation experiences.

Practitioners were also asked to describe the conditions that led to the effects they associate with meditation through the main questions querying causality (variants of “What is the reason you associate these experiences with meditation?” and “Are there any other factors that you think may have influenced the nature or course of your meditation experiences?”). Salient contextual factors and demographic variables identified earlier in the interview were often revisited or explored in greater detail through follow-up questions.

Finally, questions concerning remedies included variants of “How did you and others respond to these experiences?” and “What was helpful and what was unhelpful for addressing or managing your experiences?” These questions identified the types of strategies and remedies practitioners attempted as well as the extent to which they were perceived as effective. An explicit aim of the study is not only to identify meditation-related experiences that have the potential to be challenging, difficult, distressing, or functionally impairing, but also to provide information about potentially effective methods for managing them.

#### Expert interview protocol

Expert practitioners were asked about their training, knowledge base, and institutional affiliations. The interview then proceeded to inquire into experiences meditation teachers or clinicians have seen in their students or patients, how they interpret these experiences, and what they think are the causes of, influences on, responses to, and remedies for these experiences (see S2 File). Because they were not reporting on their own experiences, expert interviews tended to provide much less specific data on phenomenology. As a result, expert interviews focused more on the identification of the causes of, interpretations of, and remedies for meditation-related difficulties.

### Additional instruments and quantitative measures

#### Demographics and attributes form

A follow-up questionnaire was distributed to practitioners through an online platform after all interviews were completed in order to obtain standardized answers on key questions. Items queried practitioner demographics such as personal history, meditation practice history, and type, degree, and duration of impairment. Psychiatric history queried the diagnosed or suspected presence of the ten most common psychiatric disorders in epidemiological studies, including anxiety, mood, psychotic, eating and substance abuse disorders [102, 103]. Trauma history queried the presence or absence of the most commonly experienced (“Criterion A”) traumas, including physical/sexual/emotional abuse or neglect; loss, injury or death of a family member or caregiver (as a child); and experience or witnessing of life-threatening injury/illness, rape, violence, death, or warfare (at any age) [104–106]. Additional items queried include influencing factors such as types of support from teachers, therapists, or friends; the degree of helpfulness of specific remedies or treatment modalities; meditation causality (see below); and severity of distress and impairment (see below).

#### Causality assessment

In the current study, causal attribution to meditation was assessed according to the causality assessment criteria that regulatory agencies such as the World Health Organization (WHO), the Federal Drug Administration (FDA), and the National Institutes of Health (NIH) use to make health policy decisions [107–110]. These criteria are designed to assess causality of treatment-related adverse events in individual cases in the absence of prospective or epidemiological (base rate) data. Thirteen standard causality criteria typically include 1) prior published reports, 2) expert judgment, 3) subjective attribution, 4) temporal proximity (challenge), 5) exacerbation, 6) de-challenge, 7) re-challenge, 8) specificity, 9–11) three types of consistency, 12) biological gradient, and 13) linkage to known biological mechanisms [108, 111–117].

The current study assessed the first 11 criteria. Prior published reports included case reports or studies about unusual or challenging meditation-related experiences in the scientific or clinical literature. Expert judgment was derived from interviews with 32 meditation teachers and clinicians. The following six criteria were assessed as part of the demographics and attributes follow-up questionnaire or, in the case of non-responders, extracted from the interview transcript: 1) causal attribution to meditation by the subject (subjective attribution); 2) temporal proximity to (either during or following) meditation practice (challenge); 3) exacerbation of pre-existing symptoms following meditation; 4) occurrence on more than one occasion (consistency); 5) decrease when practice is reduced (de-challenge); 6) re-appearance when practice is repeated (re-challenge). A causality score was calculated as the sum of endorsements of these six criteria. Using standard guidelines [118], a score of two or greater, signifying “possibly related,” was the cutoff for inclusion.

Specificity—or the likelihood of a cause other than meditation—was evaluated at two levels. If an alternative cause such as medical illness or pre-existing psychological conditions could have wholly accounted for the experiences reported, then these subjects failed to meet causality criteria and were excluded. In the remaining practitioners, specific experiences that could not be directly linked to meditation or that were attributed to other causes (e.g., drug use, prior psychiatric or medical history, or a period in life prior to learning meditation) were not coded for phenomenology (see below: Phenomenology coding).

The consistency criterion was assessed at three levels: intra-subjective, inter-subjective, and cross-modal. Intra-subjective consistency refers to the same or a similar experience occurring in close temporal proximity (during or following) to meditation on more than one occasion within the same individual. Inter-subjective consistency refers to the same or a similar experience occurring during or following meditation in multiple individuals. Cross-modal consistency refers to different classes of sources (practitioners and experts) reporting the same or a similar experience during or following meditation (i.e. source triangulation).

#### Severity assessment

Degree of associated distress and impairment were scored according to standard severity criteria for adverse events, which are based upon a combination of duration, distress, and functional impairment [118, 119]. Impairment was defined as difficulty engaging in one’s usual activities, whether social, occupational, or leisure. “Mild impairment” was defined as mild or transient distress and less than 25% reduction in usual functioning. “Moderate impairment” was defined by a 25–50% transient reduction in functioning or requiring outpatient treatment. “Severe impairment” was defined by a 50% or more reduction in functioning, persistent impairment, or requiring inpatient treatment. Participants were asked to score (0 = none, 1 = mild, 2 = moderate, 3 = severe) for multiple domains of impairment (e.g., physical, cognitive, social, occupational), as well as indicate suicidality or inpatient hospitalization.

### Data processing

Interviews were digitally recorded, and audio files were transcribed by study personnel in the Clinical and Affective Neuroscience Lab at Brown University. Practitioner interviews were rendered verbatim, but vocalizations such as “um,” “like,” “kind of,” and “you know” as well as identifying information were removed. Names of teachers and Buddhist centers of study and practice were removed when this information could be used to identify the practitioner—for instance, if they were lineage holders in a tradition where the teacher had only a few direct disciples. Names of friends and family members, universities attended, and states of residence were also removed. Transcripts were then verified by a separate research assistant or study personnel to ensure accurate rendering of content. When necessary, religious studies scholars (JL, DC) were consulted to ensure accurate rendering of names of teachers, institutions, and places as well as tradition-specific foreign language terminology.

### Data analysis

Qualitative data analysis is a systematic means of organizing and then describing interview data. We used an exploratory, content-driven thematic analysis—where the analytical categories that are used to establish comparisons across reports derive primarily from the reports themselves—because this is the method that is recommended when there is little former knowledge about a phenomenon [101, 120]. Through this approach, we were able to establish systematic descriptions of reports of specific aspects of meditation experiences (phenomenology) as well as of specific interpretations, causal attributions, and remedies (influencing factors). The qualitative data analysis used open coding techniques [121], which are intended to “open up” the text in order to uncover its content and meaning. Following DeCuir-Gunby et al. (2011) [122], a “data-driven” approach was augmented by the incorporation of some “theory-driven” categories already existent in the literature on meditation, psychology, or phenomenology (e.g., [84, 123]). The coding structure was revised iteratively until saturation was reached—i.e., when new interviews produced little or no new data and no changes to the existing coding structure [101].

#### Codebook creation and revision

Reports of meditation experiences (phenomenology) as well as interpretations, causal factors, and remedies (influencing factors) were entered into computer-based qualitative analysis software (NVivo) for analysis. Following the methodology of open coding, and in particular the team-based approach summarized by Fonteyn et al. (2008) [124], coders assigned a tentative heading or category to each unit of analysis. They then read and coded the transcript until all relevant aspects of the content were categorized. The coding structure evolved from the initial open coding towards a defined codebook of recurring categories. Each category was given a standardized definition, inclusion and exclusion criteria, and an example excerpt [124, 125]. To define codebook categories in a way that was faithful to the reports of study participants, specific language or phrasing from interview transcripts was often incorporated into category descriptions, inclusion criteria, and exclusion criteria [101].

#### Phenomenology coding

Following a data-driven approach, interview content determined the codebook categories, which were organized into higher-order domains. To validate the phenomenology codebook, interviews illustrating a variety of categories were selected and coded independently by multiple researchers. Once a draft codebook had been established, the first 30 interviews were then recoded by pairs of researchers, and any disagreements led to iterative discussions that refined coding criteria until consensus was achieved. This draft codebook was then applied during the coding of the next 30 interview transcripts. In this phase, coders continued to track discrepancies within the coding structure, and interview content not represented by an existing category was coded as “other” according to its higher-order domain. After 60 interviews had been coded, all annotations and all content coded as “other” was reviewed, and the codebook was again revised. The addition, elimination, merging or relocation of categories, as well as discrepancies between previous codes and revised codes, was discussed until consensus among coders was reached. The final coding structure was then implemented by two coders (JL or DC) across all interview transcripts. A subset (15%) of practitioner interview transcripts were coded by both coders to ensure interrater reliability for the phenomenology codebook. Agreement ranged from 97% to 100% with an average agreement across categories of 99.5% (weighted kappa = .70).

#### Influencing factors (IFs) coding

Through coding phenomenology, it became apparent that there was little overall consensus on which meditation-related experiences are “beneficial,” “desired,” “positive,” or a “goal” of practice, and likewise there was little consensus on which experiences are “adverse,” “unwanted,” “negative,” or a “side-effect” of practice. Rather, whether a specific phenomenological category is reported as positive or negative—or as a goal or a side-effect—depends in part upon a complex process of appraisals and interpretive frameworks. For the purpose of better understanding how reports of experiences and various causes and interpretive frameworks interact in practitioner narratives, our qualitative analysis employed one coding structure for the reports of meditation-related experiences and a separate coding structure for interpretations, causal attributions, and responses or remedies.

Influencing factors (IFs) are comprised of the interpretive ascriptions concerning the significance and value of various meditation-related experiences, the causal attributions practitioners or experts made regarding the onset of meditation-related challenges, and the means for navigating or alleviating those challenges. The various causal factors that practitioners or experts believed impacted the presence or absence of certain meditation-related experiences were often presented in tandem with interpretive statements about the meaning, value, or valence of those experiences. Remedies refer to particular behaviors, approaches, activities, or contexts that they believed to be helpful in either preventing or alleviating meditation-related difficulties or the amount of distress and impairment associated with those experiences. Because many factors, such as relationships to teachers, were reported as risk factors by some practitioners and as remedies by others, risk factors and remedies were coded together under the framework of IFs.

Meditation practices are embedded both in broader systems of thought and in sociocultural contexts, and in some cases it was difficult to disambiguate attributions made exclusively to the techniques versus attributions made to the worldviews, value-systems, and social relationships associated with those techniques. The IFs codebook attempts to capture the various types of attributions practitioners and experts made in the process of attempting to explain the causal onset of meditation-related experiences reported as challenging, difficult, distressing, or functionally impairing.

Because expert interviews provided a second-person perspective on challenging meditation experiences they had seen and managed in their students or patients, they were a richer source of information for influencing factors than for phenomenology. Consequently, the IFs codebook was developed iteratively by coding expert transcripts. The methods for coding IFs and for validating the IFs codebook followed the procedure detailed above for phenomenology. To ensure interrater reliability, a team of three coders re-coded fifteen percent of expert interview transcripts (each transcript coded by two coders). Agreement ranged from 93% to 100% with an average agreement across categories of 97.5% (weighted kappa = .56).

## Results

### Participant flow

A total of 73 meditation practitioners completed interviews. Thirteen practitioner interviews were excluded from the current analysis for failure to meet inclusion criteria. Specifically, interviews with five practitioners revealed a mixed practice history that precluded clear classification as Theravāda, Zen, or Tibetan and/or included a significant influence from non-Buddhist meditation practices such as Hindu traditions or the use of technology-based meditation apps. Four practitioners were excluded from the current analysis for failing to report any challenging experiences. Four practitioners were excluded because their phenomenology could be accounted for by alternative causes. The remaining 60 practitioners were given the demographic and attributes form after all interviews were completed; 100% of respondents met causality assessment criteria (score>2, mean 4.2 ±1.1) and were included in the present analysis.

Fifty-three practitioners (88%) completed the follow-up questionnaire. For the 7 (12%) non-completers, information was extracted by researchers based upon the content of their interview transcript. Results are based on a sample size of 60 practitioners unless otherwise indicated (i.e., in the case that one or more participants declined to answer a survey item and/or did not volunteer the information during the interview).

### Sample characteristics

#### Practitioners

Participants were 60 Buddhist meditators (43% female, 57% male, mean age = 48.9 years, SD = 13.1, range 18–76) with equal representation (n = 20) across Theravāda, Zen, and Tibetan lineages. Given the study location and sampling strategy, most practitioners were from the United States (85%), with the remainder from Europe (8%), Canada (5%), or Mexico (2%). Participants were predominantly White (94.5%), with professional occupations, and had high levels of educational attainment. All but two had completed college, 25 (42%) had a Master’s degree, and 15 (25%) had an MD, a PhD or equivalent. Sixty percent of the practitioner sample identified as meditation teachers. Eleven of those practitioners provided subsequent expert interviews in which they described the experiences they had observed in their students, how they interpreted those experiences, and how they managed those students. While the sample represented a range of meditative expertise, nearly half of the sample (43%) had more than 10,000 lifetime hours of practice at the time of the interview (see Table 1).

**Table 1:** Practitioners’ demographic information.

**Age**	48.9 years (SD = 13.1) (range = 18–76)
**Gender**	43% female, 57% male
**Race/ethnicity**^a^	94% White, 2% Native American, 4% mixed/other; 5% Hispanic
**Education**	3% high school; 30% Bachelor’s degree; 42% Master’s degree; 25% Doctoral degree (MD, PhD, PsyD)
**Meditation teacher**	60%
**Lifetime meditation hours**	100–500 (3%); 500–1000 (5%); 1000–5000 (22%); 5000–10000 (27%); >10000 (43%)

See S3 File for data set.

^a^ n = 59.

#### Experts

In addition to the practitioner interviews, 32 experts were also interviewed (25% female, 75% male), 11 (34%) of whom previously provided practitioner interviews. Experts were 13 (40%) Theravādin, 8 (25%) Zen, 6 (19%) Tibetan, and 5 (16%) Clinical. In addition to the clinicians, four other meditation teachers also reported having clinical training (e.g., MA, MSW, PhD, PsyD or MD).

#### Types of meditation

Practitioners were engaged in a variety of Buddhist meditation practices (see Table 2). Some practices, such as placing attention on one’s breathing, were reported across traditions. Others, such as body scan, *kōan* practice, or visualization, were unique to particular traditions or lineages within those traditions.

**Table 2:** Types of meditation practices reported.

Type of practice	Dominant (% of sample)	Lifetime (% of sample)	At onset (% of sample)
concentration (*śamatha*, mindfulness of breathing, breath counting)	30 (50%)	57 (95%)	20 (33%)
insight (*vipassanā*, noting, open monitoring)	27 (45%)	48 (80%)	20 (33%)
body scan (including Goenka *vipassanā*)	4 (7%)	33 (55%)	5 (8%)
other insight practice (analytical meditation)	2 (3%)	25 (42%)	1 (2%)
*zazen*: breath counting	9 (15%)	28 (47%)	9 (15%)
*zazen*: “just sitting” (*shikantaza*)	12 (20%)	30 (50%)	8 (13%)
*kōan*	6 (10%)	15 (25%)	3 (5%)
loving kindness (*mettā*) or compassion	6 (10%)	49 (82%)	2 (3%)
*tonglen*	1 (2%)	28 (47%)	0 (0%)
nature of mind practice (*dzogchen*, *mahāmudrā*)	6 (10%)	28 (47%)	4 (7%)
Vajrayāna preliminary practices (*ngondro*)	5 (8%)	19 (32%)	8 (13%)
visualization practices	5 (8%)	20 (33%)	7 (12%)
mantra recitation	1 (2%)	25 (42%)	1 (2%)
other	3 (5%)	9 (15%)	8 (13%)

“Dominant” refers to the top two most frequently practiced types of meditation in the practitioner’s lifetime. “Lifetime” refers to the types of meditation reported as being practiced during the practitioner’s lifetime. Because most practitioners engaged in multiple types of meditation during the lifetime, the total percentage exceeds 100%. “At onset” refers to the top two most frequently practiced types of meditation around the onset of meditation-related challenges or difficulties. See S3 File for dataset.

### Context and practice at the onset of meditation-related challenges

The amount of meditation practice prior to the onset of challenging or difficult experiences ranged from 1 day to more than 25 years (mean = 7.1 years; SD = 8.0 years). More than a quarter (29%) of practitioners first encountered challenges within their first year of practice, almost one half (45%) between 1–10 years of practice, and one quarter (25%) after more than 10 years of practice. Seven participants (12%) reported challenges during the first 10 days of their practice, and 11 participants (18%) reported challenges during the first 50 hours of practice.

Challenges occurred during or immediately following a retreat for 43 practitioners (72%). The other 17 practitioners (28%) reported challenging experiences in the context of daily practice. About three-quarters (72%) of participants were regularly practicing within a meditation community or were working with a teacher (75%) when challenging experiences arose. At the time of meditation-related challenges, 14 practitioners (25%) were practicing meditation for 30–60 minutes per day, 19 (34%) were practicing for 1–9 hours per day, and 23 (41%) were practicing for 10 or more hours per day (see Table 3).

**Table 3:** Practitioner characteristics at onset of meditation-related challenges.

**Age at onset**	35.6 (SD = 11.8) (range = 17–63)
**Psychiatric history**^a^	18 (32%)
**Trauma history**^b^	25 (43%)
**Prior practice amount**^c^	7.1 years (SD = 8.0), range = 1 day- 25 years days (12%); months (17%); 1–10 years (45%); >10 years (25%)
**Practice context**	43 (72%) during retreat; 17 (28%) during daily practice
**Practice amount at onset**^d^	14 (25%) less than 1 hour/day; 19 (34%) 1–9 hours/day; 23 (41%) 10 more or hours/day

See S3 File for dataset.

^a^ n = 57

^b^ n = 58

^c^ n = 59

^d^ n = 56.

### Phenomenology: Domains and categories

Thematic content analysis of practitioner interviews for phenomenology captured the various types of experiences that practitioners associated with meditation. Fifty-nine categories of meditation-related effects were clustered into 7 higher-order domains (see Table 4). All practitioners reported meditation-related changes across multiple domains. The average number of domains reported per practitioner was 6.1 (SD = 1.00), with a range from 3–7. Nearly three-quarters (73%) of practitioners reported 6 or more domains, with coverage of all 7 domains as the most common pattern (43%). Each domain was reported by at least three-quarters of the sample.

**Table 4:** Phenomenology coding structure.

Cognitive	Perceptual	Affective	Somatic	Conative	Sense of Self	Social
10 categories 93% reported	7 categories 78% reported	13 categories 100% reported	15 categories 88% reported	3 categories 82% reported	6 categories 75% reported	5 categories 90% reported
Change in worldview (48%)	Hallucinations, visions, or illusions (42%)	Fear, anxiety, panic or paranoia (82%)	Somatic energy (63%)	Changes in motivation or goal (78%)	Changes in self-other or self-world boundaries (53%)	Social impairment (50%)
Delusional, irrational, or paranormal beliefs (47%)	Visual lights (33%)	Positive affect (75%)	Sleep changes (62%)	Change in effort or striving (42%)	Loss of sense of agency (25%)	Integration following retreat or intensive practice (47%)
Mental stillness (37%)	Somatosensory changes (32%)	Depression,dysphoria, or grief (57%)	Pain (47%)	Anhedonia and avolition (18%)	Loss of sense of basic self (25%)	Change in relationship to meditation community (45%)
Vivid imagery (35%)	Perceptual hypersensitivity (28%)	Re-experiencing of traumatic memories (43%)	Pressure, tension or release of pressure, tension (38%)		Change in sense of embodiment (22%)	Occupational impairment (42%)
Change in executive functioning (33%)	Distortions in time or space (25%)	Change in doubt, faith, trust or commitment (40%)	Appetitive or weight changes (38%)		Change in narrative self (22%)	Increased sociality (7%)
Meta-cognition (30%)	Dissolution of objects (18%)	Crying or laughing (38%)	Thermal changes (37%)		Loss of sense of ownership (18%)	
Increased cognitive processing (25%)	Derealization (7%)	Empathic or affiliative changes (32%)	Involuntary movements (37%)			
Clarity (20%)		Rage, anger, or aggression (30%)	Breathing changes (27%)			
Disintegration of conceptual meaning structures (12%)		Affective lability (28%)	Parasomnias (27%)			
Scrupulosity (3%)		Self-conscious emotions (25%)	Headaches or head pressure (22%)			
		Agitation or irritability (23%)	Cardiac changes (20%)			
		Suicidality (18%)	Fatigue or weakness (20%)			
		Affective flattening or emotional detachment (17%)	Gastrointestinal distress or nausea (17%)			
			Dizziness or syncope (15%)			
			Sexuality-related changes (15%)			

The number of categories in each domain varied, ranging from 3–15. The average number of total categories reported per practitioner was 19.6 (SD = 7.2), with a range from 7–40. Half of the sample (50%) reported more than 20 categories, with 10% reporting more than 30, and 5% reporting less than 10. Each category was reported by an average of 20 different participants (SD = 10.2), with a range from 2 (for scrupulosity) to 49 (for fear), indicating both maximal consistency and range.

Table 4 displays the seven domains horizontally from left to right. The categories of each domain are listed vertically in descending order of frequency (percent of practitioners reporting).

As a group, the 32 experts reported 56 out of 59 (95%) phenomenology categories, with an average of 8.5 categories (SD = 5.6) per expert. The three categories that were reported by practitioners but not experts were: *Crying*, *laughing*; *Distortions in time or space*; and *Fatigue*, *weakness*. While a detailed comparison between meditators' and experts' reports of meditation-related experiences is beyond the scope of this paper, it is worth noting that three of the categories most frequently reported by practitioners—*Fear*, *anxiety*, *panic*, *or paranoia* (82%), *Positive affect* (75%), *and Somatic energy* (63%) were also among the most commonly reported by experts (72%, 50%, and 41% respectively). *Re-experiencing of traumatic memories* also had similar frequencies for experts (47%) and practitioners (43%). These similarities between practitioner and experts reports were not impacted by the 11 participants who provided both practitioner and expert interviews. Experts’ data remained unchanged regardless of whether those 11 individuals were included or excluded from analysis. However, as explained above, because expert interviews provided second-person reports about their students, their accounts were less informative for phenomenology coding than practitioner interviews. They provided fewer reports and those reports were characterized by less precise descriptions such as “emotional problems” or “mental instability.”

### Description of phenomenology domains

The following summary is intended to provide some basic descriptions of categories beyond the category titles in Table 4. As described above, phenomenology categories were largely data-driven—that is, based upon the language and concepts used by practitioners in reporting their experiences during the interview. However, in later iterations of the codebook, it was important to refine category descriptions and distinctions in order to ensure that categories could encompass reports from multiple individuals as well as the language and perspectives from appraisals both within and beyond Buddhist traditions. To further refine distinctions, for some categories this data-driven approach was augmented by a theory-driven approach by drawing upon etic concepts from psychology, neuroscience, phenomenology, and religious studies. Given the number of categories, the number of references for each category, and the complex relationship among categories, a complete and detailed exploration of each category is beyond the scope of this paper. The summaries of each domain provided below will be unpacked in much greater depth in subsequent publications and should therefore be regarded as preliminary. For a comprehensive description of each category, including brief descriptions, inclusion criteria, and exclusion criteria, see S4 File.

#### Cognitive

Changes reported in this domain pertain to mental functioning, including the frequency, quality and content of thoughts, as well as other cognitive processes, such as planning, decision-making and memory. Three cognitive changes associated with concentration—*mental stillness* (periods of few or no thoughts), *clarity* (whether of cognitive processing or of awareness more generally), and *meta-cognition* (monitoring of cognitive processes)—were given various positive and negative associations depending upon their intensity and how they intersected with other changes in perceptual, somatic, affective, or sense of self domains. *Scrupulosity*, or obsessive and repetitive thoughts about ethical behavior, was primarily a concern for practitioners in a monastic context where adherence to ethical norms and regulations was considered integral to meditation practice. *Changes in worldview* pertained to shifts in ways of thinking about the nature of self or reality, including confusion about such views. The principal impairments in the cognitive domain were problems with *executive functioning* (inability to concentrate for extended periods, or problems with memory) and the *disintegration of conceptual meaning structures*, where percepts and concepts became disconnected. *Increased cognitive processing* speed, colloquially described as “mind racing,” also tended to be reported as unpleasant, and *vivid imagery* was given positive or negative valence depending on the content or intensity.

The category that the research team had the greatest difficulty operationalizing was *delusional*, *irrational*, *or paranormal beliefs*, in part because a particular belief could be appraised in multiple ways depending on the practitioner and his or her social context. In addition to beliefs described by the practitioner in retrospect as delusional or irrational in nature (e.g., disconfirmed by objective evidence), this category also included beliefs that seemed unusual or concerning either to an authority in their culture or subculture, such as a meditation teacher, or to a family member. When transient, delusional beliefs tended to have little impact; however, when enduring and coupled with a loss of reality testing, delusional beliefs had a much greater impact and tended to lead to functional impairment and changes in the social domain.

#### Perceptual

The perceptual domain captures changes to any of the five senses: vision, hearing, smell, taste and somatosensory processing (including interoception and proprioception). One common change in this domain was *hypersensitivity* to light, sound, or sensation. Visual hypersensitivity often began with increased color vividness (hyperchromia). Related phenomena included a general brightening of the visual field, which was sometimes associated with simple hallucinations in the form of *visual lights*; these two phenomena have been discussed in detail in an analysis of preliminary data from this study [126]. Perceptual hypersensitivity was also commonly associated with increased cognitive processing, and tended to be reported as distressing during transitions from intensive practice into daily life. Within practice traditions where concentration on the fleeting nature of percepts is a common approach, practitioners reported the *dissolution of perceptual objects*; in some cases, the cessation of all visual perception was reported. Other perceptual distortions included *distortions in time and space*, and *derealization*—where phenomena appear dreamlike, unreal, two-dimensional or as if in a fog [127].

*Illusions* (distortions of perceptual objects) *and hallucinations* (a percept-like experience in the absence of a sensory stimulus) [128] were reported both in isolation from and in conjunction with delusional beliefs. Some practitioners also reported phenomena that were technically hallucinations—in the sense that they were percepts in the absence of an external stimulus—but were interpreted as visions and attributed to an external agent or force. Unlike some other hallucinations, visions tended to be transient and did not appear outside of a formal meditation session. *Somatosensory changes* included changes in body schema, whether heightened interoception or distorted perceptions of body parts, which could also take on a quality similar to illusions.

#### Affective

Affective changes refer to changes in the type, frequency, or intensity of emotions. A wide range of emotional changes was reported, capturing both discrete (primary) experiences and also responses to changes in other domains (secondary). Practitioners reported having both increased as well as decreased emotionality. In terms of increased emotionality, *fear*, *anxiety*, *panic or paranoia* was the most frequently reported category, not only in the affective domain, but across all domains, with 82% of practitioners reporting it. Often the fear or anxiety was an additional response of negative affect that coincided with other unexpected or undesired changes, but in some cases fear was non-referential and was reported as a phenomenological change unto itself. Increased emotionality also took the form of increased *affective lability*, sensitivity, or reactivity in response either to people or to other environmental stimuli. Emotional sensitivity to others often manifested as *empathic and affiliative changes* (increased feelings of empathy, or sharing others’ emotions) between the practitioner and other human beings. In contrast to increased emotionality, some practitioners reported having fewer or less intense emotions or *affective flattening*, sometimes even the complete absence of emotions.

*Positive affect*, including bliss and euphoria were also commonly reported, but sometimes were followed by subsequent depression or agitation, either within the context of a practice or in the transition from formal practice to daily life. In some cases, *depression* became sufficiently severe to result in *suicidal ideation*. In other cases, intense positive affect did not alternate with low arousal states, but instead escalated into destabilizing conditions resembling mania and psychosis, which often required hospitalization. It should be noted that neither “mania” nor “psychosis” were phenomenological categories in our coding structure, even if practitioners, or more commonly, experts used such terms to describe an experience. Rather, because our categories aimed to capture distinguishable components of experience, what practitioners referred to as “mania” was found to be typically composed of a combination of positive affect and increased processing speed, and in some cases, delusions.

*Changes in doubt and faith* as well as *self-conscious emotions* (guilt, shame, pride, etc.) were often secondary responses to other meditation experiences and typically had an impact in the social domain as well. Although typically a secondary response, shame in particular was a large contributor to levels of distress. For practitioners with a trauma history, it was not uncommon for them to report a *re-experiencing of traumatic memories*, and even practitioners without a trauma history similarly reported an upwelling of emotionally-charged psychological material. Practitioners reported involuntary *crying or laughter* in response to positive affective content such as bliss or joy, in response to negative affective content like grief or sadness, or in some cases without content altogether. Other states of negative affect included *increased agitation or irritability*, which could become intensified to either transient outbursts or long-term expressions of *anger and aggression*.

#### Somatic

The somatic domain included observable changes in bodily functioning or physiological processes. The study documented a large number of physiological changes, many of which were infrequently reported across subjects. *Dizziness or syncope*, *gastrointestinal distress*, *cardiac irregularity*, *breathing irregularity*, *fatigue*, *headaches* and *sexuality-related changes* were all reported by fewer than 20 participants. A more commonly reported physiological effect was *changes in sleep* need, amount, or quality, with practitioners tending to report loss of sleep need, decreased sleep amount, or insomnia (see also [129]). Other sleep-related changes included *parasomnias* such as nightmares and vivid or lucid dreams. Sleep-related changes frequently co-occurred with *appetitive changes*, especially a decrease in appetite or food intake. *Thermal changes* included both feeling warmer and colder throughout the body, and more localized sensations of heat and cold.

One principal set of changes in the somatic category included reports of *pressure and tension* in the body, or sometimes intense *pain*, which would become more acute or release in the course of contemplative practice. The release of pressure or tension was sometimes associated with positive affect and surges in energy; however, it was also associated with the re-experiencing of traumatic memories and other forms of negative affect. In some cases, the release of tension was associated with reports of electricity-like “voltage” or “currents” of *somatic energy* surging through the body. Somatic energy could be under practitioners’ control or beyond their control. This was the most commonly reported experience in the somatic domain and was associated with a wide range of other somatic changes as well as changes in other domains. For instance, when surges in somatic energy were particularly strong, *involuntary body movements* sometimes followed.

#### Conative

The conative domain primarily denotes *changes in motivation* or goal-directed behaviors. This change frequently co-occurred with changes in worldview and changes in the social domain. Another conative change was the reported amount of *effort or striving* associated with meditation practice. On the one hand, practices that previously required great effort sometimes became effortless, a change generally reported as a positive. On the other hand, increased levels of effort or “striving” were also described as corresponding with increased arousal with corresponding affective, perceptual, and somatic changes that could be associated with unpleasant or destabilizing conditions. The two phenomena practitioners reported as impairing in the conative domain were the lack of desire for activities one previously enjoyed (*anhedonia*) and the loss of motivation to pursue goals (*avolition*). These often co-occurred with other functional impairments, such as changes in social or occupational behaviors. When conative phenomena were described less as changes in and of themselves and more as causal factors for the onset or alleviation of difficulties, they were coded as influencing factors (see Influencing factors: Domains and categories).

#### Sense of self

Given that the sense of self is construed in multiple ways—from fundamental embodied sensorimotor activity to more complex conceptual judgments—various changes in sense of self were differentiated according to data-driven reports and theory-driven perspectives from phenomenology and cognitive science (e.g.,[130]). *Changes in the narrative self* refer to shifts in how a practitioner conceives of himself or herself over time, often in relation to the identities, worldviews, values, goals or behaviors both within and beyond their Buddhist tradition. Other changes in sense of self occurred at more fundamental levels that had a corresponding impact on cognitive, affective, somatic, or perceptual domains. For instance, *changes in sense of embodiment* referred to feeling displaced from one’s ordinary location relative to one’s body schema, and detailed descriptions of this phenomena highlighted corresponding affective and perceptual changes in particular.

The most common change in sense of self reported by practitioners was a *change in self-other or self-world boundaries*, which took many related forms. Some practitioners reported boundaries dissolving and general permeability with the environment or with other people; others felt like their self had expanded out from their body and merged with the world; still others used the inverse language, reporting that the world had become merged with their sense of self. A range of different affective responses were associated with this change, from neutral curiosity, to bliss and joy, to fear and terror. *Loss of sense of ownership* was commonly reported in relation to thoughts, emotions, and body sensations. Practitioners also reported a *loss of sense of agency*—or the loss of a “doer” of actions—in relation to automatic actions such as crying, to habitual actions such as walking, and to typically intentional actions such as speaking. Some practitioners reported even more fundamental changes in their sense of self akin to a *loss of the sense of basic self* [131] or the minimal self [132] such that they felt like they no longer existed at all or that they would disappear or be invisible to others.

#### Social

The social domain includes any changes in interpersonal activities or functioning, including level of engagement, quality of relationships, or periods of conflict, isolation or withdrawal. The social domain tends to involve either experiences that catalyzed meditation difficulties or, conversely, were the consequence of meditation difficulties. Social factors were described as catalysts for difficulties in *integration following retreat or intensive practice* where transitioning from a practice context (whether in daily life or on retreat) to a non-practice (and often social) context were experienced as destabilizing. For example, perceptual, affective, and cognitive changes that were not problems in the practice context became difficulties that were reported as negatively valenced or impairing of functioning at work or with family. *Social impairment* includes both these instances as well as instances where practice-related difficulties continued into daily life. This domain also includes *changes in occupational functioning*, which often requires social interactions. A generally positive but less commonly reported change was an *increased sociality*, defined as an increased extraversion or valuing of social connections. *Changes in relationship to meditation community* (including both teachers and other practitioners) included feelings of support and encouragement as well as feelings of estrangement or rejection, often co-occurring with changes in worldview and changes in doubt or faith, especially when challenging meditation experiences resulted in significant distress or functional impairment. Other aspects of social relationships described as contributing to the onset or resolution of challenging meditation experiences were coded as Influencing Factors under the *Relationship* domain (see Influencing factors: Domains and categories).

### Causality criteria

Causal attribution to meditation was assessed with 11 criteria (see above Additional instruments and quantitative measures: Causality assessment). *Prior published reports* included more than 40 published reports, including case reports or studies [64, 65, 67–73, 77–79, 82, 84–86, 133–146], reviews of meditation-related risks, adverse effects or contraindications, [63, 147–154], medical textbooks [74–76, 155] and MBI implementation guidelines [62]. *Expert judgment* was derived from interviews with 32 meditation teachers and clinicians who reported 56 categories of meditation-related experiences that they had observed in their students.

Through the causality assessment section of the demographic and attributes follow-up questionnaire, practitioners reported on six causality criteria: *subjective attribution*, *temporal proximity (challenge)*, *exacerbation*, *consistency*, *de-challenge*, and *re-challenge*. Meditation practitioners met an average of four causality criteria (mean = 4.2 ±1.1 range = 2–6;), with more than half (60%) meeting four, five, or all six criteria, well-exceeding the minimum cutoff of two that standard guidelines use to warrant further investigation [118]. Table 5 shows the percent of the sample meeting each individual criterion.

**Table 5:** Causality criteria.

Causality criterion	% of sample
subjective attribution	93
temporal proximity (challenge)	85
exacerbation of previous symptoms	58
consistency (multiple occasions)	88
de-challenge	60
re-challenge	61

The *consistency* criterion was assessed on three levels: In the current study, 88% of the practitioner sample met the *intra-subjective consistency* criterion, indicating that they reported having the same or a similar experience during or following meditation on multiple occasions.

*Inter-subjective consistency* was assessed through evaluating how many practitioners reported each category (see Table 4 above). Each category of experience was reported by an average of 20 practitioners, indicating that the same or a similar experience occurring in temporal proximity to meditation was reported by multiple individuals. *Cross-modal consistency* was assessed by comparing the phenomenology reported in practitioner interviews to the phenomenology reported in expert interviews. Fifty-six of 59 (95%) categories of experience were reported by both practitioners and experts, with an average of 20 (SD = 10.2) practitioners and 4.6 (SD = 4.3) experts reporting each category. As reported above, similarities between practitioner and expert reports were not impacted by the 11 participants who provided both practitioner and expert interviews.

### Duration, severity and associated impairment

**Fig 1:**
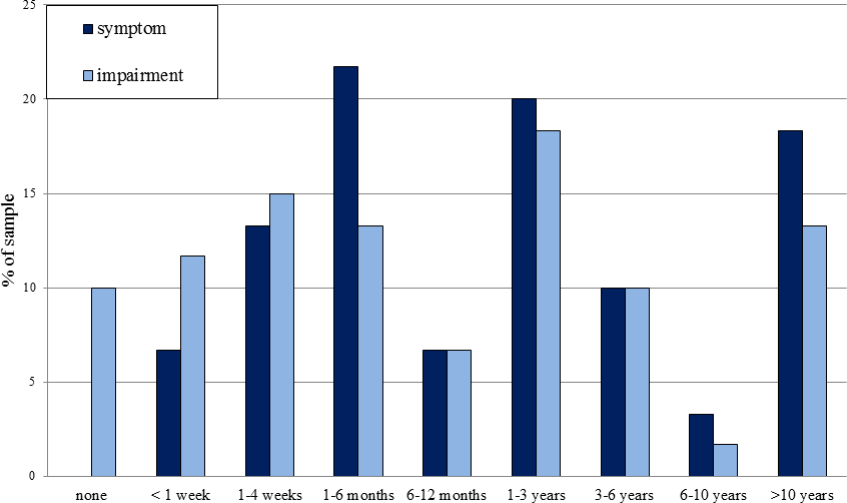
Durations of symptoms and impairment.

The vast majority (88%) of participants reported that challenging or difficult meditation experiences bled over into daily life or had an impact on their life beyond a meditation retreat or beyond a formal practice session. The term “symptoms” is used here to denote the subset of experiences that were experienced as challenging, difficult or functionally impairing. Fig 1 shows the duration of symptoms and their associated impairment. The median duration of symptoms was 1–3 years, ranging from a few days to more than 10 years. While 10% reported minimal impairment, impairment tended to mirror symptom duration, lasting from days to more than a decade. The majority of the sample (73%) indicated moderate to severe impairment in at least one domain, with 17% reporting suicidality, and 17% requiring inpatient hospitalization.

### Influencing factors: Domains and categories

Thematic content analysis of both practitioner and expert interviews for influencing factors (IF) captured the contextual factors that practitioners or experts associated with the onset of meditation-related experiences and the way those experiences changed over time. Twenty-six categories of influencing factors were clustered into 4 higher-order domains.

The average number of influencing factors reported per person was 15.1 (SD = 3.5, range 9–23) for practitioners, and 14.7 (SD = 3.3, range 8–21) for experts. Each category was reported by an average of 35 different practitioners and 19 different experts, indicating high consistency across both practitioners and experts.

Table 6 displays the 4 domains of IFs horizontally from left to right. The categories of each IF domain are listed vertically in descending order of frequency (percent of practitioners reporting) [percent of experts reporting].

**Table 6:** Influencing factors coding structure.

Practitioner	Practice	Relationships	Health Behaviors
7 categories	5 categories	6 categories	8 categories
Worldview or explanatory frameworks (97%) [85%]	Amount, intensity, or consistency of practice (93%) [94%]	Relationship to teacher (97%)[97%]	Psychotherapy or medical treatment (68%)[79%]
Intentions, motivations, or goals (87%)[88%]	Practice approach (93%)[94%]	Relationships beyond practice community (85%)[67%]	Diet (46%)[24%]
Personality or temperament (70%)[88%]	Type of practice (87%)[84%]	Relationships within practice community (83%)[67%]	Medication (40%)[58%]
Psychiatric history (43%)[88%]	Response to experience (77%)[54%]	Surroundings or environment (70%)[55%]	Grounding activity (38%)[55%]
Trauma history (38%)[54%]	Stage of practice (43%)[79%]	Sociocultural context (45%)[70%]	Sleep (38%)[18%]
Medical history (23%)[9%]		Early life relationships (35%)[21%]	Recreational drugs (35%)[18%]
Identities (22%)[21%]			Exercise (33%)[21%]
			Bodywork or Energy Healing (25%)[6%]

### Description of influencing factor domains

The following summary aims to clarify how influencing factors were defined and operationalized, which was primarily data-driven from practitioner and expert interviews. This enumeration of influencing factors should not be taken as a theory or hypothesis about risk factors and remedies for meditation-related difficulties put forth by the authors; rather, it reflects the views and experiences of the practitioners and experts who were subjects in our study. Further analysis, as well as research in controlled conditions, is necessary in order to evaluate whether these influencing factors are in fact correlated with a category of experience, the duration of challenging or difficult experiences, or the associated degree of distress or impairment. For a comprehensive description of each category, including descriptions, inclusion criteria, and exclusion criteria, see S5 File.

#### Practitioner IFs

This domain captures reported influencing factors occurring at the practitioner level. The *identities* category refers to demographic variables such as gender, age, ethnicity, religion, and so forth when these were reported as having an impact on a practitioner’s meditation experience or degree of social support received by a meditation community. *Medical history*, *psychological history*, and *trauma history* were reported as having an impact on the presence of particular meditation experiences in somatic, cognitive, and affective domains, respectively, as well as on the duration of meditation-related difficulties. The interaction between meditation and pre-existing psychiatric or trauma history was a common interpretation and causal attribution put forth by experts for certain meditation-related challenges. *Personality* characteristics and temperaments were identified as being potentially either a risk factor or a remedy, depending on the particular characteristics mentioned. Experts often explained certain difficulties as due to the way meditation practices are thought to affect personality structures. Similarly, certain *intentions*, *motivations*, *or goals*, as well as certain *worldviews or explanatory frameworks* were characterized as helpful and supportive of contemplative development, whereas others were attributed as being harmful and as intersecting with other risk factors in the practice domain. Worldviews and explanatory frameworks were also influencing factors in that certain interpretations of the meaning of meditation-related difficulties could lead to further difficulties or the alleviation of certain dimensions of difficulties. Practitioners who reported holding or being offered multiple, conflicting worldviews were particularly likely to report on the influential—and often confusing—role of interpretive frameworks.

#### Practice IFs

Practice-level influencing factors pertain to how a practitioner engaged in a particular contemplative practice. The *amount*, *intensity*, *or consistency of practice* was identified as a risk factor when intensive periods converged with certain practitioner-level influencing factors such as personality or other practice-level factors such as type of practice; however, experts in particular also promoted consistency of practice as a remedy for meditation difficulties expected to be transient in nature. *Practice approach* referred to incorrect ways of practicing meditation that were characterized as risk factors as well as the use of correct methods posited as remedies. A related category called *response to experience* referred to how practitioners either did or should respond to meditation-related changes that endure beyond the formal practice session. *Type of practice* could be identified as a risk factor for particular meditation experiences simply due to the nature of the practice, or due to a mismatch between type of practice and practitioner dispositions. Changing type of practice or complementing one practice with another was offered as a potential remedy for the latter type of difficulties. Certain difficulties were interpreted, especially by experts, as a necessary *stage of practice*, only to be resolved by passing through the stage signified by specific meditation-related experiences or by integrating stage-related changes into one’s experience. An assumption articulated in this context was that subsequent stages of practice could or would resolve challenging or difficult meditation-related experiences.

#### Relationship IFs

Relationships pervade the lives and contexts of practitioners, ranging from the impact of their early life family relationships, to the quality of relationships with meditation teachers and communities, to the amount of social support outside of the context of meditation. Some *early life relationships* were associated with practitioner-level psychiatric or trauma history; conversely, supportive early life relationships also were described as having an impact on personality traits. *Relationships within meditation communities* and especially *relationships to teachers* were reported as being both risk factors for difficulties when teachers and communities were absent, unhelpful, or not sympathetic, as well as being remedies if teachers and communities were supportive, helpful and understanding. Experts also often commented on the importance of healthy dynamics in the student-teacher relationship for the student’s negotiation of meditation-related difficulties. *Relationships beyond the meditation community* also had a range of impacts—from risk factor to remedy—depending on whether or not those relationships were stable or supportive. The practice *surroundings or environment*, especially in a retreat context of silence and social isolation, was commonly described as a risk factor, such that changes in environment were necessary for alleviating certain challenges that emerged during retreat. So too, changes from a retreat context to certain destabilizing or challenging environments were also reported as risk factors for social and occupational difficulties in particular. Another prevalent theme was practitioners’ perceived degree of compatibility between the worldviews, values, and goals shaped by meditation experiences and meditation teachings and the broader *sociocultural context* they inhabited. When there was compatibility and fit, sociocultural contexts could be part of a remedy. However, experts and practitioners alike also suggested that certain sociocultural contexts could be risk factors, particularly when there was a mismatch between a practitioner’s and a teacher’s cultural background and social customs. Another interpretation offered was that mismatches between practitioners’ meditation experiences and worldviews and values of their sociocultural contexts could create a tension that would lead to or compounded difficulties.

#### Health behavior IFs

Health behaviors were generally categorized as potential risk factors when absent or out of balance and as remedies when present or in balance. For example, lack of *sleep*, inadequate *diet*, and lack of *exercise* tended to be associated with (or preceded) destabilizing experiences, and could be corrected as remedies by increasing sleep amount, making dietary changes, or getting exercise, as well as by engaging in other activities described as *grounding*, calming, or embodying. *Recreational drugs* were sometimes cited as risk factors for certain experiences, although prior drug-related experiences were also reported as a helpful foundation to have for negotiating certain types of destabilizing meditation-related experiences. Drug use was also occasionally reported as an attempt to alleviate meditation difficulties, with mixed results. More commonly cited as helpful was a regimen of *medication*, especially for severe meditation-related difficulties requiring other intensive treatments and hospitalization. Other remedies included *psychotherapy or medical treatment*, and experts in particular interpreted certain meditation difficulties as requiring the temporary suspension of meditation practice in order to address them psychotherapeutically. For certain symptoms in the somatic domain, *body-based healing* regimens (such as massage, acupuncture, or healing techniques that manipulate the subtle “energy” of the body) were also attempted and reported as helpful by some but not others.

As with phenomenology, the degree to which certain influencing factors or remedies were appraised as helpful or harmful was highly variable and case specific. While some remedies were enthusiastically endorsed, many of the remedies that were attempted or prescribed by others were described by practitioners as ineffective or harmful.

## Discussion

Based upon qualitative interviews of Western Buddhist practitioners and experts, the Varieties of Contemplative Experience study developed a taxonomy of meditation-related experiences, with a special effort to capture under-reported experiences characterized as challenging, difficult, distressing, or impairing, and which may need specific kinds of support. Thematic content analysis yielded 59 categories of experiences across 7 domains, including cognitive, perceptual, affective, somatic, conative, sense of self, and social. Each category was reported by an average of 20 practitioners and 5 experts, indicating high consistency across participants. The valence and level of impact ranged from very positive to very negative, with the associated level of distress and functional impairment ranging from minimal and transient to severe and lasting. This range of experiences and impacts suggests that very few of the experiences were universally appraised as adverse. Instead, the valence and impact of any category of experience was dependent on a complex interaction with other influencing factors. Thus, in order to determine what variables affect the valence and impact of a given experience, the study also identified 26 categories of influencing factors across 4 domains, including factors related to the practitioner, the practice, relationships, and health behaviors.

### Appraisal processes and differential diagnosis

While one of the central aims of the VCE study is to understand the influence of meditation techniques on resultant experience, querying phenomenology within the broader context of influencing factors—some of which are sociocultural in nature—enhances our understanding of the impact of cultural contexts and conceptual frameworks on meditation experiences, as well as on how such experiences are appraised. Assessing the role of appraisal was built directly into the VCE study interview protocol by asking practitioners and experts to differentiate experiences associated with meditation from how they are interpreted, how they are believed to be caused, and how they are to be addressed. While querying descriptive reports of experience independently from interpretations and causal attributions is not intended to “separate an unmediated experience from a culturally determined description”[50], this approach nevertheless facilitates a better understanding of how causal attributions and ascriptions of significance, meaning, and value are applied to experiences. It also identifies the influence that social agents have on the appraisal of meditation-related experiences, ranging from meditation community members and especially meditation teachers to agents beyond meditation communities such as doctors and psychotherapists.

The current study not only highlights the central role of appraisal in interpreting meditation experiences, but also that there are multiple, and sometimes conflicting, interpretative frameworks at play for Western Buddhist meditators. The values held by meditation practitioners are often heavily influenced by how the authorities of a Buddhist tradition—whether canonical texts, teachers, or members of a practice community—appraise a given meditation practice or experience. Prior conceptual frameworks acquired through philosophical or theoretical study, communities, families, as well as the media, likely influence how those who encounter meditation-related difficulties appraise such experiences. While explanatory frameworks for meditation-related experiences exist within Buddhism, what is categorized as “progress” versus “pathology” may differ across traditions, lineages, or even teachers. Furthermore, these models may only be immediately available to practitioners working closely with a teacher or Buddhist community. In addition, even when traditional frameworks are available, these models may be inadequate for Western meditators who seek meditation for therapeutic reasons and who are embedded in a scientifically-oriented culture, where biomedical and psychological frameworks have a pervasive influence. Wildman (2011) [156] suggests that “the intense experiences of non-religious people are sometimes difficult to assimilate for the lack of any conceptual framework or social context for making sense of them,” a point that may extend to practitioners unfamiliar with or uninterested in traditional conceptual frameworks or to those holding multiple, incompatible frameworks. That is to say, it may be the case that *some* of the “adverse” responses to meditation experiences can be attributed to a lack of fit between practitioner goals and expectations and the normative frameworks of self-transformation found within the tradition. Thus, Western Buddhist practitioners not only have to navigate multiple interpretative frameworks, but also different opinions about which frameworks have authority.

Similarly, recent scholarship on religion and mental health has attempted to establish comparisons—especially differences—between “religious experiences” and experiences associated with psychopathology, particularly schizophrenia and psychosis [87–90]. While these studies grapple with important issues of differential diagnosis, they tend to place an excessive emphasis on the role of “belief” in religious life, while overlooking the various changes in perception, affect, cognition, embodiment, and sense of self that are also reported in the context of both religious experiences and mental illnesses. Many of the experiences reported by practitioners in our study resemble to varying degrees phenomena discussed in the vast literature on schizophrenia, schizotypy, psychosis, as well as non-psychopathological forms of anomalous experience. Without sufficiently attending to the role of appraisal processes at both individual and interpersonal levels, scholars may fundamentally misconstrue differential diagnosis as being about identifying inherent differences between religious experiences and mental illnesses, rather than seeing them as potentially more ambiguous categories or closely related phenomena that may well be grounded in common cognitive, perceptual, and behavioral mechanisms. Furthermore, an emphasis on intrinsic differences between religious experiences and psychopathology obscures the social and cultural processes through which experiences are deemed either religious or pathological [94]. Many experiences reported in the VCE study are not unique to religious traditions and are, therefore, not intrinsically religious [157, 158]. Through querying attributions of causality and ascriptions of meaning and value independently from phenomenology, we attempt to situate reports of experiences in the broader narrative and cultural contexts of practitioners and their communities.

### Relevance to meditation-based programs and centers

The current study did not directly investigate meditation-related difficulties in mindfulness-based interventions (MBIs). Although some practitioners in our study engaged in types of meditation practices (e.g., visualization), in contexts (e.g., long term retreat), and with motivations (e.g., attainment of religious goals) quite different from MBIs, a number of participants also reported challenging or difficult experiences under similar conditions as MBIs, that is: in the context of daily practice; while meditating less than 1 hour per day, or within the first 50 hours of practice; and with an aim of health, well-being or stress-reduction. Some types of practice associated with challenging meditation experiences were in many cases not dissimilar from the primary components of MBIs. Many practitioners in our study reported difficulties associated with concentration practices (focused attention-type practices such as mindfulness of breathing), or insight practices (open monitoring-type practices), or a combination thereof [25, 159]. Furthermore, MBI participants are encouraged to continue practicing after the 8-week program and are recommended to attend retreats at Buddhist meditation centers [160]. However, little is known about participant trajectories beyond the 8-week program, as most follow-ups are less than 1 year and are rarely more than 3 years [161, 162]. Given that both MBIs and Buddhist meditation centers are based on Buddhist theory and practice [24, 25], and given that these same sources describe the possibility of periods of challenge or difficulty associated with practice, it makes sense that MBI providers and meditation teachers would be familiar with these challenges and know how to manage them.

The current study provides meditation teachers with a detailed taxonomy of potentially challenging experiences that could assist in the identification of students who may need additional support or corrective instruction. Such early identification has been shown to improve general and mitigate negative outcomes [163]. The study also provides a preliminary list of remedies and management strategies, suggested by both experts and practitioners, as well as potential influencing factors that should be further investigated in both research and practice. For example, research is beginning to identify practitioner-level factors, such as trauma history [61, 164] or psychiatric conditions [165], that may influence meditation-related outcomes or require modifications in teacher training, program structure, or inclusion and exclusion criteria [166].

### Limitations

The purpose of this study is to document experiences that arise in the context of Buddhist meditation practices in Western contexts, with a deliberate effort to investigate challenging, difficult, distressing, and functionally impairing experiences that are often under-reported. As such, using a qualitative methodology with a sample of Western Buddhist meditators across traditions is the best approach to be able to document the range of under-reported experiences. While suitable for the purposes of the current study, the methods also preclude certain types of conclusions, which are outlined below.

#### Non-representative sample

The study deliberately sampled meditators who had challenging meditation experiences that are often under-reported. Thus the 100% frequency of challenging experiences is an artifact of sampling and not a reflection of the actual frequency among Western Buddhist meditators. While valuable for gathering data on under-researched phenomena, purposive sampling does not grant the ability to generalize any findings of the study to other samples such as Asian Buddhist meditators or Western meditators in general. It should be noted that the results of this study also cannot be generalized to children or youth under the age of 18, or to mindfulness-based interventions (MBIs), neither of which were included in our sample. While our sampling was largely representative of a certain demographic of Western Buddhist meditators [167], this is only one way of characterizing practitioners of Buddhism in the West [168, 169]. In particular, since our sampling did not yield a representative number of Asian-American practitioners, study findings cannot be generalized to Asian-American Buddhist communities, which may or may not have analogous approaches to meditation practice and social dynamics.

The sample is also non-representative in terms of demographics and related health risks. With 73% earning graduate or doctoral degrees, the sample represents the top 5–10% of educational attainment [170], and lower-than-average rates of mental illness and trauma exposure [171, 172], which are known to be inversely correlated to educational attainment [173, 174]. Thus, the findings of the current study may be able to generalize to other highly educated, low-risk samples, but not to clinical or other disadvantaged samples with high levels of mental or physical illness or trauma exposure.

While the current practitioner sample had slightly more men than women, this gender difference is an artifact of sampling and does not reflect the relative frequency of meditation-related difficulties across genders. Because our sampling approach is stratified by gender, this study will be able to describe how gender interacted with different meditation experiences, but will not be able to speak to other demographic variables such as race or sexual orientation. Further research in these various populations is necessary to determine frequency, generalizability, and causality of the phenomena reported in this study.

#### Category frequency

Practitioners reported their meditation-related experiences based upon open-ended questions, and were generally not prompted or directly queried to report or discuss specific types of experiences by interviewers. As a result, the frequency of reported categories may reflect not only relative frequency within our sample but also factors related to the participants as well as methodology. In terms of participant factors, the frequency of reported categories is likely a function of salience and effability, which is also a function of distress and available interpretive frameworks [175]. In other words, categories that are repeatedly reported may not only be frequent, but also may be the most important to participants. Conversely, some infrequently reported experiences may not be reported because they were not considered important or were difficult to describe. Practitioners most likely had a wider range of experiences than they chose to report.

The duration between the onset of meditation-related difficulties and the time of interview is another variable affecting both category frequency and the richness of phenomenological descriptions. Practitioners who provided interviews immediately following or still in the midst of meditation-related difficulties were often able to provide the most detailed accounts. Thus, it is likely that the more temporally distant interview subjects underreported their phenomenology, as only the most salient features were recalled. Such interview subjects were also more likely to have greater difficulty isolating a descriptive phenomenology from subsequent interpretive frameworks. However, practitioners who had more distance from their difficulties provided more information on how they navigated, interpreted, managed, or integrated those difficulties, and thus these subjects contributed valuable information on influencing factors that was not always available from interview subjects still in the midst of difficulties. The strengths and weakness of both types of interviews should be taken into account when sampling further populations.

In terms of methodological factors, the coding structure and the broadness or narrowness of the scope of a given category also affects the calculation of frequency. Some categories are currently an amalgamation of related phenomena that will be further disambiguated in subsequent phases of analysis, whereas other categories (notably those in the sense of self domain) are already delineated quite narrowly. In addition, some categories always refer only to a primary meditation-related experience, whereas others (such as fear, anger, or self-conscious emotions) include not only primary experiences, but also secondary responses to different primary experiences (such as fear arising due to a loss of sense of agency), as well as tertiary responses to how meditation-related challenges played out in social and interpersonal domains (such as shame or anger arising due to how a meditator in distress was treated by a meditation community). Thus, the frequency of categories in the current study should be regarded as preliminary and not conclusive. Future studies should use structured questionnaires to more accurately assess category-specific frequency and causality both within and across samples.

#### Causality assessment

The best study design for concluding that any experience—positive or negative—is caused by meditation is a prospective randomized controlled trial (RCT) that uses a validated measure before and after learning meditation or the equal passage of time in a matched sample. In addition, since many effects, including the ones reported in the current study, may not occur until many years after beginning a meditation practice, a conclusive study would also need a multi-year follow-up to produce the most accurate frequency estimates. Given that such extensive study designs are rare to non-existent in the current state of meditation research, researchers and policymakers use alternative methods. The current study assessed meditation causality according 11 of the 13 criteria that regulatory agencies use when making policy health decisions about possible adverse effects of medical procedures in instances where prospective trial and epidemiological data are not available [107–110]. The results provided causality-related evidence for all 11 criteria.

The remaining two criteria, biological gradient and the linkage to known biological mechanisms, are being investigated by our group and others and will be described in more detail in subsequent publications. Briefly, biological gradient, or that “greater exposure should lead to greater incidence of the effect” [114, 115], is being investigated by our group by using the current study’s codebook categories in a clinical trial of MBCT [176]. Links to biological mechanisms are best investigated separately for discrete experiences or related classes of experience. Lindahl et al. (2014) [126] provided a preliminary report on qualitative data from the VCE project and a neurobiological model of one category of perceptual changes (visual lights). We speculate that this model (homeostatic neuroplasticity) may account for a number of other categories in other domains. In a review paper, Britton et al. (2014) [129] provided a neurobiological model for sleep-related changes. Future papers from the VCE data set will continue to draw from available behavioral, psychological, and neurobiological research to better understand potential mechanisms of action. Similarly, other researchers have provided neurobiological models closely related to specific categories in this study, particularly changes in sense of self [78] and altered perceptions of space and time [77].

Finally, it is important to specify what claims can and cannot be made based upon the causality assessment results of this study. First, the results do reduce the likelihood that all of the experiences reported were entirely unrelated to meditation, or only reflect a pre-existing condition that happened to co-occur with meditation practice. Similarly, the results also challenge other common causal attributions, such as the assumption that meditation-related difficulties *only* happen to individuals with a pre-existing condition (psychiatric or trauma history), who are on long or intensive retreats, who are poorly supervised, who are practicing incorrectly, or who have inadequate preparation. However, this is not to say that these and other factors do not play a role. Indeed, both experts and practitioners identified various “influencing factors” that they thought impacted the likelihood of meditation-related challenges, their duration, and their associated degree of distress and impairment. These data are most suggestive of an interaction-based model where meditation practices—on their own—may produce challenging effects, but the specific type of effect, as well as its likelihood, duration, and associated distress and impairment, is influenced by a number of additional factors. Finally, given that this is one of the first studies of this scope on this topic, these results should not be interpreted as conclusive; rather, they should be taken as sufficient evidence to warrant further investigation.

### Future directions

The current paper describes the methodology, coding structure, and basic findings of the Varieties of Contemplative Experience study. Future papers will address specific aspects of both phenomenology and influencing factors as well as their relationship to each other. When focusing on specific categories or domains of phenomenology and/or influencing factors, future publications will engage directly with coded interview content, and categories that currently have extensive and diverse content will be subject to further thematic content analysis through the creation of data-driven and theory-driven subcategories. As practiced in a paper of preliminary data on visual phenomena [126], VCE study publications also aim to generate empirically tractable neurobiological hypotheses on the range of effects associated with meditation by engaging with scientific literature on meditation in tandem with research on related experiences from psychological and neuroscientific studies of phenomena such as depersonalization disorder, trauma, or sensory deprivation, for example. The interdisciplinary approach adopted in this study also requires attending to how such experiences are appraised and potentially differentiated from similar phenomena, in both Buddhist textual sources as well as in the reports of practitioners and experts in the study. Hypotheses generated from reports of understudied meditation-related phenomena can then be used to guide future research in the psychology and neuroscience of meditation.

The study provides initial descriptions of experience that warrant further investigation in both research and clinical settings. For more detailed information about the strength and direction of influence, the field needs well-controlled longitudinal studies on meditators employing a questionnaire assessing meditation-related experiences. The taxonomy of meditation-related experiences developed through the current study forms the foundation for such a questionnaire. An instrument based upon the phenomenology codebook has been pilot tested in a randomized controlled trial of Mindfulness-Based Cognitive Therapy [176] and is undergoing revision for wide-scale dissemination in future meditation research studies.

Three studies that follow the VCE study methodology are also currently underway. One study is a sample of practitioners and teachers from Jewish, Christian, and Sufi traditions (PI: NF). Comparisons between the core VCE data set of Buddhist practitioners and the practitioners from Abrahamic traditions will facilitate an understanding of which types of experiences may be unique to a particular practice tradition, as well as which experiences are reported in common, despite differences in contemplative practices. It will also illuminate the importance of worldviews and conceptual frameworks in the interpretation of experiences that are common to both data sets. A second study of European Buddhist meditators is also underway. Comparisons between this study and our data set, which is largely comprised of American practitioners, has the potential to enhance our understanding of the influence of sociocultural context, isolating variables that might be unique to American and European contexts, respectively. Similarly, a third study will compare meditation practitioners and experts in the United States with practitioners and experts of the same tradition in India in order to assess the influence of cultural contexts on phenomenology and appraisals.

Finally, the study also aims to provide resources and practical methods to facilitate the integration of difficult experiences that meditation practitioners report as being initially unexpected or challenging. Future papers will investigate particularly distressing and functionally impairing symptoms along with an assessment of potential risk factors and possible remedies. Subsequent analyses will also examine the convergences as well as the discrepancies between practitioners’ experiences and experts’ awareness of and management of those experiences. We have partnered with a number of meditation centers in the US and Europe and are offering educational materials and trainings as part of their meditation teacher-training programs. These resources could be of benefit not only to practitioners and meditation instructors, but also to the burgeoning application of meditation techniques in psychology and medicine.

## Supporting information

S1 File Practitioner interview questions. (PDF)

S2 File Expert interview questions. (PDF)

S3 File Data file. (XLSX)

S4 File Phenomenology codebook. (PDF)

S5 File Influencing factors codebook. (PDF)

## References

[1] Lindahl JR. Why right mindfulness might not be right for mindfulness. Mindfulness. 2015;6:57–62.

[2] Goyal M, Singh S, Sibinga EM, Gould NF, Rowland-Seymour A, Sharma R, et al Meditation programs for psychological stress and well-being: a systematic review and meta-analysis. JAMA internal medicine. 2014;174(3):357–68. PubMed Central PMCID: PMC4142584. doi: 10.1001/jamainternmed.2013.13018 24395196 10.1001/jamainternmed.2013.13018 PMC4142584

[3] Chiesa A, Serretti A. Are mindfulness-based interventions effective for substance use disorders? A systematic review of the evidence. Substance use & misuse. 2014;49(5):492–512. 23461667 10.3109/10826084.2013.770027

[4] Grant JA. Meditative analgesia: the current state of the field. Annals of the New York Academy of Sciences. 2014;1307:55–63. doi: 10.1111/nyas.12282 24673150 10.1111/nyas.12282

[5] Hofmann SG, Sawyer AT, Witt AA, Oh D. The effect of mindfulness-based therapy on anxiety and depression: A meta-analytic review. Journal of consulting and clinical psychology. 2010;78(2):169–83. PubMed Central PMCID: PMC2848393. doi: 10.1037/a0018555 20350028 10.1037/a0018555 PMC2848393

[6] Chiesa A, Serretti A. Mindfulness based cognitive therapy for psychiatric disorders: a systematic review and meta-analysis. Psychiatry research. 2011;187(3):441–53. doi: 10.1016/j.psychres.2010.08.011 20846726 10.1016/j.psychres.2010.08.011

[7] Chiesa A, Serretti A. Mindfulness-based interventions for chronic pain: a systematic review of the evidence. Journal of alternative and complementary medicine. 2011;17(1):83–93. doi: 10.1089/acm.2009.0546 21265650 10.1089/acm.2009.0546

[8] Chiesa A, Calati R, Serretti A. Does mindfulness training improve cognitive abilities? A systematic review of neuropsychological findings. Clin Psychol Rev. 2011;31(3):449–64. doi: 10.1016/j.cpr.2010.11.003 21183265 10.1016/j.cpr.2010.11.003

[9] Chambers R, Gullone E, Allen NB. Mindful emotion regulation: An integrative review. Clinical psychology review. 2009;29(6):560–72. doi: 10.1016/j.cpr.2009.06.005 19632752 10.1016/j.cpr.2009.06.005

[10] Samuelson M, Carmody J, Kabat-Zinn J, Bratt MA. Mindfulness-Based Stress Reductioin in Massachusetts Correctional Facilities. The Prison Journal. 2007;2:254–68.

[11] Sumter MT, Monk-Turner E, Turner C. The benefits of meditation practice in the correctional setting. J Correct Health Care. 2009;15(1):47–57; quiz 81. doi: 10.1177/1078345808326621 19477811 10.1177/1078345808326621

[12] Jha AP, Stanley EA, Kiyonaga A, Wong L, Gelfand L. Examining the protective effects of mindfulness training on working memory capacity and affective experience. Emotion. 2010;10(1):54–64. doi: 10.1037/a0018438 20141302 10.1037/a0018438

[13] Stanley EA, Schaldach JM, Kiyonaga A, Jha AP. Mindfulness-based Mind Fitness Training: A Case Study of a High-Stress Predeployment Military Cohort. Cognitive and Behavioral Practice 2011;18(4):566–76.

[14] Stanley EA, Jha AP. Mind fitness: Improving operational effectiveness and building warrior resilience. Joint Force Quarterly. 2009;55:144–51.

[15] MLERN, Davidson R, Dunne J, Eccles J, Engle A, Greenberg M, et al Contemplative Practices and Mental Training: Prospects for American Education. Child Dev Perspect. 2012;6(2):146–53. Epub 2012/08/21. PubMed Central PMCID: PMC3420012. doi: 10.1111/j.1750-8606.2012.00240.x 22905038 10.1111/j.1750-8606.2012.00240.x PMC3420012

[16] Kaiser-Greenland S. The Mindful Child. New York Free Press; 2010.

[17] Greenberg M, Harris A. Nurturing Mindfulness in Children and Youth: Current State of Research. Child Dev Perspect. 2012; 6(2):161–6.

[18] Meiklejohn J, Phillips C, Freedman M, Griffin M, Biegel G, Roach A, et al Integrating Mindfulness Training into K-12 Education: Fostering the Resilience of Teachers and Students. Mindfulness. 2012:1–17.

[19] Shapiro S, Brown K, Astin J. Toward the Integration of Meditation into Higher Education: A Review of Research Evidence. Teachers College Record. 2011;113(3): 493–528.

[20] Mani M, Kavanagh DJ, Hides L, Stoyanov SR. Review and Evaluation of Mindfulness-Based iPhone Apps. JMIR Mhealth Uhealth. 2015;3(3):e82 doi: 10.2196/mhealth.4328 26290327 10.2196/mhealth.4328 PMC4705029

[21] Wieczner J. Meditation Has Become A Billion-Dollar Business. Fortune. 2016;3 12:1–2.

[22] Clarke TC, Black LI, Stussman BJ, Barnes PM, Nahin RL. Trends in the use of complementary health approaches among adults: United States 2002–2012 National Health Statistics Reports no 79. Hyattsville, MD: National Center for Health Statistics; 2015. PMC4573565 25671660

[23] Barnes P, Bloom B, Nahin R. Complementary and Alternative Medicine Use Among Children and Adults: United States, 2007. CDC National Health Statisitics Report #12. 2008:1–24. 19361005

[24] Teasdale JDC, M. How does mindfulness transform suffering? II: the transformation of dukkha. Contemporary Buddhism. 2011;12:103–24.

[25] Kabat-Zinn J. Some Reflections on the Origins of MBSR, Skillful Means, and the Trouble with Maps. Contemporary Buddhism. 2011;12:281–306.

[26] Grant JA, Rainville P. Pain sensitivity and analgesic effects of mindful states in Zen meditators: a cross-sectional study. Psychosomatic medicine. 2009;71(1):106–14. doi: 10.1097/PSY.0b013e31818f52ee 19073756 10.1097/PSY.0b013e31818f52ee

[27] MacLean KA, Ferrer E, Aichele SR, Bridwell DA, Zanesco AP, Jacobs TL, et al Intensive meditation training improves perceptual discrimination and sustained attention. Psychological science. 2010;21(6):829–39. PubMed Central PMCID: PMCPMC3132583. doi: 10.1177/0956797610371339 20483826 10.1177/0956797610371339 PMC3132583

[28] Kaliman P, Alvarez-Lopez MJ, Cosin-Tomas M, Rosenkranz MA, Lutz A, Davidson RJ. Rapid changes in histone deacetylases and inflammatory gene expression in expert meditators. Psychoneuroendocrinology. 2014;40:96–107. PubMed Central PMCID: PMCPMC4039194. doi: 10.1016/j.psyneuen.2013.11.004 24485481 10.1016/j.psyneuen.2013.11.004 PMC4039194

[29] Sudsuang R, Chentanez V, Veluvan K. The effect of buddhist meditation on serum cortisol and total protein levels, blood pressure, pulse rate, lung volume and reaction time. Physiol Behav. 1991;50:543–8. 1801007 10.1016/0031-9384(91)90543-w

[30] Lopez D. The Scientific Buddha: His Short and Happy Life. New Haven: Yale University Press; 2012.

[31] Lopez DS Jr. Buddhism and science: A guide for the perplexed: University of Chicago Press; 2009.

[32] McMahan D. Buddhism as the “religion of science": From colonial Ceylon to the laboratories of Harvard. In: Lewis JR, Hammer O, editors. Handbook of Religion and the Authority of Science Leiden: Brill; 2011.

[33] McMahan D. The Making of Buddhist Modernism New York: Oxford University Press; 2008.

[34] Sharf R. Is mindfulness Buddhist? (and why it matters). Transcultural Psychiatry. 2014;52(4):470–84. doi: 10.1177/1363461514557561 25361692 10.1177/1363461514557561

[35] Wilson J. Mindful America: The Mutual Transformation of Buddhist Meditation and American Culture New York: Oxford University Press; 2014.

[36] Huntington CW. The Triumph of Narcissism: Theravāda Buddhist Meditation in the Marketplace. Journal of the American Academy of Religion 2015;83:624–48.

[37] Wallace B. Stilling the Mind: Shamatha Teachings from Dudjom Lingpa’s Vajra Essence. Boston: Wisdom Publications; 2011.

[38] Wallace BA. Dudjom Lingpa’s Visions of the Great Perfection, Volume 3: The Vajra Essence Boston: Wisdom Publications; 2015.

[39] Gyatso J. Healing burns with fire: The facilitations of experience in Tibetan Buddhism. Journal of the American Academy of Religion 1999;67(1):113–47.

[40] Sogen O. An Introduction to Zen training. (D. Hosokawa, Trans.) Boston: Tuttle Publishing; 2001.

[41] Aitken R. Taking the Path of Zen. San Francisco: North Point Press; 1982.

[42] Hakuin. Idle talk on a night boat. In: Waddell N, editor. Hakuin’s Precious Mirror Cave. Berkeley: Counterpoint; 2009.

[43] Hua H. The Shurangama Sutra with commentary, Vol. 8 Burlingame, CA: Buddhist Text Publication Society; 2003.

[44] Buddhaghosa B. The Path of Purification. Onalaska, WA: Buddhist Publication Society; 1991.

[45] Sayadaw M. Manual of insight. Somerville, MA: Wisdom Publications; 2016.

[46] Tate A. The Autobiography of a Forest Monk. Chiang Mai: Wat Hin Mark Peng; 1993.

[47] Sayadaw M. The Progress of Insight: A Modern Pali Treatise on Buddhist Satipatthana Meditation. Kandy, Sri Lanka: Buddhist Publication Society; 1965.

[48] Namto S. Insight Meditation: Practical Steps to Ultimate Truth. Fawnskin, CA: Vipassana Dhura Meditation Society; 1989.

[49] Lindahl JR. Self-transformation according to Buddhist stages of the path literature. Pacific World: Journal of the Insitute of Buddhist Studies. 2012;3(14):231–75.

[50] Sharf R. Experience In: Taylor MC, editor. Critical Terms for Religious Studies. Chicago: University of Chicago Press; 1998.

[51] Sharf R. Buddhist modernism and the rhetoric of meditative experience. Numen. 1995;42:228–83.

[52] Wallace BA. The Attention Revolution: Unlocking the Power of the Focused Mind. Boston: Wisdom; 2006.

[53] Kornfield J. Bringing home the Dharma: Awakening right where you are. Boston: Shambhala Publications; 2011.

[54] Jonsson U, Alaie I, Parling T, Arnberg FK. Reporting of harms in randomized controlled trials of psychological interventions for mental and behavioral disorders: A review of current practice. Contemporary clinical trials. 2014;38(1):1–8. Epub 2014/03/13. doi: 10.1016/j.cct.2014.02.005 24607768 10.1016/j.cct.2014.02.005

[55] Fowler F. Mode effcts in a survey of Medicare prostate surgery patients. Public Opinion Quarterly. 1998;62:29–46.

[56] Turner C, Lessler J, George B, Hubbard M, Watt M. Effects of mode of administration and wording on reporting of drug use In: Turner CF, Lessler JT, Gfroerer JC, editors. Survey Measurement of Drug Use: Methodological Studies. Washington, D.C: Government Printing Office.; 1992 p. 177–220.

[57] Weissman JS, Schneider EC, Weingart SN, Epstein AM, David-Kasdan J, Feibelmann S, et al Comparing patient-reported hospital adverse events with medical record review: do patients know something that hospitals do not? Annals of internal medicine. 2008;149(2):100–8. Epub 2008/07/16. 18626049 10.7326/0003-4819-149-2-200807150-00006

[58] Bent S, Padula A, Avins AL. Brief communication: Better ways to question patients about adverse medical events: a randomized, controlled trial. Annals of internal medicine. 2006;144(4):257–61. Epub 2006/02/24. 16490911 10.7326/0003-4819-144-4-200602210-00007

[59] Kuyken W, Hayes R, Barrett B, Byng R, Dalgleish T, Kessler D, et al Effectiveness and cost-effectiveness of mindfulness-based cognitive therapy compared with maintenance antidepressant treatment in the prevention of depressive relapse or recurrence (PREVENT): a randomised controlled trial. Lancet. 2015;386(9988):63–73. doi: 10.1016/S0140-6736(14)62222-4 25907157 10.1016/S0140-6736(14)62222-4

[60] Kuyken W, Warren FC, Taylor RS, Whalley B, Crane C, Bondolfi G, et al Efficacy of Mindfulness-Based Cognitive Therapy in Prevention of Depressive Relapse: An Individual Patient Data Meta-analysis From Randomized Trials. JAMA Psychiatry. 2016. 10.1001/jamapsychiatry.2016.0076 PMC6640038 27119968

[61] Williams JM, Crane C, Barnhofer T, Brennan K, Duggan DS, Fennell MJ, et al Mindfulness-based cognitive therapy for preventing relapse in recurrent depression: a randomized dismantling trial. Journal of consulting and clinical psychology. 2014;82(2):275–86. PubMed Central PMCID: PMCPMC3964149. doi: 10.1037/a0035036 24294837 10.1037/a0035036 PMC3964149

[62] Kuyken W, Crane W, Williams JM. Mindfulness-Based Cognitive Therapy (MBCT) Implementation Resources Oxford University, University of Exeter, Bangor University 2012.

[63] Lustyk M, Chawla N, Nolan R, Marlatt G. Mindfulness Medtation Research: Issues of participant screening, safety procedures, and researcher training. Advances in Mind-Body Medicine. 2009;24(1):20–30. 20671334

[64] Kuijpers H, van der Heijden F, Tuinier S, Verhoeven W. Meditation-induced psychosis. Psychopathology. 2007;40:461–4. doi: 10.1159/000108125 17848828 10.1159/000108125

[65] Epstein M, Lieff J. Psychiatric complications of meditation practice. The Journal of Transpersonal Psychology. 1981;13(2):137–47.

[66] Jaseja H. Potential role of self-induced EEG fast oscillations in predisposition to seizures in meditators. Epilepsy & Behavior. 2010;17:124–5. 19932061 10.1016/j.yebeh.2009.10.022

[67] Shapiro DH Jr. Adverse effects of meditation: a preliminary investigation of long-term meditators. Int J Psychosom. 1992;39(1–4):62–7. 1428622

[68] Yorston G. Mania precipitated by meditation: a case report and literature review. Mental Health, Religion & Culture. 2001;4:209–14.

[69] Miller J. The unveling of traumatic memories and emotions through mindfulness and concentration meditation: clinical implications and three case reports. Journal of Transpersonal Psychology. 1993;25:169–80.

[70] Chan-Ob T, Boonyanaruthee V. Meditation in association with psychosis. Journal of the Medical Association of Thailand. 1999;82(9):925–30. 10561951

[71] Kutz I, Leserman J, Dorrington C, Morrison CH, Borysenko JZ, Benson H. Meditation as an adjunct to psychotherapy. An outcome study. Psychother Psychosom. 1985;43(4):209–18. 3898186 10.1159/000287881

[72] Walsh R, Roche L. Precipitation of acute psychotic episodes by intensive meditation in individuals with a history of schizophrenia. Am J Psychiatry. 1979;136(8):1085–6. doi: 10.1176/ajp.136.8.1085 380368 10.1176/ajp.136.8.1085

[73] Disayavanish C, Disayavanish P. Meditation-induced psychosis (in Thai). Journal of the Psychiatric Association of Thailand. 1984;29:1–12.

[74] Turner RP, Lukoff D, Barnhouse RT, Lu FG. Religious or spiritual problem. A culturally sensitive diagnostic category in the DSM-IV. The Journal of nervous and mental disease. 1995;183(7):435–44. 7623015 10.1097/00005053-199507000-00003

[75] APA. 300.6 Depersonalization Disorder. Diagnostic and Statistical Manual of Mental Disorders (DSM IV). 4th Edition ed. Washington DC: American Psychiatric Association; 1994.

[76] APA. 300.6 Depersoanlization/Derealization Disorder. Diagnostic and Staistical Manual of Mental Disorders, 5th Edition (DSM-5). Arlington VA: American Psychiatric Association; 2013. p. 302–6.

[77] Berkovich-Ohana A, Dor-Ziderman Y, Glicksohn J, Goldstein A. Alterations in the sense of time, space, and body in the mindfulness-trained brain: a neurophenomenologically-guided MEG study. Front Psychol. 2013;4:912 PubMed Central PMCID: PMCPMC3847819. doi: 10.3389/fpsyg.2013.00912 24348455 10.3389/fpsyg.2013.00912 PMC3847819

[78] Dor-Ziderman Y, Berkovich-Ohana A, Glicksohn J, Goldstein A. Mindfulness-induced selflessness: a MEG neurophenomenological study. Front Hum Neurosci. 2013;7:582 PubMed Central PMCID: PMCPMC3781350. doi: 10.3389/fnhum.2013.00582 24068990 10.3389/fnhum.2013.00582 PMC3781350

[79] Ataria Y, Dor-Ziderman Y, Berkovich-Ohana A. How does it feel to lack a sense of boundaries? A case study of a long-term mindfulness meditator. Conscious Cogn. 2015;37:133–47. doi: 10.1016/j.concog.2015.09.002 26379087 10.1016/j.concog.2015.09.002

[80] Chen Z, Qi W, Hood R, Watson P. Common Core Thesis and Qualitative and Quantitative Analysis of Mysticism in Chinese Buddhist Monks and Nuns. Journal for the Scientific Study of Religion. 2011;50(4):654–70.

[81] Ataria Y. Where do we end and where does the world begin? The case of insight meditation. Philosophical Psychology. 2015;28(8):1128–46.

[82] Droit-Volet S, Fanget M, Dambrun M. Mindfulness meditation and relaxation training increases time sensitivity. Conscious Cogn. 2015;31:86–97. doi: 10.1016/j.concog.2014.10.007 25460243 10.1016/j.concog.2014.10.007

[83] Full G, Walach H, Trautwein M. Meditation induced changes in perception: An interview study with expert meditators (sotapannas) in Burma. Mindfulness. 2013;4:55–63.

[84] Kornfield J. Intensive insight meditation: A phenomenological study. Journal of Transpersonal Psychology. 1979.

[85] Lomas T, Cartwright T, Edginton T, Ridge D. A qualitative summary of experiential challenges associated with meditation practice. Mindfulness. 2014:1–13.

[86] VanderKooi L. Buddhist teachers’ experience with extreme mental states in Western meditators. Journal of Transpersonal Psychology. 1997;29:31–46.

[87] DeHoff S. Distinguishing Mystical Religious Experience and Psychotic Experience: A Qualitative Study Interviewing Presbyterian Church (U.S.A.) Professionals. Pastoral Psychology. 2015;64:21–39.

[88] O’Connor S, Vandenburg B. Differentiating psychosis and faith: the role of social norms and religious fundamentalism. Mental Health, Religion & Culture 2010;13(2):171–86.

[89] Menezes A, and Moreira-Almeida A. Religion, Spirituality, and Psychosis. Current Psychiatry Reports. 2010;12:174–9. doi: 10.1007/s11920-010-0117-7 20425277 10.1007/s11920-010-0117-7

[90] Dein S, Littlewood R. Religion and psychosis: A common evolutionary trajectory?. Transcultural Psychiatry 2011;48(3):318–35. doi: 10.1177/1363461511402723 21742955 10.1177/1363461511402723

[91] Pasick R., Burke N., Barker J., Joseph G., Bird J., Otero-Sabogal R., Tuason N., Stewart S., Rakowski W., Clark M., Washington P., & Guerra C. (2009). Behavioral Theory in a Diverse Society: Like a Compass on Mars. Health Education and Behavior, 36, 11S–35S. 19805789 10.1177/1090198109338917 PMC2921832

[92] Patton M. Qualitative research and evaluation methods 3rd ed. Thousand Oaks, CA: Sage Publications; 2002.

[93] Taves A. Ascription, attribution, and cognition in the study of experiences deemed religious. Religion. 2008;38:125–40.

[94] Taves A. Religious Experience Reconsidered: A Building Block Approach to the Study of Religion and Other Special Things. Princeton: Princeton University Press; 2009.

[95] Kirmayer L, Ban L. Cultural Psychiatry In: Alarcon R, editor. Cultural Psychiatry. Advances in Psychosomatic Medicine. 33 Basel, Switzerland: Karger; 2013. 10.1159/000348742 23816867

[96] Monteiro LM, Musten R. F., & Compson J. Traditional and contemporary mindfulness: finding the middle path in the tangle of concerns. Mindfulness. 2015;6:1–13.

[97] Shonin E, Van Gordon W., & Griffiths M. D. Meditation Awareness Training (MAT) for improved psychological well-being: a qualitative examination of participant experiences. Journal of Religion and Health. 2014;53(3):849–63. doi: 10.1007/s10943-013-9679-0 23377964 10.1007/s10943-013-9679-0

[98] Tashakkori A, Teddlie C, editors. Handbook of mixed methods in social & behavioral research Thousand Oaks, CA: Sage; 2003.

[99] Teddlie C, Yu F. Mixed Methods Sampling: A Typology With Examples. Journal of Mixed Methods Research. 2007;1(1):77–100.

[100] Faugier J, Sargeant M. Sampling hard to reach populations. Journal of Advanced Nursing. 1997;26:790–7. 9354993 10.1046/j.1365-2648.1997.00371.x

[101] Guest G, MacQueen KM, Namey EE. Applied Thematic Analysis. Thousand Oaks, CA: Sage Publications 2012.

[102] Kessler RC, McGonagle KA, Zhao S, Nelson CB, Hughes M, Eshleman S, et al Lifetime and 12-month prevalence of DSM-III-R psychiatric disorders in the United States. Results from the National Comorbidity Survey. Arch Gen Psychiatry. 1994;51(1):8–19. 8279933 10.1001/archpsyc.1994.03950010008002

[103] Robins L, Locke B, Regier D. An overview of psychiatric disorders in America In: Robins L, Regier D, editors. Psychiatric Disorders in America: The Epidemiological Catchment Study. New York: Free Press; 1991.

[104] Bovin MJ, Marx BP, Weathers FW, Gallagher MW, Rodriguez P, Schnurr PP, et al Psychometric Properties of the PTSD Checklist for Diagnostic and Statistical Manual of Mental Disorders-Fifth Edition (PCL-5) in Veterans. Psychol Assess. 2015. 10.1037/pas0000254 26653052

[105] McFarlane A, Clark CR, Bryant RA, Williams LM, Niaura R, Paul RH, et al The impact of early life stress on psychophysiological, personality and behavioral measures in 740 non-clinical subjects. J Integr Neurosci. 2005;4(1):27–40. 16035139 10.1142/s0219635205000689

[106] Bernstein DP, Stein JA, Newcomb MD, Walker E, Pogge D, Ahluvalia T, et al Development and validation of a brief screening version of the Childhood Trauma Questionnaire. Child abuse & neglect. 2003;27(2):169–90. 12615092 10.1016/s0145-2134(02)00541-0

[107] NIH. Adverse Event and Serious Adverse Event Guidelines. OHRP Guidance on Reviewing and Reporting Unanticipated Problems Involving Risks to Subjects or Others and Adverse Events, OHRP Guidance. National Insitutes of Health (NIH): Office for Human Research Protections, U.S. Department of health and Human Servcies; 2016.

[108] Agbabiaka TB, Savovic J, Ernst E. Methods for causality assessment of adverse drug reactions: a systematic review. Drug safety. 2008;31(1):21–37. 18095744 10.2165/00002018-200831010-00003

[109] Turner WM. The Food and Drug Administration algorithm. Special workshop—regulatory. Drug information journal. 1984;18(3–4):259–66. 10268553 10.1177/009286158401800311

[110] WHO. The use of the WHO-UMC system for standardized case causaility assessment. who-umc.org: World Health Organization (WHO), Uppsala Monitoring Centre 2016.

[111] Naranjo CA. A clinical pharmacologic perspective on the detection and assessment of adverse drug reactions. Drug information journal. 1986;20(4):387–93. 24482869 10.1177/009286158602000403

[112] Naranjo CA, Busto U, Sellers EM. Difficulties in assessing adverse drug reactions in clinical trials. Progress in neuro-psychopharmacology & biological psychiatry. 1982;6(4–6):651–7. 6761768 10.1016/s0278-5846(82)80162-0

[113] Naranjo CA, Busto U, Sellers EM, Sandor P, Ruiz I, Roberts EA, et al A method for estimating the probability of adverse drug reactions. Clinical pharmacology and therapeutics. 1981;30(2):239–45. Epub 1981/08/01. 7249508 10.1038/clpt.1981.154

[114] Hill AB. The Environment and Disease: Association or Causation? Proceedings of the Royal Society of Medicine. 1965;58:295–300. PubMed Central PMCID: PMC1898525. 14283879 10.1177/003591576505800503 PMC1898525

[115] Hill AB. The environment and disease: association or causation? Journal of the Royal Society of Medicine. 2015;108(1):32–7. doi: 10.1177/0141076814562718 25572993 10.1177/0141076814562718 PMC4291332

[116] Gallagher RM, Kirkham JJ, Mason JR, Bird KA, Williamson PR, Nunn AJ, et al Development and inter-rater reliability of the Liverpool adverse drug reaction causality assessment tool. PLoS One. 2011;6(12):e28096 PubMed Central PMCID: PMC3237416. doi: 10.1371/journal.pone.0028096 22194808 10.1371/journal.pone.0028096 PMC3237416

[117] Theophile H, Arimone Y, Miremont-Salame G, Moore N, Fourrier-Reglat A, Haramburu F, et al Comparison of three methods (consensual expert judgement, algorithmic and probabilistic approaches) of causality assessment of adverse drug reactions: an assessment using reports made to a French pharmacovigilance centre. Drug safety. 2010;33(11):1045–54. doi: 10.2165/11537780-000000000-00000 20925441 10.2165/11537780-000000000-00000

[118] OHRP. Guidance on Reviewing and Reporting Unanticipated Problems Involving Risks to Subjects or Others and Adverse Events: Office for Human Research Protections, US Department of Health and Human Services; 2007.

[119] NIA. NIA Adverse Event and Serious Adverse Event Guidelines. National Institutes on Aging, National Insitutes of Health (NIH)2011.

[120] Elo S, Kyngas H. The qualitative content analysis process. Journal of advanced nursing. 2008;62(1):107–15. doi: 10.1111/j.1365-2648.2007.04569.x 18352969 10.1111/j.1365-2648.2007.04569.x

[121] Strauss A, Corbin J. Basics of qualitative research: Techniques and procedures for developing grounded theory. Thousand Oaks, CA: Sage Publications.; 1998.

[122] DeCuir-Gunby J. Developing and using a codebook for the analysis of interview data: An example from a professional development research project. Field Methods 2011;23(2):136–55.

[123] Kriegel U. The Varieties of Consciousness. Oxford: Oxford University Press: Oxford University Press; 2015.

[124] Fonteyn ME, Vettese M, Lancaster DR, Bauer-Wu S. Developing a codebook to guide content analysis of expressive writing transcripts. Applied nursing research: ANR. 2008;21(3):165–8. doi: 10.1016/j.apnr.2006.08.005 18684411 10.1016/j.apnr.2006.08.005

[125] MacQueen K, McLellan E, Kay K, Milstein B. Codebook development for team-based qualitative analysis. Cultural Anthropology Methods 1998;10(31–36).

[126] Lindahl J, Kaplan C, Winget E, Britton W. A Phenomenology of Meditation-Induced Light Experiences: Traditional Buddhist and Neurobiological Perspectives. Frontiers in Psychology. 2014;4(973). 10.3389/fpsyg.2013.00973 PMC3879457 24427148

[127] Simeon D. Depersonalization disorder. In: Dell PFaON, J.A., editor. Dissociation and the Dissociative Disorders: DSM-V and Beyond. New York: Routledge; 2009.

[128] Blom JD. Hallucinations and other sensory deceptions in psychiatric disorders In: Jardri R, Cachia A, Thomas P, D P, editors. The Neuroscience of Hallucinations. New York: Spinger; 2013 p. 43–58.

[129] Britton WB, Lindahl JR, Cahn BR, Davis JH, Goldman RE. Awakening is not a metaphor: the effects of Buddhist meditation practices on basic wakefulness. Annals of the New York Academy of Sciences. 2014;1307:64–81. PubMed Central PMCID: PMC4054695. doi: 10.1111/nyas.12279 24372471 10.1111/nyas.12279 PMC4054695

[130] Gallagher S, editor. The Oxford Handbook of the Self. Oxford: Oxford University Press; 2013.

[131] Parnas J, Moller P, Kircher T, Thalbitzer J, Jansson L, Handest P, et al EASE: Examination of Anomalous Self-Experience. Psychopathology. 2005;38(5):236–58. doi: 10.1159/000088441 16179811 10.1159/000088441

[132] Cermolacce M, Naudin J, Parnas J. The "minimal self" in psychopathology: re-examining the self-disorders in the schizophrenia spectrum. Conscious Cogn. 2007;16(3):703–14. doi: 10.1016/j.concog.2007.05.013 17632013 10.1016/j.concog.2007.05.013

[133] Castillo R. Depersonalization and meditation. Psychiatry Research: Neuroimaging Section. 1990;53:158–68. 10.1080/00332747.1990.11024497 2191357

[134] Sethi S. Relationship of meditation and psychosis: case studies. Australian and New Zealand Journal of Psychiatry. 2003;37(3):382. 10.1046/j.1440-1614.2003.11721.x 12780479

[135] Heide F, Borkovec T. Relaxation-induced anxiety: paradoxical anxiety enhancement due to relaxation treatment. Journal of consulting and clinical psychology. 1983;51(2):171–82. 6341426 10.1037//0022-006x.51.2.171

[136] Carrington P. The Misuse of Meditation: Problems from Overmeditation Freedom in Meditation. Garden City, NY: Anchor Books; 1977.

[137] Shonin E, Van Gordon W, Griffiths MD. Do mindfulness-based therapies have a role in the treatment of psychosis? Aust N Z J Psychiatry. 2014;48(2):124–7. doi: 10.1177/0004867413512688 24220133 10.1177/0004867413512688

[138] Brown D, Forte M, Dysart M. Visual sensitivity and mindfulness meditation. Percept Mot Skills. 1984;58(3):775–84. doi: 10.2466/pms.1984.58.3.775 6382145 10.2466/pms.1984.58.3.775

[139] Deikman A. Implications of experimentally induced contemplative meditation. Journal of Nervous and Mental Disease. 1966;142:101–16. 5936297 10.1097/00005053-196602000-00001

[140] Nakaya M, Ohmori K. Psychosis induced by spiritual practice and resolution of pre-morbid inner conflicts. German Journal of Psychiatry. 2010;13:161–3.

[141] VAN Nuys D. Meditation, attention, and hypnotic susceptibility: A correlation study. International Journal of Clinical & Experimental Hypnosis. 1973;21:59–69.

[142] Boorstein S. Clinical aspects of meditation In: Scotton B, Chinen A, Battista J, editors. Textbook of Transpersonal Psychiatry and Psychology. New York: Basic Books; 1996.

[143] Walsh R. Initial meditative experiences: Part I. Journal of Transpersonal Psychology. 1977;9:151–92.

[144] Walsh R. Initial meditative experiences: Part II. Journal of Transpersonal Psychology. 1978;10:1–28.

[145] Shonin E VGW, Griffiths MD. Cognitive behavioral therapy (CBT) and meditation awareness training (MAT) for the treatment of co-occurring schizophrenia with pathological gambling: a case study. Int J Ment Health Addict. 2014; 12:181–96

[146] Kerr CE, Josyula K, Littenberg R. Developing an observing attitude: an analysis of meditation diaries in an MBSR clinical trial. Clin Psychol Psychother. 2011;18(1):80–93. PubMed Central PMCID: PMCPMC3032385. doi: 10.1002/cpp.700 21226129 10.1002/cpp.700 PMC3032385

[147] Shapiro DH Jr. Overview: clinical and physiological comparison of meditation with other self-control strategies. Am J Psychiatry. 1982;139(3):267–74. doi: 10.1176/ajp.139.3.267 7036760 10.1176/ajp.139.3.267

[148] Perez-de-Albeniz A, Holmes J. Meditation: Concepts, effects and uses in therapy. International Journal of Psychotherapy. 2000;5:49–58.

[149] Dobkin P, Irving J, Amar S. For Whom May Participation in a Mindfulness-Based Stress Reduction Program be Contraindicated? Mindfulness. 2012;3:44–50.

[150] Waelde L. Dissociation and meditation. Journal of Trauma & Dissociation. 2004;5(2):147–62.

[151] Craven JL. Meditation and psychotherapy. Can J Psychiatry. 1989;34(7):648–53. 2680046 10.1177/070674378903400705

[152] Fenwick P. Can we still recommend meditation? Br Med J (Clin Res Ed). 1983;287(6403):1401. PubMed Central PMCID: PMCPMC1549624. 10.1136/bmj.287.6403.1401 PMC1549624 6416433

[153] Shonin E, Van Gordon W, Griffiths M. Are there risks associated with using mindfulness in the treatment of psychopathology? Clinical Practice,. 2014; 11: 389–92.

[154] Hanley A, Abell N, Osborn D, Roehrig A, Canto A. Mind the Gaps: Are Conclusions About Mindfulness Entirely Conclusive? Journal of Counseling & Development. 2016;94:103–13.

[155] Lukoff D, Lu FG, Turner R. Cultural considerations in the assessment and treatment of religious and spiritual problems. The Psychiatric clinics of North America. 1995;18(3):467–85. 8545262

[156] Wildman W. Religious and spiritual experiences. Cambridge: Cambridge University Press; 2011.

[157] Lindahl JR, Chilcott T. Religious Experiences, Transformative Paths, And Religious Goals. Religion. 2011;41(1):79–83.

[158] Luhrmann T, Padmavati R, Tharoor H, Osei A. Hearing Voices in Different Cultures: A Social Kindling Hypothesis. Topics in Cognitive Science. 2015;7(4):646–63. doi: 10.1111/tops.12158 26349837 10.1111/tops.12158

[159] Lutz A, Slagter HA, Dunne JD, Davidson RJ. Attention regulation and monitoring in meditation. Trends in cognitive sciences. 2008;12(4):163–9. Epub 2008/03/11. PubMed Central PMCID: PMC2693206. doi: 10.1016/j.tics.2008.01.005 18329323 10.1016/j.tics.2008.01.005 PMC2693206

[160] Kabat-Zinn J. Full catastrophe living: Using the wisdom of your body and mind to face stress, pain and illness New York: Delacorte Press; 1990.

[161] Miller JJ, Fletcher K, Kabat-Zinn J. Three-year follow-up and clinical implications of a mindfulness meditation-based stress reduction intervention in the treatment of anxiety disorders. Gen Hosp Psychiatry. 1995;17(3):192–200. 7649463 10.1016/0163-8343(95)00025-m

[162] Carlson LE, Tamagawa R, Stephen J, Drysdale E, Zhong L, Speca M. Randomized-controlled trial of mindfulness-based cancer recovery versus supportive expressive group therapy among distressed breast cancer survivors (MINDSET): long-term follow-up results. Psychooncology. 2016;25(7):750–9. doi: 10.1002/pon.4150 27193737 10.1002/pon.4150

[163] Shimokawa K, Lambert MJ, Smart DW. Enhancing treatment outcome of patients at risk of treatment failure: meta-analytic and mega-analytic review of a psychotherapy quality assurance system. Journal of consulting and clinical psychology. 2010;78(3):298–311. doi: 10.1037/a0019247 20515206 10.1037/a0019247

[164] Eisendrath SJ, Gillung E, Delucchi KL, Segal ZV, Nelson JC, McInnes LA, et al A Randomized Controlled Trial of Mindfulness-Based Cognitive Therapy for Treatment-Resistant Depression. Psychother Psychosom. 2016;85(2):99–110. PubMed Central PMCID: PMCPMC4756643. doi: 10.1159/000442260 26808973 10.1159/000442260 PMC4756643

[165] Strauss C, Cavanagh K, Oliver A, Pettman D. Mindfulness-based interventions for people diagnosed with a current episode of an anxiety or depressive disorder: a meta-analysis of randomised controlled trials. PLoS One. 2014;9(4):e96110 PubMed Central PMCID: PMC3999148. doi: 10.1371/journal.pone.0096110 24763812 10.1371/journal.pone.0096110 PMC3999148

[166] Folette V, Palm K, Pearson A. Mindfulness and trauma: implications for treatment. Journal of Rational-Emotive & Cognitive-Behavior Therapy. 2006;24:45–61.

[167] Olano HA, Kachan D, Tannenbaum SL, Mehta A, Annane D, Lee DJ. Engagement in mindfulness practices by U.S. adults: sociodemographic barriers. Journal of alternative and complementary medicine. 2015;21(2):100–2. PubMed Central PMCID: PMCPMC4326023. doi: 10.1089/acm.2014.0269 25685958 10.1089/acm.2014.0269 PMC4326023

[168] Gregory P. Describing the elephant: Buddhism in America. Religion and American Culture 2001;11(2):233–63.

[169] Hickey WS. Two Buddhisms, Three Buddhisms, and Racism. Journal of Global Buddhism. 2010;11:1–25.

[170] Census Bureau US. Educational Attainment in the United States: 2014—Detailed Tables. http://wwwcensusgov/hhes/socdemo/education/data/cps/2014/tableshtml. 2014.

[171] Kilpatrick DG, Resnick HS, Milanak ME, Miller MW, Keyes KM, Friedman MJ. National estimates of exposure to traumatic events and PTSD prevalence using DSM-IV and DSM-5 criteria. J Trauma Stress. 2013;26(5):537–47. PubMed Central PMCID: PMCPMC4096796. doi: 10.1002/jts.21848 24151000 10.1002/jts.21848 PMC4096796

[172] Kessler RC, Berglund P, Demler O, Jin R, Merikangas KR, Walters EE. Lifetime prevalence and age-of-onset distributions of DSM-IV disorders in the National Comorbidity Survey Replication. Arch Gen Psychiatry. 2005;62(6):593–602. doi: 10.1001/archpsyc.62.6.593 15939837 10.1001/archpsyc.62.6.593

[173] Brattstrom O, Eriksson M, Larsson E, Oldner A. Socio-economic status and co-morbidity as risk factors for trauma. Eur J Epidemiol. 2015;30(2):151–7. doi: 10.1007/s10654-014-9969-1 25377535 10.1007/s10654-014-9969-1

[174] Barnett K, Mercer SW, Norbury M, Watt G, Wyke S, Guthrie B. Epidemiology of multimorbidity and implications for health care, research, and medical education: a cross-sectional study. Lancet. 2012;380(9836):37–43. doi: 10.1016/S0140-6736(12)60240-2 22579043 10.1016/S0140-6736(12)60240-2

[175] Sierra M, Berrios GE. The phenomenological stability of depersonalization: comparing the old with the new. The Journal of nervous and mental disease. 2001;189(9):629–36. 11580008 10.1097/00005053-200109000-00010

[176] Britton WB. NCCAM/NIH K23 AT006328-01A1 "Dismantling Mindfulness". Further details for this registered phase 3 clinical trial can be found at www.clinicaltrials.gov (NCT# 01831362) 2011.

