# Supplementary material for: The varieties of contemplative experience: A mixed-methods study of meditation-related challenges in Western Buddhists
Source: PLoS One. 2017 May 24;12(5):e0176239. doi: 10.1371/journal.pone.0176239 (PMC5443484; doi:10.1371/journal.pone.0176239)
Supplement: S1 File — (PDF) [file pone.0176239.s001.pdf]

S1 File. Practitioner Interview Questions

Lindahl, J., Fisher, N., Cooper, D., Rosen, R., and Britton, W.

Meditation history

1. Please provide a brief chronological overview of your meditation practice history, including: when you began meditating; why you began meditating; lineages, teachers, or centers with which you have been affiliated; and meditation type and practice amount at different periods of time (daily practice and retreat).

Phenomenology

1. What kinds of experiences have you had that you associate with meditation?
2. Are there any unexpected, challenging, or difficult experiences that you associate with the practice of meditation?
3. How did these experiences impact your life?

Interpretations, causes, and influencing factors

1. How did you interpret your experiences?
2. How did other people interpret your experiences? What interpretive frameworks were offered to you?
3. Why do you associate these experiences with meditation?
4. Are there any other factors that you think may have influenced the nature or course of your meditation experiences?

Remedies

1. How did you and others respond to these experiences?
2. What kinds of responses or management strategies have you found particularly helpful?
3. What kinds of responses or management strategies have you found particularly unhelpful?
