# Supplementary material for: The varieties of contemplative experience: A mixed-methods study of meditation-related challenges in Western Buddhists
Source: PLoS One. 2017 May 24;12(5):e0176239. doi: 10.1371/journal.pone.0176239 (PMC5443484; doi:10.1371/journal.pone.0176239)
Supplement: S2 File — (PDF) [file pone.0176239.s002.pdf]

S2 File. Expert Interview Questions.

Lindahl, J., Fisher, N., Cooper, D., Rosen, R., and Britton, W.

Knowledge base

1. Please describe your formal training and education as a meditation teacher and/or clinician, including: meditation practice history; training as a meditation teacher; clinical, psychological or medical training; years of experience; and populations with which you have worked.

Phenomenology

1. What kinds of experiences are associated with meditation practice?
2. Are there any unexpected, challenging, or difficult experiences that meditators have reported to you?
3. How do these experiences impact a meditator's life?

Interpretations, causes, and influencing factors

1. How do you interpret these experiences?
2. Why do you associate these experiences with meditation?
3. Are there any other factors that influence the nature or course of a meditator's experience?

Remedies

1. What do you recommend as ways for meditators to work with these experiences?
2. What kinds of responses or management strategies are particularly helpful?
3. What kinds of responses or management strategies are particularly unhelpful?
