# Supplementary material for: The varieties of contemplative experience: A mixed-methods study of meditation-related challenges in Western Buddhists
Source: PLoS One. 2017 May 24;12(5):e0176239. doi: 10.1371/journal.pone.0176239 (PMC5443484; doi:10.1371/journal.pone.0176239)
Supplement: S4 File — (PDF) [file pone.0176239.s004.pdf]

S4 File. Phenomenology Codebook. Version 1.

Lindahl, J., Fisher, N., Cooper, D., Rosen, R., and Britton, W.

**Affective Domain** (13 Categories)	**Description**	**Inclusion Criteria**	**Exclusion Criteria**
Affective Flattening, Emotional Detachment, or Alexithymia	A narrowed or diminished affective range, a lack of affective charge, and/or an inability to identify/distinguish emotions.	Descriptions of affective flattening indicate a narrowed affective range (the opposite of *Affective Lability*). Affective flattening may also reflect a lack of affect arising and/or the absence of a typical affective response to an experience that usually evokes one. Emotional detachment may be expressed as a disidentification with the emotional charge of experiences or circumstances, having emotions but not feeling them, and/or having emotions only as body sensations without a sense of affect. Descriptions of alexithymia indicate an inability to identify, understand, process, describe or distinguish emotions, or to distinguish between the somatic dimensions of emotions and other bodily sensations. Double-code with *Mental Stillness* when affective flattening or emotional detachment is described as also having a cognitive dimension, for instance in relation to descriptions of "equanimity" associated with progress in meditation. Double-code with *Meta-Cognition* when emotional detachment is associated with cognitive distancing.	Emotional detachment, flattening, or alexithymia that arises due to causal circumstances other than meditation practice. Distinguish from *Depression*, which is characterized by low and negative mood rather than an absence of affect or a diminished affective range. Differentiate from (or double-code with) *Anhedonia* (lack of interest or motivation), which is a *Conative* change.
Affective Lability	Rapid shifts in mood, mood swings, a increased range of emotions, or strong, unwarranted reactions to situations.	Being emotionally reactive or triggered, or strong shifts in mood. This may manifest as strong reactions to situations that would not have previously elicited such a reaction, or a greater range of both positive and negative emotions than ordinarily experienced. May be double-coded with *Agitation or Irritability*, or *Rage, Anger, or Aggression*, or conversely with *Positive Affect*.	Affective lability that is mentioned prior to meditation experience or that can be attributed to other causes. For unusually strong or unwarranted reactions to situations arising beyond the context of meditation, do not code instances in which meditation-related changes are not playing an influencing role. For moods that are primarily ruminative or depressive in nature, code as *Depression*.
Agitation or Irritability	An agitated or irritable mood, possibly accompanied by restlessness, distractibility or uneasiness.	Agitation and irritability are types of affective reactivity and may include descriptions of restlessness, distractibility, or uneasiness. Agitation and restlessness described with somatic language would still be coded here if an affective dimension is mentioned, and would be double-coded with *Somatic Energy* or other Somatic domain categories in cases of intense psychomotor agitation. Other common double-codes include *Increased Cognitive Processing*, *Perceptual Hypersensitivity*, *Affective Lability*, *Rage*, or *Fear or Anxiety.*	Excitation or a restless mind not attributed to meditation practice. Distinguish from *Rage, Anger, or Aggression,* which is higher in intensity and degree and more likely to be associated with behavioral change, though these may be coded in proximity if agitation and irritability develop into anger, for instance.
Change in Doubt, Faith, Trust, or Commitment	Changes (increase or decrease) in doubt, faith, trust or commitment in relation to religious doctrines, practices, goals, community or in relation to oneself in any dimension of life, such as self-confidence.	May include increase or decrease of faith with respect to religious matters, such as particular teachings or worldviews. Also includes doubt in one's ability to attain enlightenment or to be a successful meditator, or, conversely, descriptions of self-assurance and self-confidence in practice-related or other domains. May be double-coded with *Change in Worldview*, *Change in Relationship to Meditation Community*, or categories in the Conative domain, such as *Change in Motivation or Goal*.	Changes in doubt, faith, trust or commitment prior to meditation, or regarding issues unrelated to meditation or to one's identity as a meditation practitioner.
Crying or Laughing	Crying and laughing, and associated vocalizations.	Crying or laughing--for any reason or none, associated with emotional content or not, and either during formal meditation or not--that is causally attributed to meditation. Include descriptions of associated vocalizations such wailing, moaning or others. May be double-coded with *Affective Lability*, *Depression*, or *Positive Affect*.	Crying or laughing that is not attributed (in whole or in part) to meditation practice. For instance, crying or laughing due to an environmental stimulus or social situation that is not impacted in some way by the effects of meditation.
Depression, Dysphoria, or Grief	Low, depressed, or sad moods, usually coupled with physical and behavioral manifestations that may or may not affect normal functioning.	Depression includes feelings of intense sadness, emptiness, hopelessness, helplessness, guilt, or unworthiness. Dysphoria includes feelings of unease or dissatisfaction with life. Grief includes feelings of sorrow or longing associated with loss. May also be characterized by a problems concentrating on the task at hand, forgetfulness, insomnia, loss of appetite, and/or general loss of energy, drive, or motivation. Thus, where relevant, double-code with *Change in Executive Functioning*, *Self-Conscious Emotions*, *Anhedonia*, *Appetitive Changes* or *Sleep Changes*. Grief in particular may be double-coded with *Change in Relationship to Meditation Community*, *Change in Doubt, Faith, Trust, or Commitment* or changes in the *Conative Domain*.	Feelings of lowered mood without explicit connection to meditation practice. Loss of meaning or doubt are coded under *Change in Doubt*. Differentiate from (or double-code with) *Anhedonia* (lack of interest or motivation), which is a *Conative* change.
Empathic or Affiliative Changes	Increased or decreased empathic connection to other people or to environmental stimuli.	Empathic or affiliative changes attributed to meditation practice. Descriptions of increased empathic connection may include references to social emotions (love, attachment, union), valuing social communities or interpersonal relationships and, at the more extreme end, affective sensitivity and contagion (being impacted by or taking on the feelings of others). Descriptions of decreased empathic connection may include references to indifference about or increased aversion to social situations or relationships, in which case consider double-coding with *Affective Flattening*. May be double-coded with categories in the *Social Domain* (if behavioral changes are also reported) and/or *Change in Self-Other Boundaries* (when the change in boundaries also results in affective contagion).	Empathic or affiliative changes not attributed to or exaggerated by meditation practice. Affective contagion (reports of taking on the emotions of others) may need to be differentiated from or double-coded with both *Perceptual Hypersensitivity* as well as *Change in Self-Other Boundaries*, a category in the *Sense of Self Domain*.
Fear, Anxiety, Panic, or Paranoia	Feelings of fright or distress--with or without an external referent--and their corresponding physiological and behavior responses.	Some feeling of fright or distress--ranging from anxiety to fear, terror, panic, or paranoia--usually stimulated by a phenomenon that the subject is unused to, whether an external stimulus or an dimension of their meditation experience. Fear should be coded when it is reported as a response to other meditation-related changes in this codebook. However, fear or terror may also appear as a strong feeling on its own, without known or specified content or cause. Also includes intense negative emotions such as panic or paranoia and their behavioral changes that emerge from unpleasant experiences attributed wholly or in part as effects of meditation. Consider double-coding with *Delusional, Irrational, or Paranormal Beliefs* when paranoia influences thought content.	Fear, anxiety, panic, or paranoia prior to meditation-related experiences, arising outside of the context of meditation, or arising due to another identifiable cause that does not become amplified or exaggerated by meditation. General references to being afraid of/about something not accompanied by a clear affective phenomenology should not be included.
Positive Affect	A state of positive or elevated mood or energy level, ranging on a continuum from low to high arousal.	Positive affect attributed to meditation practice. Possible descriptions of positive feelings ranging from low to high levels of arousal include: peace, joy, love, gratitude, happiness, awe, wonder, excitation, enthusiasm, effusiveness, bliss, euphoria, ecstasy, rapture, grandeur, grandiosity, mania, or others. At the high arousal level, particularly with mania, positive affect may be accompanied by intense productivity or insight, in which case a double-code with *Change in Worldview* may be warranted. Mania may also be reported in conjunction with delusions of grandeur (or other delusions), or impairment in rational thinking such that one acts with overconfidence or disregard for one's safety, finances, etc., in which cases double-coding with *Increased Cognitive Processing* or *Delusional, Irrational, or Paranormal Beliefs* may be warranted.	Positive affect stimulated by external circumstances without clear association with meditative practice. General references to an experience being positive that don't clearly reference an elevated mood or other criteria denoting an affective change.
Rage, Anger, or Aggression	Feelings of intense displeasure or a retaliatory response, often caused by some adverse stimulus provoking an uncomfortable emotion.	Extreme feelings of displeasure, retaliation, anger or aggression, either in reaction to a stimulus or in the absence of a known specific stimulus. Rage, anger, and aggression may arise on their own as primary phenomenology, or they may be in response to meditation-related experiences or how such experiences were managed. A double-code with *Affective Lability* is warranted when the response is stronger than it would be under typical conditions. May also be double-coded with or coded in proximity to *Agitation or Irritability.* May lead to or correspond with various changes in the *Social Domain*.	Feelings of anger arising outside of a meditation-related context, in the past (before onset), or for reasons not causally linked to meditation. Differentiate *Agitation or Irritability*, which is marked primarily by reactivity, from *Rage, Anger or Aggression*, which is higher in intensity and degree and has different behavioral manifestations.

Re-experiencing of Traumatic Memories or Affect Without Recollection	Either a recollection of some past traumatic event in the subject's life that may or may not have been repressed, and which is generally associated with strong emotions, or the upwelling of strong emotions without any corresponding memory, content, thought or other identifiable stimulus.	Re-experiencing of traumatic memories or traumatic flashbacks include references to the recollection of some past traumatic event in the subject's life. These can also be coupled with the explicit mention of its relation to powerful emotional content such as grief, terror, or shame, in which case double-coding for those may also be warranted. Affect without recollection includes references to an experience of an unexpected, or sudden onset or upwelling of emotions without an identifiable stimulus or typical causal factor, such as a memory, a thought, or an evocative external circumstance. When re-experiencing is associated with specific locations in the body, double-code for *(Release of) Pressure, Tension* or *Somatic Energy,* accordingly.	Reference to some past event in the subject's life but without mention of it being emotionally intense or traumatic. Reference to emotions or affective response which have clear causal relationship to thoughts, memories, or external circumstances and are not unexpected. Re-experiencing of trauma or emotion not attributed to meditation practices and/or arising in non-practice contexts would also be excluded.
Self-Conscious Emotions	Emotions relating to one's sense of self and identity, as well as the awareness of reactions of others to oneself, whether real or imagined.	Include descriptions of notable increases or decreases in self-conscious emotions (guilt, shame, embarrassment, envy or pride) caused or intensified by meditation practice. Code self-conscious emotions arising as primary phenomenology, as responses to other specific meditation-related experiences, or as responses to how those experiences were responded to. May be double-coded with categories in the *Social Domain* when impacting the practitioner's relationship to social, occupational, or meditation communities.	Self-conscious emotions arising prior to meditation or in a context in no way related to meditation-related experiences should not be coded. Changes related to self-confidence or self-esteem should be coded under *Change in Doubt, Faith, Trust, or Commitment.*
Suicidality	Affect-driven ideation concerning wanting to die, not wanting to continue with life, wishing to no longer being alive, thinking about taking one's own life, or thinking about or making specific plans for taking one's own life.	Includes thoughts of contemplating the ending of one's own life, whether they arise briefly and spontaneously or are a consequence of other affective states. Passive ideation includes wanting to die, thinking about no longer wanting to be alive or taking one's own life. Active ideation includes making specific plans for taking one's own life or making an attempt at taking one's own life. Suicidal ideation can be associated with another affective experience, or conversely may arise seemingly out of nowhere. In reports of suicidal ideation that are described as unexpected and beyond one's control, consider double-coding with changes in the *Sense of Self Domain*, such as *Loss of Sense of Agency* or *Loss of Sense of Ownership*.	Suicidality that is caused by reasons unrelated (temporally or symptomatically) to meditation, such as family trauma or death of a loved one.
**Cognitive Domain** (10 Categories)	**Description**	**Inclusion Criteria**	**Exclusion Criteria**
Change in Executive Functioning	Either an inability to perform cognitive functions of decision making, concentration, and memory that the person used to be able to perform, or an enhanced ability in these domains of executive functioning.	A noticeable increase or decrease in any capacity to think, make decisions, memory recall, or the performance of other cognitive tasks. Such changes may have been noted by the subject themselves or by another observer (such as family, friends, coworkers, etc.). Diminished capacities in executive functioning also co-occur with extreme forms of *Mental Stillness*. When the person is prevented from normal functioning, work, or the ability to relate to others, double-code with *Social* or *Occupational Impairment*.	Any symptomology related to emotions, such as *Depression,* would not belong in this category, unless it also affected cognitive abilities, in which case double-coding would be appropriate. Disambiguate from *Disintegration of Conceptual Meaning Structures*, otherwise known as cognitive defusion.
Change in Worldview	A shift in ways of thinking about the nature of self or reality, including a change in understanding or confusion about the nature of self or reality.	A change in worldview as a result of contemplative practice, for instance a change in relationship to or understanding of Buddhist teachings, doctrines, or views. Change in worldview may arise during a content-driven practice, a content-minimal practice, or following a practice session so long as the influence of prior meditation is apparent. May have many associated effects, including changes in the Conative domain such as *Change in Effort* or *Change in Motivation or Goal*, changes in the *Sense of Self Domain*, or *Scrupulosity*. May also be double-coded with *Change in Relationship to Meditation Community.*	Change in worldview due to learning or an interpersonal influence not associated with meditation or the associated teachings that are implemented in practice. Reports of beliefs held independent of meditation-related changes or interpretations of prior phenomenology should not be coded. Ordinary confusion (not pertaining to views of self or reality) should be coded under *Change in Executive Functioning*. Causal attributions made to the effect of worldviews on meditation-experiences would be coded as *Influencing Factors*.
Clarity	Reports of clarity or lucidity as a mental state, quality of attention, or quality of consciousness, in which there is a heightened cognition of relevant stimuli and a diminished interference from non-relevant stimuli.	Primarily described as a pervasive quality of mind, consciousness, or awareness or attention. Often associated with a figure-ground shift such that the practitioner attends more to the field of awareness rather than or in addition to discrete perceptual objects. May also be thought of as the opposite of the fogginess or dullness of *Mental Stillness*, in the sense of a clarity of cognitive or perceptual processing. May be reported in conjunction with *Increased Cognitive Processing*, *Perceptual Hypersensitivity* or other changes in the *Perceptual Domain*, in which case double-code accordingly.	The perception of an environmental clarity (such as space or air) that is not attributed to a change in the quality of awareness should not be coded. Brightening of the visual field should be primarily coded under *Visual Lights*. Other forms of *Perceptual Hypersensitivity* may coarise with *Clarity*; if the change is described exclusively in perceptual terms with no cognitive dimension, code under the relevant Perceptual category instead. Some reports of expansiveness/spaciousness may be more appropriately coded under *Change in Self-Other or Self-World Boundaries*.
Delusional, Irrational, or Paranormal Beliefs	Holding with conviction and being influenced by one or more beliefs despite evidence to the contrary. Ascriptions of significance or meaning that are later disregarded or that might seem unusual or concerning to members of the practitioner's broader culture or particular subculture. Attributions of paranormal agency, origin, or explanation for cognitive experiences.	Reports of distortions of grandiosity, paranoia, or mania that lead to some form of misapprehending or no longer participating in consensual reality. Beliefs that were reported as being strongly held as true despite feedback from others to the contrary or based upon rational or empirical evidence. Or beliefs that were described by the subject in retrospect as delusional or irrational in nature. Subjects may or may not have "insight" into the delusional nature of their beliefs at the time they are describing, and subsequent attributions or reflections on their prior experience should be taken into account. Also include here beliefs that are reported as seeming unusual or concerning to an authority in their culture or subculture (such as a meditation teacher), a friend or family member, or a mental health professional. Includes beliefs that affect the structuring of perceptual, cognitive, affective, or interpersonal experiences. Includes magical thinking and paranormal beliefs such as communication with agents, paranormal powers, access to forms of knowledge that would not be verifiable by a second-person source or experiences of hypersalience (anomalous gnostic events). Paranormal beliefs may also be double-coded with *Change in Worldview*, especially in cases where paranormal experiences are valued as indicating a contact with a deeper, truer, and/or usually hidden reality. When non-consensual changes in perception co-occur with changes in belief, double-code for *Hallucinations, Visions or Illusions*.	Statements that appear far-fetched but are not demonstrated to have been viewed as delusional at the time or in retrospect by the practitioner, *and* were not be deemed delusional, irrational, or paranormal by a member of the practitioner's subculture or broader culture (including Western psychiatry). Associated beliefs must have clear phenomenological referent or intersect with phenomenological reports. Casual post-hoc descriptions of prior events as "delusional" or "irrational" should not be coded unless the events themselves indicate clear cognitive changes meeting inclusion criteria. Delusions that manifest with a percept-like experience in the absence of perceptual input would be coded as *Hallucinations, Visions, or Illusions*. Should be disambiguated from the manic dimensions of *Positive Affect*, which may arise without delusional cognitive content.
Disintegration of Conceptual Meaning Structures	Percepts arise but are processed without their associated conceptual meaning, resulting in an inability to form conceptual representations of the perceptual world.	Difficulty in navigating situations because of an inability to grasp previously familiar and basic concepts associated with percepts, such as running a red light because the color red loses its meaning as signifier of stopping, or the shape "5" no longer being recognized as representing the number five. Word-finding problems associated with identifying the name or function perceptual objects would be coded here. May be described as evolving from a more mild form of "cognitive defusion" that would be better coded under *Meta-Cognition*. May also arise in conjunction with changes in the *Sense of Self Domain*.	This may be intentionally cultivated through *Meta-Cognition* and other forms of "cognitive defusion," but disambiguate from meta-cognitive events in which conceptual processing of percepts remains intact. Other forms of confusion or impairment in functioning that are unrelated to concepts, as well as word-finding problems due to problems with memory, would be better coded under *Change in Executive Functioning*.
Increased Cognitive Processing	Primarily a cognitive change in thought amount or speed of cognitive processing, though the increase in processing often coincides with a decrease in sensory gating that leads to the impression of taking in or processing more perceptual information than usual.	The perception of thoughts and sensations as occurring more rapidly than normally experienced, attributed to a change in a state of mind or cognitive processing. Includes statements mentioning a racing mind or increase in cognitive processing speed and reports of "stimulus overload." May also lead to or be associated with overwhelm and *Anxiety* in the *Affective Domain*. Racing thoughts associated with reports of mania--in which someone is entirely preoccupied by the thought process and may lose track of time--may also be warrant double-coding with *Positive Affect* and/or *Delusional, Irrational, or Paranormal Beliefs*.	Do not include increased thought amount not caused by meditation or meditation-related symptoms such as fear or anxiety. Do not include rapidity of stimuli attributed to changes in the external world and not in the cognitive processing of the practitioner. Differentiate from an increased noticing of or *Meta-Cognition* of thoughts. Perceptual changes--such as reality appearing to be composed of rapidly moving vibrations, particles or pixels, or other descriptions indicating increased processing of percepts--should primarily be coded as *Perceptual Hypersensitivity* or *Dissolution of Objects* unless they are accompanied by a clear cognitive phenomenology.

Mental Stillness	An state in which there are few identifiable thoughts, a perceived absence of thought, or a poor awareness about the thinking process in general.	Mention of an absence of thought, whether positively or negatively valenced, intentionally sought or involuntarily experienced. Includes both target states of "calm abiding," or "mental quiescence" associated with concentration practice, as well as unexpected, prolonged, or undesirable states of "spacing out without mind wandering," "fogginess," or a general obscuration of cognitive processes. Lack of access to thought (inability to generate thought) is an extreme and often involuntary version of *Mental Stillness*.	In cases of mental fog, attempt to differentiate absence or thought from an a *Change in Executive Functioning*. Corresponding flattening of emotions or a lack of affect should be coded under *Affective Flattening*. Expected cycles of drowsiness or dullness should not be coded here.
Meta-Cognition	Meta-cognition, or meta-awareness, refers to an explicit knowledge of the content of thoughts or the thinking process. Meta-cognition can also entail a higher-order cognition of processes in other domains of experience, such affective, perceptual, somatic or sense of self.	Sustained meta-cognition or meta-awareness resulting from the practice of meditation. Includes reports of a "distancing" from thoughts, seeing thoughts as "just thoughts," or as seeing thoughts as transient events in the mind. Also includes the explicit reference of a "monitoring" awareness witnessing transient somatic, affective, or perceptual events. In cases where the transience of phenomena is described as an understanding of Buddhist views such as "impermanence," or "arising and passing away," consider double-coding with *Change in Worldview*. Reports of resting in a "witness consciousness" would also be coded here, and may also entail double-coding with changes in the *Sense of Self Domain*.	A brief moment of meta-cognition, or meta-cognition not related to meditation or to particular views and values associated with meditation. Prolonged and intense forms of meta-cognition may develop into a *Loss of Sense of Ownership* over thoughts, emotions, or body sensations or a *Loss of Agency* over actions, at which point those codes would be more appropriate. Some forms of distancing from thoughts and the content of thoughts in which a cognitive event is no longer processed with associated meaning or significance may be better coded (or double-coded) as *Disintegration of Conceptual Meaning Structure*s.
Scrupulosity	Obsessive thinking, specifically about moral or religious issues and behaviors.	A change in self-perception or behavior in relation to religious teachings or moral values associated with contemplative practices. Also includes the exacerbation of existing dispositional tendency towards obsession over moral or religious issues when influenced by meditation or being in a meditation context. May often be precipitated by or result in *Change in Worldview* or *Change in Effort,* and may also be co-occurring with behavioral changes in the *Social Domain*.	Changes in self-perception and behavior coming from other religious contexts (such as a practitioner's former religious tradition), unless those tendencies are exaggerated by meditation. Only beliefs and associated behaviors related to morality or religious codes of conduct should be coded here. Disambiguate from *Delusional, Irrational, or Paranormal Beliefs*, *Change in Doubt, Faith, Trust or Commitment*, and *Change in Worldview*.
Vivid Imagery	An experience of intense, vivid and/or clear thoughts or mental images that arise involuntarily, or a report of an increased ability to visualize.	Intense and potentially disconcerting thoughts that arise involuntarily and which are accompanied by an increased clarity or vividness. Vivid imagery or thought intrusions may also be accompanied by strong emotions such as *Fear* or bliss (*Positive Affect*).	Vivid fantastical images that are perceived as if external objects would be coded under *Hallucinations*. Vivid imagery that is associated with traumatic memories should primarily be coded as *Re-experiencing of Traumatic Memories.* Disambiguate from *Clarity*, which may be a coinciding quality of consciousness, but mentioned independent of vivid imagery. Disambiguate from *Hallucinations, Visions, or Illusions* which are percept-like changes in the *Perceptual Domain*.
**Conative Domain** (3 Categories)	**Description**	**Inclusion Criteria**	**Exclusion Criteria**
Anhedonia or Avolition	Anhedonia is the inability to experience pleasure in activities previously found pleasurable. Avolition is the lack of drive or motivation to pursue goals previously valued as meaningful.	Anhedonia and avolition are involuntary reductions in pleasure and drive. Anhedonia (the inability to experience pleasure) often leads to avolition (not being motivated to act). Anhedonia may emerge from *Affective Flattening* or *Depression* and tends to manifest as a change in motivation that affects functioning. Thus double-coding with *Social Impairment* or *Occupational Impairment* may also be appropriate. When avolition is related to changes in interests or values due to other phenomenology, double-coding (or coding in proximity to) *Change in Worldview* or *Change in Doubt, Faith, Trust, or Commitment* may be appropriate.	Disambiguate from contentment, which is a low arousal *Positive Affect*, and from equanimity, which is better coded under *Emotional Detachment* or *Mental Stillness* accordingly. Anhedonia should not be coded in the case of simple voluntary changes in what the practitioner takes interest in, nor is it related to maturational changes that result in corresponding change in interests; rather, anhedonia and avolition are pervasive or comprehensive reductions in pleasure and interest that leads to a change in behavior. Simple changes in interest pertinent to the practice of meditation might be better coded under *Change in Effort* or *Change in Motivation or Goal*.
Change in Effort or Striving	The degree or intensity of attempts at pursuing something valued-as-good or as a means to a valued end. Effort may be mental, physical, or emotional.	An increase in the effort, striving, trying, diligence or active pursuit of meditation or a meditation-related goal. Includes reports of zeal, enthusiasm, being "hardcore", becoming addicted to meditation, or wanting to meditate for an increasingly long duration or increased frequency. Conversely, a diminishment in effort, striving, trying, or active pursuing of meditation or a meditation-related goal. Includes reports of a decrease in duration of practice sessions or practice frequency if they have an associated conative phenomenology. Reports of involuntary decreases in effort might also co-occur with *Anhedonia*.	Increase or decrease in effort, striving, drives, or the pursuit of goals not related to or intersecting with meditation practice. Change in practice amounts for circumstances that are unclear or without a conative dimension should not be coded. Various factors associated with practice effort may also be coded under *Change in Motivation or Goal* or *Change in Worldview*. Striving dispositions or amount of effort applied to meditation that are described as risk factors for other meditation-related experiences should be coded as *Influencing Factors*.
Change in Motivation or Goal	The reasons, drives, and needs behind a practitioner's actions, which influence or determine their behavior, as well as their expectations concerning a particular behavior.	Includes references to changes in the effect of or influence of motivating factors or goals on behavior, which may be double-coded with *Change in Effort*, as well as to references in the change of the motivating factors or goals themselves, which may be double-coded with *Change in Worldview*. Includes references to the appearance of newly desired expectations about the results of practicing meditation, such as experiential states or behavioral traits. Includes changes in reasons for taking up the practice of meditation, for changing practices, and for continuing to practice meditation in the face of challenging or difficult experiences.	References made to motivations, goals, or expectations that do not influence or stem from meditation-related behaviors should not be coded. References made to other goals in life or motivating factors for other, non-meditation-related behaviors also should not be coded. References to the influence of motivations or goals on other types of meditation-related experiences or references pertaining to the alleviation of meditation-related difficulties are should be coded as *Influencing Factors.*
**Perceptual Domain** (7 Categories)	**Description**	**Inclusion Criteria**	**Exclusion Criteria**
Derealization	Surroundings are perceived as strange, unreal, or dreamlike, or perception is experienced as mediated by a fog, a lens, or some other filter that results in feeling cut off from the world.	Mentioning a feeling of unreality, strangeness, unfamiliarity or dreamlike quality of surroundings, or reports of feeling cut off or alienated from the perceptual world. Includes confusion concerning whether one is dreaming or awake. Perceptual changes include objects appearing two-dimensional, or "cartoon-like," which are often reported with corresponding affective changes about objects no longer feeling real. Derealization can thus be characterized by feelings of the world lacking emotional coloring and depth in addition to related perceptual changes, in which case double-coding with *Affective Flattening* may be warranted. Reports of strangeness or unfamiliarity co-occurring with affective flatness should also be double-coded. May also be double-coded with various other perceptual distortions as well as *Loss of Sense of Basic Self*.	Changes in sense perception without the sense of feeling dreamlike, or unreal would be coded under the appropriate change in the Perceptual domain. Senses of separation from the world may sometimes be better coded as a *Change in Self-World Boundaries* when descriptions do not include perceptual distortions. Feelings of a loss of emotional tone without associated perceptual changes or feelings of unreality should be coded as *Affective Flattening*.
Dissolution of Objects or Phenomena	The dissolving or complete disappearance of visual objects or the entire visual field.	An experience in which parts of objects, entire objects, or the entire visual field is distorted to the point of seeming composed of points of scintillating light, pixelating, dissolving, or entirely disappearing. Changes described in terms of luminosity, such as shimmering, may be double-coded with *Visual Lights*. May also follow or co-occur with *Perceptual Hypersensitivity*, especially hyperchromia, as well as *Increased Cognitive Processing*.	Differentiate from *Distortions in Time or Space*, which does not include objects. Metaphorical reports of the impermanence or arising and passing away of phenomena, influenced by Buddhist view and doctrine, should not be included here unless they involve corresponding perceptual changes of instability, flux, or dissolution. Differentiate from *Clarity*, which may involve similar metaphors of translucency applied to cognitive rather than perceptual processes.
Distortions in Time or Space	An alteration in the subjective experience of spatial boundaries or relations and/or temporal causality or sequencing.	Abnormalities in one's perception of space and/or time. May refer to one's perception of distance, scale, time, causality, or personal history. Includes references to temporal gaps or absences. Includes distortions in spatial relationships. Distortions in time and space may be double-coded with change in the *Sense of Self Domain*, especially *Loss of Sense of Basic Self*.	Discussion of time and space that do not describe distortions but ordinary experiences of time (teleology from past, present, to future) and/or space (ordinary body awareness or awareness of objects). Distortions in time/space that are substance-induced or not meditation-related. Disambiguate from *Somatosensory Changes* or *Change in Self-Other or Self-World Boundaries* that affect one's relationship to space or objects in space, as well as from *Dissolution of Objects*.

Hallucinations, Visions, or Illusions	A hallucination is an experience of a percept that is not externally stimulated, is not shared by others, and is not taken to be veridical. When a visual percept that is not shared by others is taken to be veridical, it is a vision. An illusion involves a percept that is distorted, changed, or has features added to the raw percept.	A hallucination is the experience of a percept in the absence of a corresponding sensory stimuli. Hallucinations can occur in any modality: visual, auditory, gustatory, olfactory, or proprioceptive. Hallucinations are unique to the subjectivity of the practitioner, are not a part of shared, consensual reality, and are generally understood not to be veridical. Percepts unique to the subjectivity of the practitioner, not part of consensual reality, but that *are* taken to be veridical are be better described as visions (for the visual domain, locutions for the auditory domain, etc.). Visions may be further distinguished from hallucinations by the causal origin of non-consensual percept being attributed to a non-shared agent. For example, a non-consensual percept of the Buddha may be attributed to the Buddha's agency and taken to be veridical, rather than hallucinatory. An example of an illusion, or a distortion of perception, would be micropsia or macropsia, in which objects appear larger or smaller than they would normally. Double-code with *Delusional, Irrational, or Paranormal Beliefs* when hallucinations or visions have corresponding cognitive content or impact cognitive content.	Hallucinations or illusions not reported either in the context of meditation practice or in the context of post-meditation effects. Distortions of percepts arising due to conditions other than meditation. Simple visual hallucinations that fit under the criteria for *Visual Lights* should be coded under that category. *Delusional, Irrational, or Paranormal Beliefs* or thoughts that do not have a seeming percept associated with them would not be coded here. *Somatosensory Changes* can include body-related illusions, and thus should be coded or double-coded there. Body-related hallucinations would still be coded here, unless they better fit the description of *Somatic Energy*. Strong mental imagery not experienced as a quasi-percept would be better coded as *Vivid Imagery*, unless the attribution of agency is in line with that of a vision, in which case double-coding would be most appropriate.
Perceptual Hypersensitivity	Unusual or atypical sensitivity to certain frequencies or volumes of sound (hyperacusis), to color (hyperchromia), to visual details, to light, to taste, to smell, or to embodiment.	Being extremely sensitive to sounds, light, colors, tactile sensations or other environmental stimuli. Also commonly described as difficulty tolerating everyday sounds, lights, or other sensory stimuli, in which cases double-coding with *Agitation* may be appropriate. Hypersensitivity enduring beyond or becoming apparent after a formal practice session or retreat may correspond with or lead to problems with *Integration following Retreat* and to *Social Impairment*. Increased sensitivity to visual objects or the body may lead to certain distortions, in which case this may lead to coding proximity with *Dissolution of Objects* or *Somatosensory Changes*, respectively.	Sensitivity to sound, lights, colors, smells etc. that are not produced by externally present stimuli--that is, which are *Hallucinations, Visions, or Illusions*--would be coded there. Reports of ordinary experiences of sounds, lights, colors, etc. that does not emphasize being overly stimulated or sensitive. Affective hypersensitivity should be coded primarily under *Affective Lability*. Hypersensitivity to body schema and proprioception should be coded primarily under *Somatosensory Changes*.
Somatosensory Changes	A change in proprioceptive information that affects one's perception of relative positions or dimensions of body parts or the body more generally.	Increased proprioceptive information, or increased awareness of or sensitivity to the body schema. Distortions in the body schema resulting in a change in scale of body parts or the body in general, disappearance of body boundaries (such as feeling that arms are missing), inaccurate perception of position of body parts (such as feeling that legs as being twisted when they are not), or changes in body scale (such as body parts feeling larger or smaller or dissolving). Increased proprioceptive information leading to changes in relationship to body schema may emerge from *Perceptual Hypersensitivity* of embodiment, and thus may be coded in proximity or double-coded together.	Mentioning the body in a way unrelated to one's perception of it. Statement about metaphysical disconnection/disidentification with the body, such as feeling like "no one" is in body, would be better coded in the *Sense of Self Domain*. *Change in Self-Other or Self-World Boundaries* may also lead to or result from changes in body schema that affect self-other boundaries. Feelings of being displaced from the body should be coded as *Change in Sense of Embodiment*. Bodily hallucinations (of body-related phenomena that are not actually part of the body or distortions of the body) should be coded under or double-coded with *Hallucinations, Visions, or Illusions*. Distortions or sensitivities described as involuntary surges of energy or electricity would be better coded as or double-coded with *Somatic Energy*.
Visual Lights	Experience of a light or lights in field of vision that are vivid but not the result of external stimuli.	Visual hallucinations or visions related to light or lights, which can be bright and/or colored, and can be experienced with closed or open eyes. Lights may be "discrete" circumscribed shapes or patterns or "diffuse" lights or changes in the overall brightness of the visual field. Diffuse lights or brightening of the visual field may also be correlated with the cognitive quality of *Clarity*, in which cases double-coding or coding-proximity may be warranted.	Experiences within the visual field that refer to externally present lights. Visual hallucinations in the form of recognizable shapes, objects, or scenes would be coded as *Hallucinations, Visions, or Illusions*. *Perceptual Hypersensitivity* to light or color should be coded in that category.
**Sense of Self Domain** (6 Categories)	**Description**	**Inclusion Criteria**	**Exclusion Criteria**
Change in Self-Other or Self-World Boundaries	Expansion beyond or distortions in the typical sense of where the boundaries between self and other or self and world are delineated.	Includes references to being expanded beyond one's body schema, or the sense of boundaries between self and other or self and world being dissolved. May also be described in terms of merging with, being porous or permeable to either the world or other people. Alteration of self-other boundaries includes experiencing other people's mind states or emotions, in which case double-coding with *Empathic or Affiliative Changes* may be appropriate. References to awareness as spacious, expansive, centerless, non-local, all-pervading, or non-dual with the world of experience would be coded here, as would references to a sense of unity or oneness with nature, the environment, the world, or the universe. Conversely, feeling more separated from the world or feelings as if distant from it could also be coded here. Expansiveness may have associated phenomenology from other domains, such as increases in *Clarity*, *Positive Affect,* or *Affective Lability*.	Does not include reports of cessation of consciousness or a sense of "not being there." Be careful to disambiguate with *Change in Sense of Embodiment* for reports where the sense of self is displaced to an atypical location within or in relation to the body schema. Hypersensitivity to others' emotions without reference to a change in boundaries should be coded as *Empathic or Affiliative Changes*. Hypersensitivity to the body schema without corresponding alterations in boundaries between self and other or self and world would be coded as *Somatosensory Changes.* Feeling more distant from others or from the world should be coded as *Derealization* when this is attributed to the visual sense of a filter or opaque medium, or to a loss of sense of reality.
Change in Narrative Self	A report of a change in how the practitioner conceives of himself or herself as a person. Or, a change in the content of or their perspective on their story or personal identity.	References that compare how a practitioner used to think about themselves as a person or their story that are in contrast with how they currently feel or felt after a shift associated with meditation practice. Also includes references to a complete loss of narrative identity. The narrative self is temporally extended in reference to past or future conceptions of self or personhood. Thus, changes in narrative self include the impact of different perspectives cultivated through the theory and practice of meditation on the stories practitioners tell about themselves. Such instances may benefit from double-coding with *Change in Worldview*. Stories may include descriptions of changes in how they view the motivations for their behaviors, the type of person that they think they are, or the type of person that they think they should be, in which cases double-coding with *Change in Motivation or Goal* may also be appropriate.	References to stories of self that do not appear to be emergent from meditation or changed by the theory and practice of meditation should not be coded. Statements in which a practitioner describes himself or herself only in the present moment, without implicit or explicit suggestions of a shift or change from previous conceptions of themselves. Theoretical discussion of topics related to the inclusion criteria but not explicitly connected to the practitioner's sense of himself or herself as a person or to a "story of me." Strictly doctrinal or theoretical conceptions of self or person might be better coded as *Change in Worldview* or as Influencing Factors.
Change in Sense of Embodiment	Feeling of being disembodied, located outside or at a distance from one’s body, or located in an unusual location within one's body schema.	Includes any change in where one's sense of self is located relative to the body schema. Includes references to a change in perspective of location within the body schema, such as from behind the eyes, to the middle of the head or to the heart. May also refer to a distance from the body schema, where the sense of self or perspective is located behind the body or in space, as in "out-of-body" experiences.	Distinct from reports of expansiveness associated with *Change in Self-Other or Self-World Boundaries*, as disembodiment still has a particular vantage point or location, and is not everywhere/nowhere. Changes or distortions in body image that do not reflect a change in locatedness of the sense of self would be coded in *Somatosensory Changes*.
Loss of Sense of Agency	A loss of a sense of ownership or sense of control over one's actions.	Mention of the lack of a "doer," or a sense that there is "no one" in the body who decides, controls, or executes actions, whether habitual or intentional. References to actions happening on their own or to feeling like a "puppet," an automaton, or subject to (the will of) an external force. Also includes references to disconnect from and disidentification with embodied actions such as movement, or to a sense of a being a detached witness/observer to action, but in these cases must also be suggestive of a diminished sense of or loss of agency.	*Loss of Sense of Ownership* not pertaining to actions should be coded in that category instead. Also to be distinguished from an expanded sense of self in *Change in Self-Other or Self-World Boundaries*. Care should be taken to disambiguate from or double-code with *Meta-Cognition*, through which similar changes can occur in a more transient, and less enduring manner, especially in relation to habitual actions. Discussions of energy running through the body and resulting in bodily movements should be coded as or double-coded with *Somatic Energy* and *Involuntary Movements*.

Loss of Sense of Ownership	A loss of the usual sense of owning one's thoughts, body sensations, emotions, and/or memories.	Reports that thoughts, body sensations, and/or emotions don't feel like "mine" or like they "belong to me." This may include the feeling that they belong to someone else, or they may be experienced as "impersonal." May include seeing "thoughts as just thoughts" or emotions as impersonal events in the mind or body. Thoughts may no longer become personally relevant and may lose their emotional charge or salience, in which case double-coding with *Emotional Detachment* may be warranted. Includes the experience of anomalous subjective recall--the experiencing of personal events such that they feel impersonal, or as if they happened to someone else, or to oneself but feel unusually distant in time.	Some mild forms of "seeing thoughts as thoughts" may be better coded (or double-coded) as *Meta-Cognition*. Similarly, some forms of distancing from thoughts and the content of thoughts in which a cognitive event is no longer processed with associated meaning or significance may be better coded (or double-coded) as *Disintegration of Conceptual Meaning Structures*.
Loss of Sense of Basic Self	A loss of the sense of existing, of being a self, or of having a self.	References to "not being there," to "disappearing," to "being absent," or to "not being present." These changes in sense of self are reported as happening at a very basic level of the sense of self. They may be associated with other types of changes in sense of self, whether concerning embodiment, ownership, or agency, but references should signal an alteration at this basic level of existence or being. Includes references to changes in or problems with the "unity" of various sensory, affective, and cognitive dimensions of experience.	Changes in sense of self happening at other levels should be coded accordingly. If a loss of ownership or agency is specified, code *Loss of Sense of Ownership* or *Agency*. If associated with a change in embodiment is referenced, code *Change in Sense of Embodiment* or *Change in Self-Other or Self-World Boundaries*. Unspecified references to changes in sense of self that do not seem to be altering the core sense of existence or being should be coded as *Other*.
**Social Domain** (5 Categories)	**Description**	**Inclusion Criteria**	**Exclusion Criteria**
Change in Relationship to Meditation Community	Changes in relationship with the meditation community (*Sangha*), whether increasing or decreasing degrees of affiliation with the community of teacher(s) and other practitioners.	Can include an increased sense of affiliation, belonging or commitment, or an increased sense of trust or faith in the community or teacher, in which cases double-coding with *Change in Doubt, Faith, Trust, or Commitment* may be warranted. Can also include a change in role towards increasing participation such as becoming a monk or teacher or taking on other institutional roles. Conversely, can also include a decreased sense of affiliation, belonging or commitment, a decreased sense of trust or faith in the community or teacher. This may include conflict, tension or fallout with meditation teachers or community, no longer adhering to the disciplinary standards of the community, being excluded from (or shunned by) one's community, or being banned from future retreats. Often double-coded with *Change in Worldview*. May be related to *Conative* changes, such as *Change in Motivation or Goal* in relationship to meditation community.	Social isolation or impairments that are not related to the meditation community, or *Sangha*. Increased or decreased relationships to other communities would be coded under *Increased Sociality* or *Social Impairment*, respectively. Change in relationship with meditation communities should be a direct consequence of meditation practice. Disambiguate phenomenological changes from other aspects of relationships to meditation communities that influence meditation-related experiences and which should be coded in the *Influencing Factors* codebook.
Increased Sociality	Increased extraversion, social contact, friendships or other behavioral manifestations indicating an increased valuing of social engagement.	Clear behavioral manifestations of an increased valuing of social engagement. Examples include increased extraversion, increased social contact or friendships, new social activities, or resuming social activities previously neglected. Double-coding with changes in the *Conative Domain*, especially *Change in Motivation or Goal*, may be appropriate.	Does not include rhetoric from Buddhist traditions about prosocial values such as compassion or the *bodhisattva* vow unless accompanied by behavioral changes. Increased sociality not attributed to meditation practice would also not be coded.
Integration Following Retreat or Intensive Practice	A destabilizing transition from intensive formal practice to informal practice, daily life, or life circumstances.	Includes any destabilizing transition from formal practice, daily life, or life circumstances, typically following retreat but also including the transition from any period of formal practice back into daily life. The key inclusion element is a reference of temporal proximity to a formal practice session (daily or retreat) and a reference to challenges related to this re-engagement (e.g., driving a car or interacting with people). Also includes the range of experiences that are desirable or pursued on retreat but that are inefficient, impairing, or difficulty-causing in life outside of the practice context. In the case of conflict or tension between the views and values of practice and those of everyday life, double-code with *Change in Worldview* or with categories in the *Conative Domai*n. Will often be double-coded with another category that specifies the nature of the impairment itself, such as *Social Impairment*, *Perceptual Hypersensitivity*, *Increased Cognitive Processing,* etc.	Any difficulty that arises within a retreat or within daily practice that doesn't specifically have to do with re-engagement with one's life duties or with actions in daily life. Difficulties in one's daily life that are not temporally proximate to a period of intensive practice, such as new difficulties that arise on account of prolonged and enduring meditation-related symptoms but that are not emergent in the transition from a period of intensive practice. Impairments in daily life unrelated to meditation practice should also not be coded.
Occupational Impairment	An impaired ability to perform in an occupational environment.	Describing a decreased ability to function in a normal work environment or fulfill the roles of a job.	Social impairment or personal feelings of isolation or loneliness that are unrelated to impairment in occupational functioning. If social impairment is directly causing occupational impairment, double-code with *Social Impairment*.
Social Impairment	Behaviors indicative of a change in relationship to social networks or social situations that inhibits ordinary or desired functioning or level of engagement.	Behavioral manifestations of a subjective feeling of disconnection from social networks (including friends, family, institutions, cultures, religious groups, etc.), or of a general sense of loneliness within said networks, possibly stemming from religious, cultural, emotional, or intellectual differences, or from novel experiences. This could include a decrease in social engagement or activity, a loss of friendships, a decreased ability to interact with others in ways conducive to maintaining social relationships. Should be accompanied by statements clarifying that the degree of social engagement is other than typical, optimal, or desired. Double-coding with *Affective Flattening,* or *Anhedonia or Avolition* may often be warranted.	References to social isolation that are voluntary and desired, such as periods of retreat or disassociating from unhealthy relationships, would not be coded here. Describing physical isolation but not including subjective feelings of disconnection from social networks. Subjective feelings of isolation without a behavioral manifestation should be coded under *Empathic or Affiliative Changes*, *Depression*, or *Affective Flattening.* Social isolation related to the meditation community should be coded under (or double-coded with) *Change in Relationship to Meditation Community.*
**Somatic Domain** (15 Categories)	**Description**	**Inclusion Criteria**	**Exclusion Criteria**
Appetitive or Weight Changes	Decreased or increased appetite, weight loss or gain.	Decreased or increased appetite, weight loss or gain attributed to meditation practice. Includes a general disgust with or increased attraction to food. Decreased appetite may be double-coded with *Anhedonia* or *Depression* when these co-occur.	Decreased or increased appetite, weight loss or gain attributed to a cause unrelated to meditation.
Breathing Changes	Altered respiration rates that may manifest as a temporary cessation, or speeding up or slowing down of breathing.	Breathing irregularity, either during formal meditation or not, that is attributed to meditation practice. Pre-existing breathing irregularity made worse by meditation practice. Can also include symptoms like labored breathing or a feeling of suffocating. Breathing irregularity may be valenced as positive (calming, relaxing, feeling that it is easier to breathe) or negatively valenced (distressing, feeling of suffocating, etc.)	Breathing irregularity not attributed to meditation practice. Pre-existing breathing irregularity not made worse by meditation practice. Intentionally controlling or regulating the breathing should not be coded.
Cardiac Changes	Irregular heartbeat, heart palpitations, or other significant irregularities.	Cardiac irregularity attributed to meditation practice or pre-existing cardiac irregularity made worse by meditation practice. Cardiac changes include tachycardia (unusually rapid heart beat, even at rest), brachycardia (unusually slow heart beat), and heart palpitations.	Cardiac irregularity not attributed to meditation practice; pre-existing cardiac irregularity not made worse by meditation practice.
Dizziness or Syncope	Dizziness, vertigo (feeling one is spinning or off-balance), lightheadedness (feeling one is about to faint), or syncope (a brief loss of consciousness and muscle strength, commonly called fainting, passing out or blacking out).	Dizziness, vertigo, lightheadedness, or syncope attributed to meditation practice or associated with other meditation-related symptoms such as cardiac or breathing irregularities.	Dizziness, vertigo, lightheadedness, or syncope not attributed to meditation practice. Nausea not resulting from dizziness, lightheadedness or vertigo might be more appropriately coded under or double-coded with *Gastrointestinal Distress or Nausea*.

Fatigue or Weakness	A feeling of exhaustion, fatigue or weakness (general or localized).	Feelings of exhaustion, fatigue or weakness (general or localized) attributed to meditation practice. Also includes (but not limited to) statements about chronic fatigue, which may co-occur with increased sleep need (and therefore should be double-coded with *Sleep Changes*). Fatigue may also correlated with cognitive impairments such as *Change in Executive Functioning*.	Pre-existing conditions that involve fatigue, or the development of fatigue that is attributed to a source other than meditation (such as Lyme Disease or other medical history).
Gastrointestinal Distress or Nausea	Gastrointestinal problems including (but not limited to) diarrhea, bloating, cramping, nausea and vomiting.	GI distress or nausea attributed to meditation practice or viewed as a consequence of other physiological effects caused by meditation.	GI distress attributed to diet, location, or other non-meditation causes (e.g., GI issues related to food while on retreat in India). Nausea associated with dizziness, lightheadedness or vertigo should be double-coded with *Dizziness or Syncope*.
Headaches or Head Pressure	Ache, sharp pain, or pressure in the region of the head or neck.	Headaches or head pressure attributed to or exacerbated by meditation. Headaches includes migraines or head pressure often associated with breathing. Includes any brief, prolonged, or intermittent sensations of pressure in the head as well as any associated or subsequent pain or discomfort.	Headaches or head pressure not attributed to or exacerbated by meditation practice, such as from head trauma or a sinus headache. *Pain* and *Pressure or Tension* elsewhere in the body should be coded accordingly with the general categories.
Involuntary Movements	A motor movement usually under voluntary control that occurs without a conscious decision for movement.	Involuntary movements that are attributed to meditation practice. This includes spontaneous movements such as: tics, spasms, twitching, rocking, shaking, seizing up, twisting of the torso or head, fidgeting, or others. Includes involuntary vocalizations. May be repetitive or a single involuntary movement. When occurring with reports of *Somatic Energy*, double-code or code sequentially.	Involuntary movements not attributed to meditation practice. Differentiate from *Loss of Sense of Agency*, which is a change in the sense of feeling of ownership over typical or ordinary actions; double-code in cases where it is unclear if the action was considered typical or sporadic, or where involuntary movements subsequently impacted feelings related to sense of self.
Pain	Pain is an unpleasant physical sensation, either diffuse or acute, and lasting for variable amounts of time.	Pain of any kind attributed to meditation practice beyond typical and expected postural aches and pain. Include also the relieving of pain attributed to meditation. When pain or the alleviation of pain is mentioned in conjunction with *Pressure, Tension or Release of Pressure, Tension* double-code these two together.	Pain not attributed to meditation practice. Expected or anticipated levels of postural pain arising on account of the somatic immobility entailed in the practice of meditation and that is casually mentioned without attribution of significance or downstream consequences. Pain in the head and neck area should be coded under *Headaches*.
Parasomnias	Nightmares, vivid dreams, sleep paralysis or the alleviation of these symptoms.	Includes nightmares, vivid dreams or sleep paralysis (or experiences that resemble sleep paralysis), or the alleviation of these symptoms, attributed to meditation practice. Nightmares or vivid dreams includes either unpleasant dreams that can cause a strong negative emotional response or dreams with unusually good recall that are often perceived as if a recent experience. Sleep paralysis occurs as one falls asleep or as one wakes: an open-eyed paralysis that can last from seconds to minutes and is often accompanied by a sense of terror, danger and/or hallucinations.	Nightmares, vivid dreams or sleep paralysis or the alleviation of these symptoms attributed to a cause unrelated to meditation--for instance, cases of ongoing sleep paralysis unaffected by meditation practice. Changes in sleep amount, need, or insomnia should be coded under *Sleep Changes*.
Pressure, Tension or Release of Pressure, Tension	Bodily pressure or tension, or release of bodily pressure or tension, that can vary according to location (general or specific), intensity, or length of time.	Pressure or tension, or release of pressure or tension, attributed to meditation practice. Pressure or tension includes pressure "knots," which are sensations of intense energy in acute body locations that feel as though there are energy blockages, and may include a sensation of twisting. It also includes experiences of tightness, constriction, squeezing, contraction, locking of muscles, and other manifestations of pressure or tension. Release of pressure or tension may be described as “opening,” “relaxation” or “release,” and is sometimes but not necessarily linked to *Re-experiencing of Traumatic Memories* and may be double-coded there or with *Somatic Energy* or *Involuntary Movements*.	Pressure or tension, or release of pressure or tension, not attributed to meditation practice. *Head Pressure* connected to breathing is coded in its own category. Ordinary states of physical relaxation not characterized as a release of pressure or tension in specific areas of the body.
Sexuality-Related Changes	Hypersexuality (very frequent or suddenly increased sexual urges or activity) or hyposexuality (notably decreased sexual urges or activity).	Sexuality-related changes attributed to meditation practice. Hypersexuality can also include increased intensity of sexual urges or activity, a sense of being sexually out of control (or that one is not controlling sexual activity oneself), unwanted or disturbing thoughts that are sexual in content, or otherwise notably unusual changes related to increases in sexual urges or activity. Subjects may connect hypersexuality to “tantric” insights, to “*kundalini*” awakenings or other *Somatic Energy* experiences and effects. Thus, double-code with *Somatic Energy* where appropriate.	Sexuality-related changes not attributed to meditation practice. Descriptions of sexual energy not leading to a change in sexual desire or sexual behavior might be more appropriately coded as *Somatic Energy.*
Sleep Changes	Changes in sleep amount, sleep need, or sleep depth.	Changes in sleep amount or depth (including intensification of existing symptoms) attributed to meditation practice. Includes (but not limited to): difficulty falling asleep or staying asleep through the night, insomnia, decreased or increased sleep need, hypersomnia (excessive daytime sleepiness or prolonged nighttime sleep), or other related symptoms. May be double-coded with *Fatigue* or with *Parasomnias*.	Changes is sleep amount or depth not attributed to meditation practice. Changes in dreams, nightmares, and other anomalous sleep experiences would be coded under *Parasomnias*.
Somatic Energy	A type of sensation moving throughout the body or throughout a body area described with language of vibration, energy, current, or other related metaphors.	Somatic energy or vibrations attributed to meditation practice. Include any level of intensity (gentle to forceful, flowing to surging.) May be described as being contained within or going beyond one’s body schema. May also be reported as under one’s control or involuntary, in which case double-coding with *Loss of Sense of Agency* may be appropriate. May include use of emic terms like "*kundalini*, "*prana*," or metaphors of "currents," "voltage," or "electricity." Also includes related references to psychomotor agitation (adrenaline rushes, feeling "wired") or slowing. May also be double-coded with *Release of Tension* or with *Agitation*. Reports of somatic energy may also affect motor patterns and may therefore be closely associated with reports of *Involuntary Movements*.	Somatic energy or vibrations that are not attributed to meditation practice. The use of "energy" as a metaphysical concept without any associated phenomenology should not be coded. The term "vibrations" being used to describe minor twitches and other minor somatic movements that may also or alternatively be classified under *Involuntary Movements*.
Thermal Changes	Changes associated with heat or cold, whether a general change in sense of body temperature or localized to a specific body area.	Thermal changes attributed to meditation practice. Includes (but not limited to) statements about: sweating, dry mouth, overheating, hot flashes, prickling sensations, burning sensations, cold sweats, goosebumps, chills, hair standing on end, feeling unusually cold, or other related symptoms. Classic "*kundalini*" experiences may have heat or cold accompanied by *Somatic Energy*.	Thermal changes not attributed to meditation practice, such as changes in environmental temperature.
