# Supplementary material for: The varieties of contemplative experience: A mixed-methods study of meditation-related challenges in Western Buddhists
Source: PLoS One. 2017 May 24;12(5):e0176239. doi: 10.1371/journal.pone.0176239 (PMC5443484; doi:10.1371/journal.pone.0176239)
Supplement: S5 File — (PDF) [file pone.0176239.s005.pdf]

S5 File. Influencing Factors Codebook. Version 1.

Lindahl, J., Fisher, N., Cooper, D., Rosen, R., and Britton, W.

**Practice Domain** (5 Categories)	**Description**	**Inclusion Criteria**	**Exclusion Criteria**
Amount, Intensity, or Consistency of Practice	Changes in (whether increase or decrease) the amount of time or intensity dedicated to meditation practice, or continuing practice with the same amount of time or intensity or as before.	Any statement that mentions the amount or intensity of practice, including references to frequency, time per session, or to the intensity of practice, when attributed as a causal factor leading to difficulties or remedies. Double-code with *Intentions, Motivations or Goals* or *Worldview or Explanatory Frameworks* when these are explicitly mentioned. Practicing through difficulties would be coded here as a form of consistency of practice, potentially in conjunction with *Practice Approach, Intentions,* or *Worldview.*	A statement that relates more to intentions or beliefs about meditation than to the actual amount of time or intensity of practice. Do not code general, factual references to amount of practice that are not causally linked to meditation-related difficulties or remedies. Do not code references to effort or striving alone if they are do not include mention of corresponding impact on amount or intensity of practice. Discussion of how specific meditation techniques are engaged, such as the impact of a striving orientation, might better be coded primarily under *Practice Approach*.
Practice Approach	Concerning the practitioner's how-to knowledge about practice and the application of that knowledge.	Any explicit reference from practitioners or experts to approaches to the formal practice of meditation, whether correct or incorrect. Include postures, (mis)application of techniques, attachments to particular states, lack of how-to knowledge, excessive striving, starting or stopping practice against the instruction of the teacher, not following the directions or misunderstanding the directions. Also includes general discussion of approaches to practice deemed to be correct if they were considered causally related to difficulties or remedies. Double-coding with *Personality* or *Relationship to Teacher* may be warranted if the practitioner is doing what he/she prefers as opposed to what the teacher is suggesting or instructing. Consider double-coding with *Amount, Intensity or Consistency of Practice* when a degree of intensity, such as striving, is interpreted as an inappropriate approach to practice. When formal practice and informal practice are hard to distinguish (as in the application of mindfulness techniques in daily life), consider coding as or double-coding with *Response to Experience*.	References to general approaches to experiences that are not in the context of or consistent with formal meditation practice should be coded as *Response to Experience*. Statements referencing a type of practice or technique in itself rather than how a practitioner is applying/approaching that practice should be coded under *Type of Practice*. Double-code with *Type of Practice* when both are mentioned together. When a statement references issues of worldview or explanatory framework in an abstract way that is not explicitly connected to practice it should be coded as *Worldview or Explanatory Frameworks*.
Response to Experience	How a practitioner responds to meditation experiences that persist into daily life or how a practitioner approaches his or her experience in the context of daily life or informal practice.	References to the way in which practitioners relate to the content of their present moment experience outside of the context of formal meditation practice--in particular, how they respond to meditation-related experiences that emerge from practice and persist into daily life. On the one hand, this includes resisting, fighting, or avoiding experience. On the other hand, this also includes references to relaxing into, surrendering, impartially observing, accepting, or deidentifying with experience. References to experiential avoidance, experiential acceptance, or decentering would also be coded here. On an affective level, this includes having self-compassion or having compassion or positive affect towards elements of present moment experience; conversely, it also applies to having self-criticism or having fear, anger, or other negative affect towards present moment experience. Double-code with *Practice Approach* when the response applies both during formal and daily life/informal practice. Double-code with *Personality* when the way of relating to present moment experience is characteristic of how the practitioner responds in general and thus represents a more general psychological characteristic.	Discussion of how to practice formal meditation techniques, such as posture or settling the mind in concentration practices, or the misapplication of formal meditation techniques, should be coded as *Practice Approach*. If the instruction applies uniquely in the context of formal practice or in the application of practice techniques in an informal setting, it should be coded as *Practice Approach*. Characteristics of the practitioner that are not explicitly related to the way in which the practitioner responds to present moment experience should be coded as *Personality.* Beliefs about what is true, valuable, meaningful, or other conceptual views about the relationship between oneself and one's experience should be coded as (or double-coded with) *Worldview or Explanatory Frameworks*.
Stage of Practice	Qualities, characteristics, or issues associated with a particular meditative state or developmental stage along a contemplative path.	References to states and stages, whether positive or difficult, that are understood to be associated with certain cognitive, affective, or perceptual characteristics, but which are also generally expected to be transient in nature. Such stages may be conceived as a proximate goal of practice, an ultimate goal of practice, or a side-track from practice. Stages may be considered optional or inevitable. Stages may be specifically named and identified in the texts and traditions, or they may be more general references to a progression of contemplative development.	Statements about states or stages that pertain to psychological and not contemplative development should be coded as *Personality, Psychotherapy*, or *Worldview or Explanatory Frameworks* depending on the context. While many references to *Stage of Practice* may also include descriptions coded as Phenomenology, be sure to code *Stage of Practice* only when the stage is offered as an interpretation of experiences, or is characterized as a risk factor for experiences. In cases where attaining a subsequent stage of practice is recommended as a remedy, a double-code with *Consistency of Practice* or *Practice Approach* is likely.
Type of Practice	A specific type of contemplative practice (e.g., concentration, compassion, body scan, yoga) or a change in type of practice.	When a type of practice in itself is described as influencing the practitioner and was causally associated with challenging or difficult meditation experiences or with remedies. Includes references to specific aspects of the practice type(s). Also includes more general references to the effects of a practice tradition either in isolation or in comparison with other practice traditions. Includes reports of the impact of adding or changing a type of contemplative practice. Double-code with other relevant categories such as *Personality, Intentions, Motivations, or Goals*, or *Stage of Practice* when reasons are given for changing or a adopting a new type of practice.	References to or changes in practice amount should be primarily coded as or double-coded with *Amount, Intensity, or Consistency of Practice*. The way in which practitioners actually engage with the practices should be coded as *Practice Approach;* references to both a type of practice and the manner in which the practitioner engages with it should be double-coded with *Practice Approach.* Fasting would not be considered a type of practice but a change in *Diet.* Non-meditation practices that are part of meditation traditions, such as rituals, offerings, and other religious practices, should not be coded here but could be coded as *Other Practice* if they are given a causal attribution as a risk factor or remedy. Some practice-like activities may be better coded as in the *Health Behaviors Domain,* such as *Grounding Activity.*
**Practitioner Domain** (7 Categories)	**Description**	**Inclusion Criteria**	**Exclusion Criteria**
Identities	Demographic variables or roles that comprise a practitioner's identity and social status.	A practitioner's gender, race, ethnicity, age, religion, sexual orientation, socioeconomic status, or other aspects of identity when they have an effect on meditation practice or meditation-related experiences. Also includes roles with which practitioners have identified that impact their social relationships or behavior with meditation teachers or meditation communities. Double-code with *Sociocultural Context* when this is related to discrimination or behavior related to a particular sociocultural norm. Double-code with other codes in the *Relationship Domain* when one of these variables affects the quality of a specific relationship.	References that mention gender, age, race, ethnicity, religion, sexual orientation, or socioeconomic status but do not attribute any influence to them on meditation practice or interaction with meditation communities. References to personality traits as a form of identity would be coded under *Personality or Temperament*. References to mental illness as a form of identity would be coded under *Psychiatric History*.
Intentions, Motivations, or Goals	Intentions, motivations, ambitions, goals, and expectations in relation to contemplative practices or experiences.	Any mention of conative elements--such as wants, desires, hopes, intentions, aspirations, or motivation--in relation to practice, past or present, when these elements have an influence on meditation practices, meditation experiences, or the interpretation of or response to particular experiences. This can include goals and intentions of the meditation teacher if adopted by the individual. These can be represented as mature or naïve, or can document a shift from one set of motivations or expectations to another. Intentions will often be coded with *Worldview or Explanatory Frameworks* when there is a sufficiently explicit connection made by experts or practitioners between individual values or goals and the concepts, views, and ideals of Buddhist traditions. Includes ideal or optimal intentions, motivations, and goals put forth by experts when these are seen as having a causal role in facilitating positive or desired meditation-related experiences or responding to meditation-related challenges or difficulties.	Intentions or goals without causal attribution to or association with meditation-related experiences. Striving described solely in terms of amount or intensity of practice time, e.g. meditating long hours, would be better coded as or double-coded with *Amount, Intensity of Practice.* Views, values, and expectations without a clear conative dimension should be coded as or double-coded with *Worldview or Explanatory Frameworks.*
Medical History	Problems with physical health, whether short-term illness or chronic disease.	References to pre-existing, latent, or ongoing physical medical symptoms when these have an effect on meditation practice or meditation-related experiences. Double-code with *Psychiatric History* when a health problem has both physical and psychiatric dimensions. Include discussion by experts of medical symptoms being a risk factor.	References to medical history that are causally unrelated to meditation practice or meditation experience. Somatic challenges such as physical pain or change in breathing that are described as emerging from meditation would not be coded here, as these are Phenomenology. Psychiatric diagnoses should be coded as *Psychiatric History*. Apply *Psychotherapy and Medical Treatment* code when reference is to treatment rather than to symptoms.

Personality or Temperament	Personality characteristics, traits, dispositions, or temperament of the meditation practitioner.	Any statement which describes the personality traits or characteristics of the meditation practitioner. Personality traits, such as attachment style, that are described as stable across several years or behaviors that are stable within a single context or environment. Also include shorter term personality and temperament fluctuations that causally interact with meditation difficulties or remedies for difficulties, such as resisting meditation experiences, in which case a double-code with *Response to Experience* may be warranted. When temperament is explicitly mentioned in conjunction with meditation preferences, double-code with *Type of Practice* or *Practice Approach*.	Personality traits of other people, such as meditation teachers, would be coded in the *Relationship Domain.* References to experiences of states of being that were temporary and not integrated into or affecting the personality are more likely to be coded as Phenomenology or not coded at all. Behavioral tendencies resulting from psychiatric illness should be coded as *Psychiatric History.* General likes and dislikes should not be coded. Externally-related beliefs and orientations will likely be better coded as *Worldview or Explanatory Frameworks.* Personality traits and behavioral tendencies that developed after meditation onset would not be coded unless they are seen as remedies (such as not resisting experience).
Psychiatric History	A history of psychiatric diagnosis in the meditation practitioner that has an influence on meditation, or psychiatric diagnoses resulting from the effects of meditation.	References made to a practitioners' psych history or to mental illness among first-degree family members if an association is made with meditation practice or meditation-related experiences. In the latter case, double-coding with *Early Life Relationships* might also be warranted. Also includes references to DSM psychiatric diagnosis or reports of symptoms typical of psychiatric diagnoses that were reported or hypothesized as resulting from meditation. Includes references to taking medications related to a psychiatric diagnosis or going off of them when these result from or influence meditation practice or experience. Discussion by experts of psychological instability or psychological history as a risk factor for meditation-related challenges.	Counseling during early life period without diagnosis of psychiatric illness. References to psychiatric history that are not reported as being causally related to meditation-related difficulties should not be coded here. References to psychiatric diagnoses received after starting meditation should also not be coded if they seem completely unrelated to meditation.
Trauma History	A history of past trauma that influences the practice of meditation or specific meditation experiences.	Mention of any past event that triggered a strong emotional response characterized as traumatic or trauma-like, including after-effects such as shock, denial, flashbacks, and so forth, when such effects were attributed as being related to the practice of meditation or otherwise associated with challenging or difficult meditation experiences. These events could be accompanied by a diagnosis of PTSD, but that is not required. Discussion by experts of the putative role of trauma history on meditation experiences or on the practice of meditation, including mention of specific adjustments to practice, relationships, or supplementary treatments that need be taken into consideration for practitioners with a trauma history. In these cases, double-coding with relevant categories in *Practice, Relationship,* and *Health Behaviors Domains* is likely.	General tension in relationships with family members or others should be coded as *Early Life Relationships* or *Relationships beyond Meditation Community* depending on the time frame. Potentially traumatic events that the individual has been told about, but that are not part of the personal memory of the practitioner and do not impact the practitioner's trajectory. Traumatic events in meditation or traumatic responses to meditation that have no relationship to past experiences, such as references to a difficult period of time as "traumatic" but lacking the symptoms of traumatic re-experiencing (an acute emotional response). References to trauma that seem causally unrelated to meditation-related difficulties should not be coded.
Worldview or Explanatory Frameworks	Having, changing, or lacking an explanatory framework for how contemplative practice works, what experiences are part of the contemplative path, or beliefs about the nature of self and world.	Discussions of "view" in the specific philosophical sense, in relation to the application of contemplative practices, or in a more general sense pertaining to the practitioner's conceptions of self, teacher, world, and/or reality. Double-code with *Practice Approach* when view is thought to influence how one practices. Include references to resistance to frameworks offered by experts (teachers, psychiatrists, etc.), in which case double-code with relevant category in the *Relationship Domain* and/or *Personality.* Includes experts' views on the goals or nature of contemplative practices, especially views that are thought to be helpful or harmful when held by practitioners. Double-code with the relevant *Relationship* category when the attribution is to a broader cultural worldview that is impacting the practitioner as an individual through social relationships.	Conative dimensions--wants, goals, expectations and desires about one's practice--are coded under (or double-coded with) *Intentions*. General doctrines, dogma or explanations that are not directly associated with meditation but which influence it might be more appropriately coded as *Sociocultural Context*. Explanatory frameworks that individuals grew up with but do not actively identify with now and do not causally influence meditation approach or frameworks for understanding meditation experiences would not be coded. Changes in worldview resulting from the practice of meditation are coded as *Phenomenology*; codes here assess the influence of worldviews described as interpretations and potential risk factors or remedies.
**Relationship Domain** (6 Categories)	**Description**	**Inclusion Criteria**	**Exclusion Criteria**
Early Life Relationships	Early life relationships before adulthood (age 18) and, for practitioners, at least two years before their current age.	Any statement referencing the impact of the quality of relationships (family, friends, schoolmates, teachers, etc.) before the age of 18 or at least two years before a practitioner's current age that are associated with the trajectory of meditation practice or a specific meditation-related experience. Includes the impact of early life relationship dynamics on current relationships within the meditation community (to both teachers and other practitioners), in which case double-coding with *Relationships within Practice Community* or *Relationship to Teacher* will likely be warranted.	Childhood events unrelated to relationships and to meditation experiences should not be coded as *Early Life Relationships*. Traumatic experiences should be coded as *Trauma History*, or double-coded with *Trauma History* if the traumatic event involved a relationship (e.g., a parent or relative).
Relationships beyond Meditation Community	Relationships with family, friends, partners, and others who are not part of a meditation community, or involvement in activities beyond the meditation community that have a social dimension to them.	The quality of relationships with friends, family, and significant others, and general social relationships when such relationships influence or provide a remedy for a meditation-related difficulty. Could include references to broad affiliations of large cultural groups with which practitioners identify. Also includes general references to the causal role or importance of social environment or unspecified social communities. Includes references to supportive social relationships, to not having significant social relationships, and to conflicts with people outside of a meditation community. Becoming (increasingly or less) involved in activities that have a social dimension to them, such as dedication to or involvement in work, hobbies, or athletics, etc. when described as an influencing factor on the trajectory of meditation-related challenges.	Relationships with members of one's meditation community should be excluded unless those relationships are also family members or partners, in which case they can be double-coded with *Relationship within Meditation Community.* Change in activities with or role in a meditation community should be coded as *Phenomenology.* General statements about the influence of specific cultures or subcultures on meditation practice or experience would likely be coded as *Sociocultural Context.*
Relationships within Meditation Community	The relationship or lack thereof between the meditator and meditation community.	Any statement referencing a relationship with members of a meditation community or the qualities of such a relationship, regardless of whether there is a causal attribution to difficulties or remedies. References to structural aspects of the meditation community (such as hierarchies, access, or privileges) would also be coded here. Includes also behavioral expectations around social relationships or degree of social interaction while in the context of retreat, in which case double-coding with *Surroundings or Environment* may also be appropriate. Also includes relationships with individual members of the community and social activities engaged in as part of the community, as well as references to not having a meditation community.	References to the teacher(s) of a meditation community should be coded as *Relationship to Teacher,* unless there was a clear differentiation between a practitioner's primary teacher and other members of the community who happened also to be teachers but were not primarily related to as such. Contemplative practices undertaken as part of the meditation community should be coded in the Practice domain. Family members or partners should be excluded unless they are an important part of one's meditation community and the relationship with them reflects the general relationship with the meditation community.
Relationship to Teacher	The quality and nature of a relationship between meditation teacher and student or lack thereof.	Any statement referencing the relationship between a student and a meditation teacher, the qualities of that relationship, or the approach from either teacher or student to being in such a role. References to teaching styles, teacher personality traits, teacher views and values, and feelings towards having a teacher or not having a teacher. Includes references to assessment techniques overseen by teachers, such as screening procedures or confidentiality issues. Includes discussions of amount of or ease of access to a teacher, or their empathy/personability. Double-coding with categories in the Practice domain may be warranted when teachers prescribe approaches to practice type or amount. Double-code with *Personality or Temperament* when personality conflicts between practitioner and teacher are mentioned.	References to meditation teachers acting in other roles, such as therapist, friend, or family. References to any psychotherapeutic or medical technique that is discussed or prescribed by a teacher who is acting in the capacity as a clinician or doctor should be coded as *Psychotherapy or Medical Treatment*, but not if the teacher sees it as a part of their role as a meditation teacher.

Sociocultural Context	The influences that come from the cultural or social context of a meditation practitioner.	References to the broader cultural and/or societal context that the practitioner is within, or to contrasts between cultures or societies. References to fast-paced modern life, technology, American individualism, Asian collectivism, and the wider contexts around meditation and/or Buddhism in society. Double-code with *Personality or Temperament* when broader socio-cultural factors are adopted by an individual practitioner and influence their individual psychology. Double-code with *Worldview or Explanatory Frameworks* when a reference is made to how an individual's worldview or belief structure is shaped or influenced by larger sociocultural factors. Double-code with *Intentions, Motivations, or Goals* when there is reference to sociocultural origin of these conative dimensions.	The beliefs and worldviews that individual practitioners hold concerning the practice of meditation or the contemplative path should be coded as *Worldview or Explanatory Frameworks*. The characteristics of the immediate physical environment should be coded as *Surroundings or Environment*, whereas *Sociocultural Context* refers to the broader cultural influences that are not specific to one place or environment. Relationships with specific people should be coded as *Relationships within* or *beyond the Meditation Community*, accordingly, whereas *Sociocultural Context* refers to the broader society or culture of the practitioner.
Surroundings or Environment	The meditation practitioner's immediate physical surroundings or environment, whether stable or changing, temporary or long-term.	References to the causal role of the meditation environment on a practitioner's practice trajectory or experiences. Any statement that mentions moving to a new environment or significantly changing the old environment so that it feels different and new. Includes references to a retreat environment (e.g., silence, location, iconography) independent of the role of the people therein. Also includes references to non-practice environments when these are mentioned as having a causal influence on meditation-related experiences, in which case a double-code with the categories in the *Social Domain* of the *Phenomenology* codebook is likely.	References to a social environment or situation in terms of relationships should be coded either as *Relationship within* or *beyond Meditation Community*, accordingly. References to general aspects of American (or Asian or other) cultural life would be coded as *Sociocultural Context.*
**Health Behaviors Domain** (8 Categories)	**Description**	**Inclusion Criteria**	**Exclusion Criteria**
Bodywork or Energy Healing	Approaches to working with the physical aspects of the body as a remedy in itself or as a means of affecting the "subtle energies" of the body believed to be related to physical, mental, and emotional health.	Bodywork includes references to treatments that manipulate the physical body, such as tissue massage, musculo-skeletal alignment, acupuncture or acupressure, etc. These treatments may be presented as remedies in themselves for somatic problems or for other types of problems such as affective disturbances or ungroundedness, in which case a double-code with *Grounding Activity* may be warranted. Some physical treatments, such as acupuncture, yoga or cranio-sacral therapy, may also be presented as affecting the practitioner on an "energetic" level, referring to conceptions of the "subtle body" the as medium between the physical and mental or spiritual domains. Practices of energy healing may directly address practitioners' energetic issues or attempt to resolve somatic or other issues through the manipulation of energies.	Yoga, Tai Chi, and other potentially body or body energy-based practices could also be coded as *Exercise* or *Type of Practice*, depending on the context or intent. They should not be coded here unless the purpose is to affect healing for energy-based issues or meditation-related challenges in other domains. Double-code with *Relationship to Teacher* for healers who are also meditation teachers, or with *Psychotherapy or Medical Treatment* if the healers have a primary identity as a psychologist, psychiatrist, or medical doctor. Use of herbal remedies associated with acupuncture, Chinese medicine, or Ayurvedic medicine should be coded as *Medication*. Seeing a ritual healer would be coded here if the emphasis was on healing energetic blockages, but if the attribution is to a general effect of the ritual it should be coded as *Other Practice.*
Diet	Changes in diet, whether changes in the type of food eaten or the quantity eaten.	Changes in the quantity of food eaten or the type of food eaten. Includes discussion of fasting or change in diet type, such as becoming vegetarian or carnivorous. Includes theoretical discussion of dietary changes being a risk factor or remedy for meditation-related difficulties, or instructions by experts to alter diet (by increasing quantity of food taken, for instance) as a remedy for meditation-related challenges.	Does not include involuntary changes in diet or food intake reported in subject interviews due to meditation-related symptoms such as *Somatic Energy* or *Sleep Changes,* which are coded as *Phenomenology* under *Appetitive or Weight Changes*. If the involuntary changes in food intake are reported as a factor leading to other experiences, they should be double-coded as *Phenomenology* and as an *Influencing Factor*.
Exercise	Changes in the amount of time spent exercising, the intensity of exercise, or the type of exercise practiced.	Beginning or stopping an exercise regimen in an attempt to respond to meditation-related difficulties. Includes change in the intensity, duration, or type of exercise performed. Includes references to exercise-like physical activity such as difficult manual labor, which may warrant a double-code with *Grounding Activity*. Includes references to immobility associated with long-term or intensive practice if this is mentioned as differing from the practitioner's typical or desired amount of physical activity.	Incidental references to exercise causally unrelated to the onset of challenging meditation-difficulties or symptom reduction should not be coded. Yoga, Tai Chi or other body movement-based contemplative practices should be coded as or double-coded with *Type of Practice* or as *Bodywork or Energy Healing,* depending on the context.
Grounding Activity	Adoption of a behavior intended to ground, center, calm or embody the practitioner.	References to any activity described as "grounding" or described through similar metaphors such as centering, embodying, calming, as "grounding" can be conceptualized as an orientation towards a stable sense of embodiment. Examples include being in nature or in physical contact with the earth, such as through gardening. Also includes other menial tasks like cooking or manual labor, the adoption of particular postures, or focusing on the feet or the lower half of the body when they are described as having a "grounding" effect. Consider double-code with *Exercise* when prescriptions to walk in nature could be about the physical activity as much as the natural environment. Consider double-coding with *Diet* reports of or prescriptions to eating meat, oil, or other dietary practices attempted as grounding. Social activities described as grounding should be double-coded with the appropriate category in the Relationship domain.	References to walking outdoors should be coded as *Exercise* when the physical activity is emphasized. References to directing attention to the feet or away from other objects may be more appropriate to code as (or double-code with) *Practice Approach* or *Type of Practice*.
Medication	Ingestion of pharmacological drugs or alternative medicines, such as herbs, taken as part of a regular health regimen.	Any statement that mentions attempts at taking medication or alternative medicines, whether for meditation-related symptom reduction or for psychiatric or medical illnesses thought to have an influence on meditation-related experiences. Includes references to medications for anxiety, depression, psychosis, sleep disturbances, pain and so forth, when those symptoms were derived from meditation practice or may have influenced the trajectory of meditation-related difficulties.	Taking medication to alleviate pre-existing psychological conditions that do not recur in relation to meditation should be coded as *Psychiatric History*. References to the use of medications that seem causally unrelated to meditation-related difficulties should not be coded here, unless the practitioner makes an explicit connection in the subsequent narrative between such medications and meditation-related difficulties.
Psychotherapy or Medical Treatment	Treatment from a psychotherapist, psychologist, or a medical doctor.	Seeking support or counsel from psychotherapeutic or medical providers. Includes involuntary treatments such as inpatient hospitalization. Relationships with providers should be coded here, though subsequent implementation of technique and treatments might be better coded under *Medication* or other specific *Health Behaviors* categories. Psychotherapists or medical providers acting as such who recommend meditation, yoga, or other contemplative practices should still be coded here, though their prescriptions should be double-coded with *Type of Practice*.	References to seeking psychotherapy or medical treatment for reasons other than the alleviation of meditation-related challenges. Meditation teachers who are also doctors or psychotherapists should not be considered here if their primary relationship to the subject was as a meditation teacher, not as a health care provider. When the use of a psychotherapeutic method is seen as an act of meditation instruction or support by a meditation teacher this should be coded as *Relationship to Teacher.* Use of body and energy-based alternative medicines or healing modalities should be coded under *Bodywork or Energy Healing*.
Recreational Drugs	Ingestion of non-medical drugs or the ingestion of prescription medical drugs in a manner inconsistent with or outside of the context of a medical prescription.	Includes the ingestion of drugs such as alcohol, caffeine, marijuana, psychedelics, or the abuse of prescription drugs, where abuse means use without prescription or in a dosage beyond what was prescribed. Also includes changes (increase or decrease) in the intake of drugs, whether as a means of alleviating symptoms or coping with them. Consider double-coding with *Diet* when caffeine and alcohol are mentioned as part of daily diet.	Use of prescription drugs or other medications should be coded under *Medication* unless abused (beyond prescription-based use, dosage, justification). References to the use of drugs that seems causally unrelated to meditation-related difficulties unless the subject makes an explicit connection in the subsequent narrative between drugs and the onset or alleviation of meditation-related difficulties.
Sleep	Changes in amount of sleep needed or the amount of sleep that occurs despite a desire for more or less sleep.	Voluntary attempts to increase or decrease sleep amount as a risk factor or remedy for meditation-related difficulties. Includes references to advice given to practitioners to get more sleep to alleviate certain meditation-related symptoms. Voluntary decreases in sleep may be double-coded with or associated with *Amount or Intensity of Practice*. Consumption of sleep-aids should be double-coded with *Medication.*	Discussion of involuntary changes in sleep amount reported in practitioner interviews as arising from the practice of meditation should be coded as *Phenomenology,* unless the involuntary changes in sleep amount are reported as leading to other experiences, in which case they should be double-coded as *Phenomenology* and as an *Influencing Factor*.
